# Homo- and Heteroleptic Silylstannylenes: Synthesis, Structure and Use as Precursors to Bimetallic Compounds

**DOI:** 10.1021/acs.organomet.6c00001

**Published:** 2026-03-19

**Authors:** Aidan J. Murray, Lewis L. Wales, Maximilian Dietz, Eve M. Poland, Caitilín McManus, Agamemnon E. Crumpton, Job J. C. Struijs, Simon Aldridge

**Affiliations:** Inorganic Chemistry Laboratory, Department of Chemistry, 6396 University of Oxford, South Parks Road, Oxford OX1 3QR, U.K.

## Abstract

Two novel silyl-substituted stannylenes are reported: Ar^Mes^Sn­{Si­(SiMe_3_)_2_(Si^t^BuPh_2_)} (Ar^Mes^ = 2,6-Mes_2_C_6_H_3_, Mes = 2,4,6-Me_3_C_6_H_2_) and Sn­{Si­(SiMe_3_)_2_(Si^t^BuPh_2_)}_2_, the latter representing a very rare example of a homoleptic disilyl stannylene Together with the known stannylene Ar^Mes^Sn­{Si­(SiMe_3_)_3_}, these systems are each shown to insert into the Au–Cl bond of (Ph_3_P)­AuCl to form complexes of the type (Ph_3_P)­Au­(SnR_2_Cl) containing four coordinate stannyl ligands (where R = aryl or silyl group). Similar behavior is shown toward (Cy_3_P)­AuI, yielding crystalline (Cy_3_P)­Au­{SnI­(Ar^Mes^)­(Si­(SiMe_3_)_3_)} in the case of Ar^Mes^Sn­{Si­(SiMe_3_)_3_}. Subsequent iodide abstraction to yield [(Cy_3_P)­Au­{Sn­(Ar^Mes^)­(Si­(SiMe_3_)_3_)}]^+^ can be achieved using Li­[Al­(OR^*f*^)_4_] (R^*f*^ = −C­(CF_3_)_3_), facilitated by the long tin–halide bond and strongly electron-donating tertiary phosphine ligand. This cationic complex represents the first structurally characterized example of a simple (two-coordinate) stannylene ligand bound to a gold center in a manner analogous to classical Au­(I) carbene complexes.

## Graphical abstract

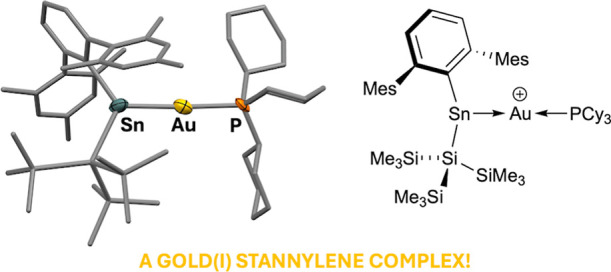

## Introduction

Stannylenes are the tin analogues of carbenes and conform to the general formula R_2_Sn, where R is typically a sterically bulky substituent which subverts aggregation.^1^ Despite the role of stable carbenes in revolutionizing transition metal coordination chemistry and its extension into homogeneous catalysis, the corresponding chemistry of their more tractable heavier group 14 analogues (including stannylenes) has received significantly less attention.^2–5^

Carbene complexes of gold (especially gold­(I)) have been widely explored in the context of bond activation, synthesis and catalysis—particularly toward systems containing C–C multiple bonds.^6–10^ Despite this, reports of related Au/Sn bimetallic compounds remain uncommon.^11–26^ An early report described the direct insertion of SnCl_2_ into triphenylphosphine gold­(I) chloride, (Ph_3_P)­AuCl, to form (Ph_3_P)­Au­(SnCl_3_), and a number of similar processes have subsequently been reported for other R_2_Sn species, accessing Au–Sn bimetallics of the general formula L_*n*_Au­(SnR_2_Cl) (where L_*n*_ is one or more neutral ligands; **I**, Scheme 1).^11–14,16,20–22,25^ The resulting bimetallic species can be thought of as featuring a strongly electron-donating formally anionic stannyl (SnX_3_^–^) ligand bound to the Au­(I) center. Reactions of gold­(I) cyanide (AuCN) with Lewis base-coordinated stannylenes have been reported to give rise to compounds of similar composition, with Au-to-Sn migration of the CN^–^ ligand being accompanied by Sn-to-Au migration of the Lewis base (to give **II**, Scheme 1).^17,18^ However, attempts to synthesize a stannylene–gold complex by abstraction of the CN^–^ ligand using B­(C_6_F_5_)_3_ resulted instead in coordination of the borane by the N-lone pair of the Sn–CN unit.^17^

**Scheme 1:**
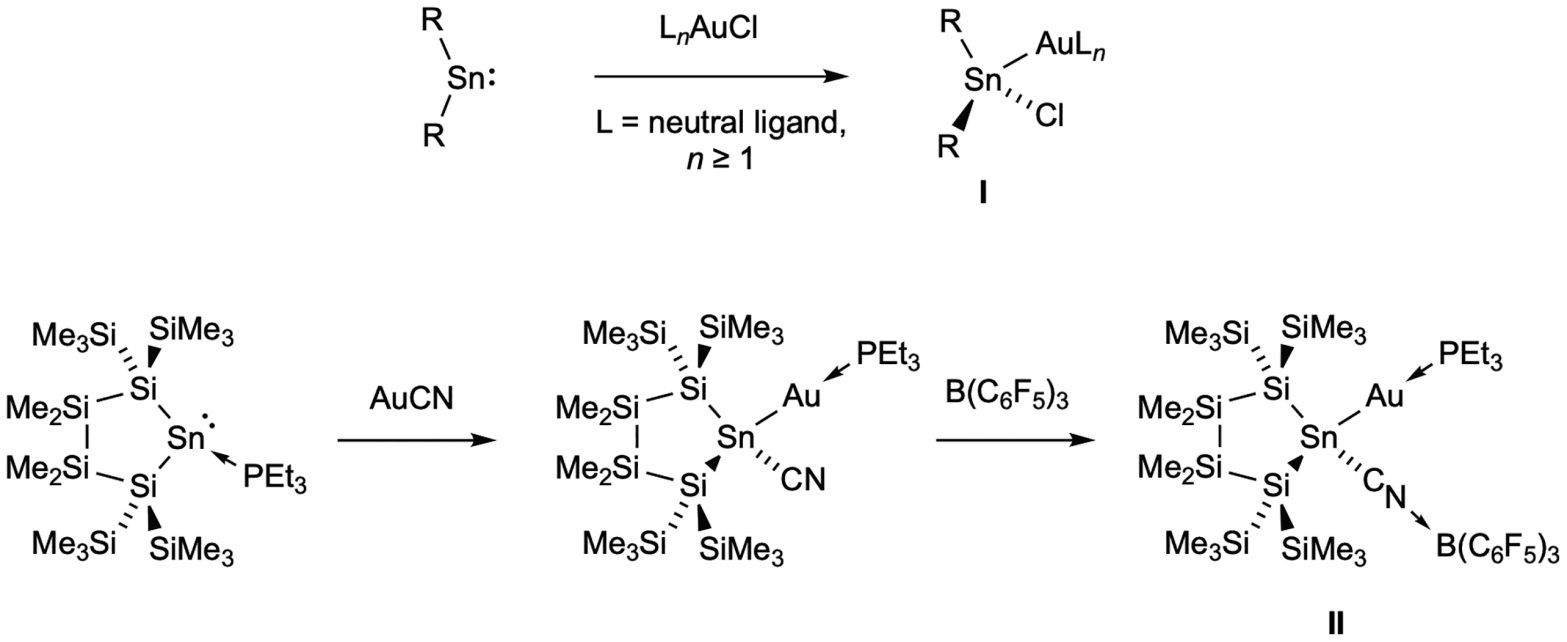
Selected Examples of Previously Reported Reactions Relevant to the Current Study

In a broader context, there are relatively few structurally characterized complexes featuring a neutral stannylene metallo–ligand coordinated to a gold center. Among cationic species, the tin­(II) center is invariably base-stabilized through coordination of neutral donor moieties tethered to the ligand framework (**III**—**VI**, Figure 1).^24,26–28^ To date (to our knowledge), there are no reported mononuclear gold complexes involving a simple two-coordinate stannylene ligand akin to commonly employed carbene donors.

**Figure 1:**
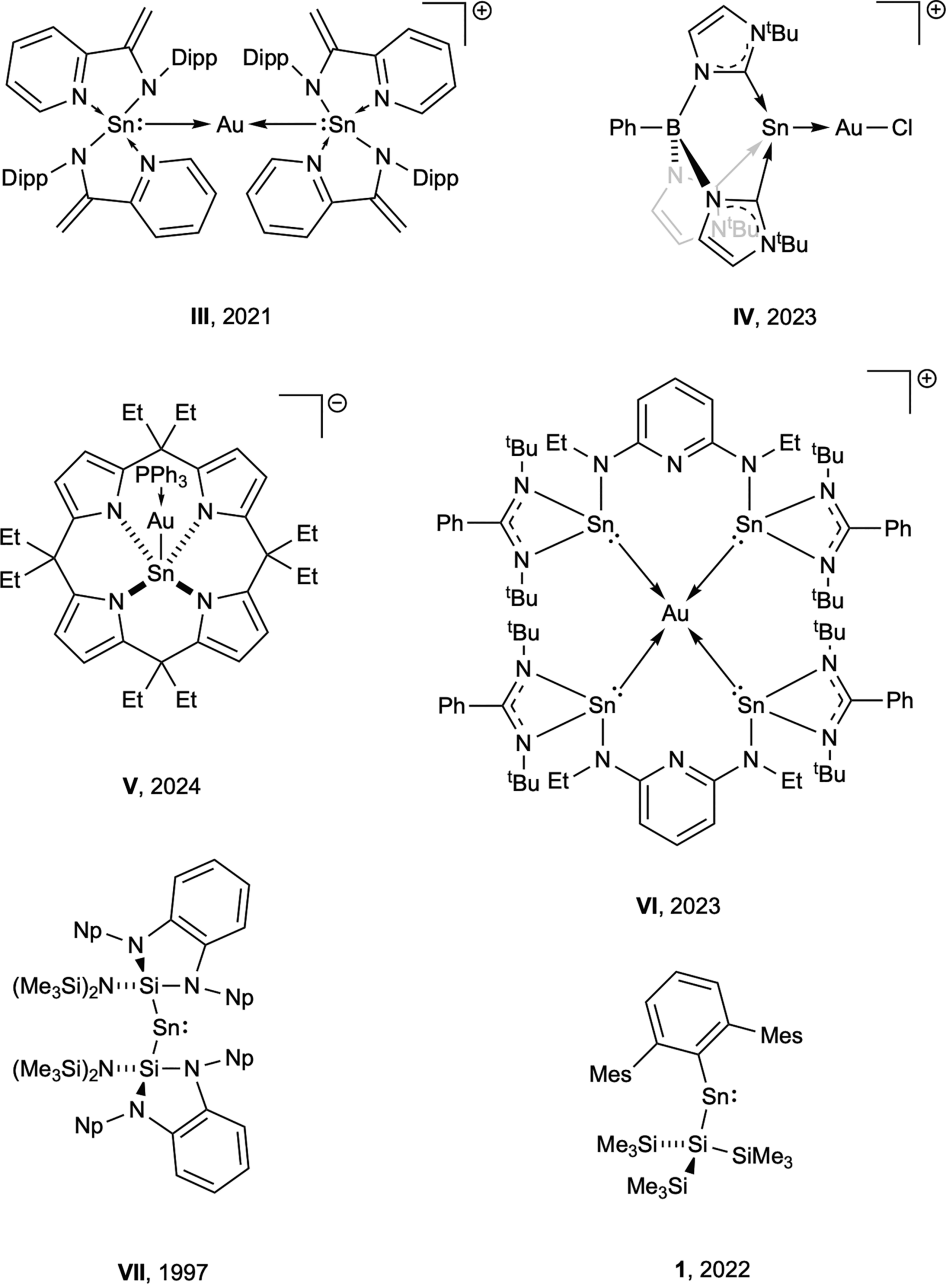
Selected examples of previously reported stannyl-gold and stannylene species relevant to the current study (counterions omitted for clarity; Dipp = 2,6-^*i*^Pr_2_C_6_H_3_, Np = CH_2_^*t*^Bu, Mes = 2,4,6-Me_3_C_6_H_2_).

The sterically bulky aryl­(silyl)­stannylene Ar^Mes^Sn­{Si­(SiMe_3_)_3_} (**1**, Figure 1; Ar^Mes^ = 2,6-Mes_2_C_6_H_3_, Mes = 2,4,6-Me_3_C_6_H_2_) has been reported previously to be highly reactive toward a range of small organic molecules, consistent with its electron-rich nature imparted (to a large degree) by the electropositive silyl substituent.^29,30^ Given the ready synthesis of **1**, we wanted to explore the potential of it (and related silyl-stannylenes), for the formation of systems directly analogous to classical gold–carbene complexes, potentially via halide abstraction from a halo–stannyl ligand (itself formed by insertion into a gold–halide bond). These studies are reported here.

## Results and Discussion

### Synthesis of Novel Stannylenes

The extremely bulky silyl substituent, −Si­(SiMe_3_)_2_(Si^*t*^BuPh_2_), has previously been developed by Schnepf and co-workers, and is accessible in straightforward fashion from the potassium “hypersilyl” compound (THF)_2_K­{Si­(SiMe_3_)_3_}.^31^ We envisaged that it might provide the basis for the facile synthesis (among other systems) of a monomeric disilyl stannylene, given its enhanced steric profile over the hypersilyl group (and the dimeric nature of [Sn­{Si­(SiMe_3_)_3_}_2_]_2_ in the solid state).

Combining equimolar amounts of Ar^Mes^SnCl and K{Si(SiMe_3_)_2_Si^*t*^BuPh_2_} in toluene solution at room temperature (Scheme 2) results in the immediate appearance of a green color, in similar fashion to that observed in the synthesis of **1**. The ^1^H NMR spectrum shows a single set of new peaks, while three environments are visible in the ^29^Si NMR spectrum (at δ_Si_ = 8.9, −3.2, −36.7 ppm). A new ^119^Sn NMR signal can be located at δ_Sn_ = 2831 ppm, i.e., at lower field than the bis­(aryl)­stannylene (Ar^Mes^)_2_Sn (δ_Sn_ = 1971 ppm), but higher than for the electron-rich bis­(boryl)­stannylene reported by Protchenko et al. (δ_Sn_ = 4755 ppm).^32,33^ To allow the connectivity to be confirmed and structural parameters to be determined crystallographically, blue crystals of **2** were grown from a hexane solution (Figure 2).

**Scheme 2:**
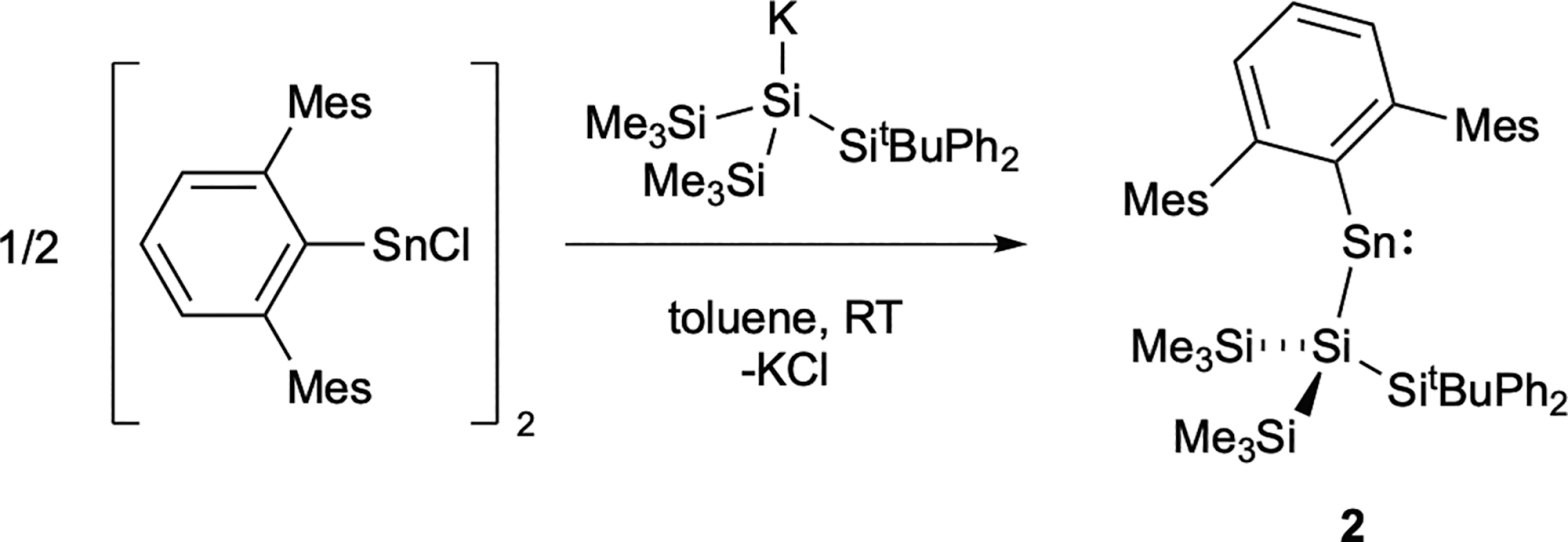
Reaction of Ar^Mes^SnCl with K­{Si­(SiMe_3_)_2_Si^*t*^BuPh_2_} to Produce Aryl­(silyl)­stannylene **2**

**Figure 2:**
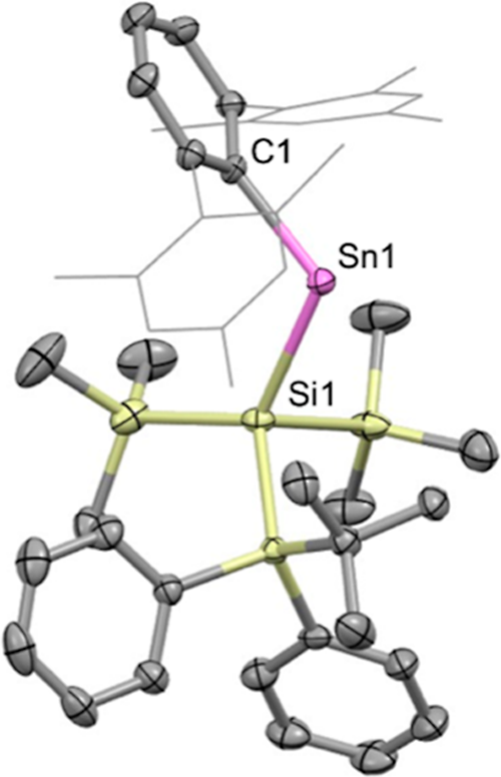
Molecular structure of **2** in the solid state as determined by X-ray crystallography. Thermal ellipsoids are set at the 50% probability level. Mes groups are depicted in wireframe format and H atoms omitted for clarity. Key bond lengths (Å), angles (°): C1–Sn1 2.220(3), Sn1–Si1 2.6807(9), C1–Sn1–Si1 113.96(8).

X-ray crystallography confirms the formulation of **2** and reveals Sn1–C1 and Sn1–Si1 distances (2.220(3) and 2.6807(9) Å, respectively) which are similar to those of **1** (2.189(3) and 2.6407(7) Å).^29^ The C1–Sn1–Si1 angle (113.96(8)°) is wider (109.75(6)° for **1**), presumably on steric grounds, but is similar both to that of the previously reported aryl­(silyl)­stannylene Ar^Mes^Sn­(Si^*t*^Bu_3_) (113.50(14)°) and the C–Sn–C angle of the bis­(aryl)­stannylene Sn­(Ar^Mes^)_2_ (114.7(2)°).^29,34,35^

With the heteroleptic aryl­(silyl)­stannylene **2** in hand, we sought to access the corresponding homoleptic disilyl stannylene Sn­{Si­(SiMe_3_)_2_(Si^*t*^BuPh_2_)}_2_. Synthetic approaches using SnCl_2_ and K­{Si­(SiMe_3_)_2_(Si^*t*^BuPh_2_)} did not yield the desired product, but a route originating from Sn­{N­(SiMe_3_)_2_}_2_ - as used for the synthesis of [Sn­{Si­(SiMe_3_)_3_}_2_]_2_ - proved more successful.^36^ The addition of Sn­{N­(SiMe_3_)_2_}_2_ to a suspension of K­{Si­(SiMe_3_)_2_(Si^*t*^BuPh_2_)} in pentane at −78 °C (Scheme 3) leads to the immediate formation of a lime-green suspension, with a further change to dark red/brown occurring on warming to 0 °C.^36^ The crude ^1^H NMR spectrum shows a single new set of resonances, while three ^29^Si NMR signals at similar shifts to those of **2** were observed to grow in (at δ_Si_ = 10.4, −3.3 and −46.0, cf. 8.9, −3.2 and −36.7 ppm, respectively). Extraction into pentane allowed the product to be isolated as deep red crystals suitable for X-ray crystallography (**3**; Figure 3). Unfortunately, a ^119^Sn NMR signal could not be unambiguously identified for **3**, which is unstable in solution at room temperature, decolorizing overnight with deposition of colloidal tin. **3** can, however, be stored as a solid under an inert atmosphere for an indefinite period of time.

**Scheme 3:**
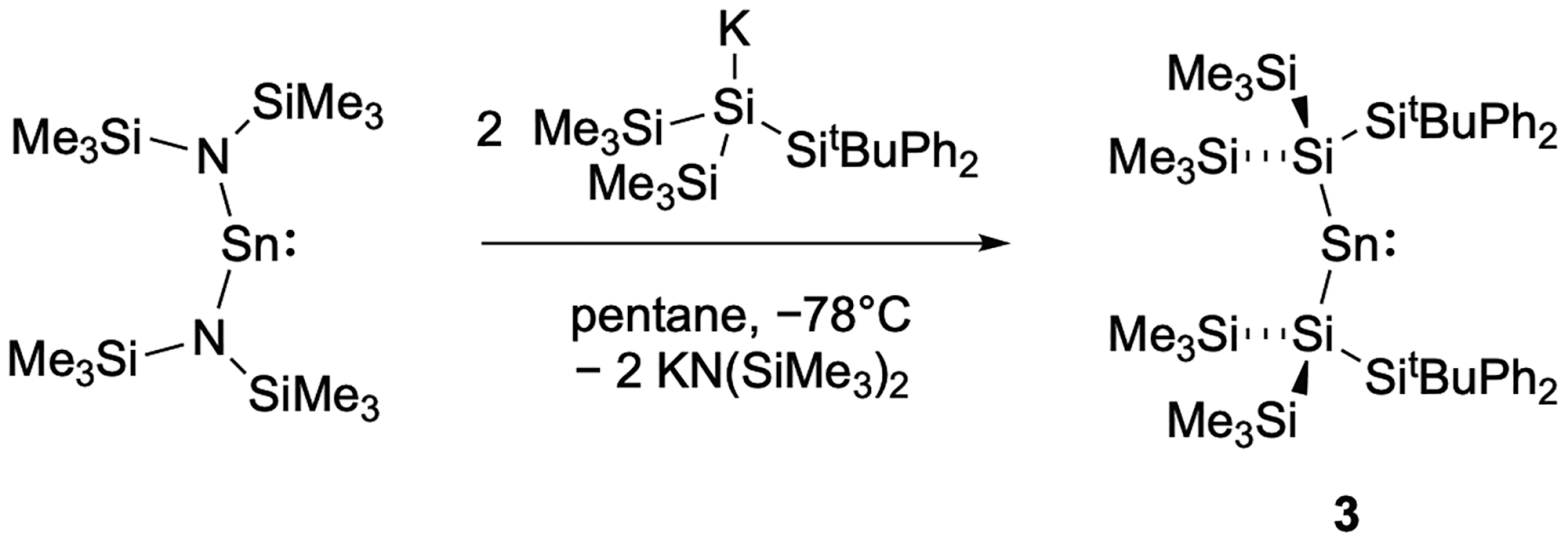
Reaction of the Bis­(amido)­stannylene Sn­{N­(SiMe_3_)_2_} with K­{Si­(SiMe_3_)_2_Si^*t*^BuPh_2_} to Give the Homoleptic Disilylstannylene Sn­{Si­(SiMe_3_)_2_(Si^*t*^BuPh_2_)}_2_, **3**

**Figure 3:**
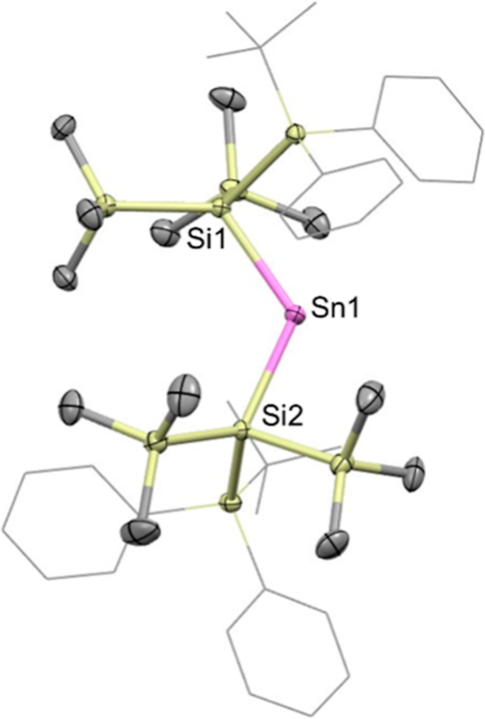
Molecular structure of **3** in the solid state as determined by X-ray crystallography. Thermal ellipsoids are set at the 50% probability level. The ^*t*^Bu and Ph groups are depicted in wireframe format and H atoms omitted for clarity. Key bond lengths (Å), angles (°): Sn1–Si1 2.6464(8), Sn1–Si2 2.6591(8), Si1–Sn1–Si2 121.71(3).

Unlike the bis­(hypersilyl)­stannylene, [Sn­{Si­(SiMe_3_)_3_}_2_]_2_, reported by Klinkhammer and co-workers, stannylene **3** is monomeric in the solid state, presumably due to the increased steric bulk of the “mega-silyl” substituents.^36^ **3** is only the second structurally characterized example of a monomeric acyclic bis­(silyl)­stannylene, the only previously reported example being formed by the insertion of a silylene precursor into both of the Sn–N bonds of Sn­{N­(SiMe_3_)_2_}_2_ (**VII**, see Figure 1). The Sn–Si distances (2.6464(8) and 2.6591(8) Å) are somewhat shorter than those reported by Lappert et al. for **VII** (both 2.712(1) Å),^37^ and the Si–Sn–Si bond angle (121.71(3)°) is much wider (cf. 106.77(5) ° for **VII**) implying a narrower HOMO–LUMO gap.^38^ This angle is also wider than that measured for **2** (113.96(8)°), and for the above bis­(boryl)­stannylene (118.8(3)°).^33^ It is, however, narrower than the C–Sn–C angle measured for the bis­(aryl)­stannylene Sn­(Ar^iPr,Mes^)_2_ (Ar^iPr,Mes^ = 2,6-Mes_2_-3,5-^*i*^Pr_2_C_6_H_3_) reported by Power and co-workers (C–Sn–C angle 123.4(2)°).^38^

### Reactions of Silylstannylenes with (Ph_3_P)­AuCl

With novel stannylenes **2** and **3** in hand, we sought to investigate their reactivity toward Au­(I) halide precursors. Combining equimolar amounts of **1** and (Ph_3_P)­AuCl in toluene solution results in the immediate disappearance of the green color of **1** and the formation of a pale-yellow solution. The ^1^H NMR spectrum of the reaction mixture shows quantitative conversion to one product, as revealed by a single set of proton environments; restricted rotation of the mesityl substituents is implied by the presence of three sharp 6H singlets (at δ_H_ = 2.43, 2.36, and 2.16 ppm), as compared to the broad overlapping signal measured for the Mes *o*-Me group of **1** (δ_H_ = 2.41–2.37 ppm).^29^ The ^31^P NMR spectrum of the product shows a signal at δ_P_ = 43.6 ppm with ^119/117^Sn satellites. This shift is within the range for the ^31^P NMR resonances reported for structurally characterized compounds containing a Ph_3_P–Au–Sn linkage (δ_P_ = 62.6–31.0 ppm),^13,22,28^ and the ^119/117^Sn coupling constants (^2^ *J*_Sn–P_ = 1890 and 1800 Hz, respectively), fall within the range of ^2^ *J*_Sn–P_ values reported for phosphine–Au–Sn systems (1240–3430 Hz).^13,17,22,28^ The ^119^Sn NMR signal appears as a doublet at δ_Sn_ = 272 ppm (with a matching coupling constant, ^2^ *J*_Sn–P_ = 1890 Hz), and falls in the range for reported stannyl–gold complexes (δ_Sn_ = −126 to 530 ppm).^17,18,22,26,27^ On this basis, we hypothesized that **1** has undergone insertion into the Au–Cl bond of (Ph_3_P)­AuCl (Scheme 4). Colorless crystals of product **4** were obtained from a hexane solution and allow this proposal to be confirmed (Figure 4).

**Scheme 4:**
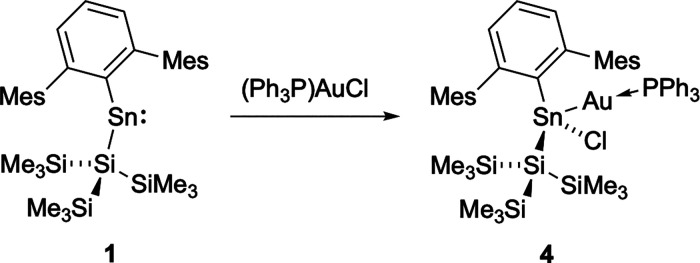
Reaction of **1** and (Ph_3_P)­AuCl to Form Stannyl–Gold Product **4** by Insertion Into the Au–Cl Bond

**Figure 4:**
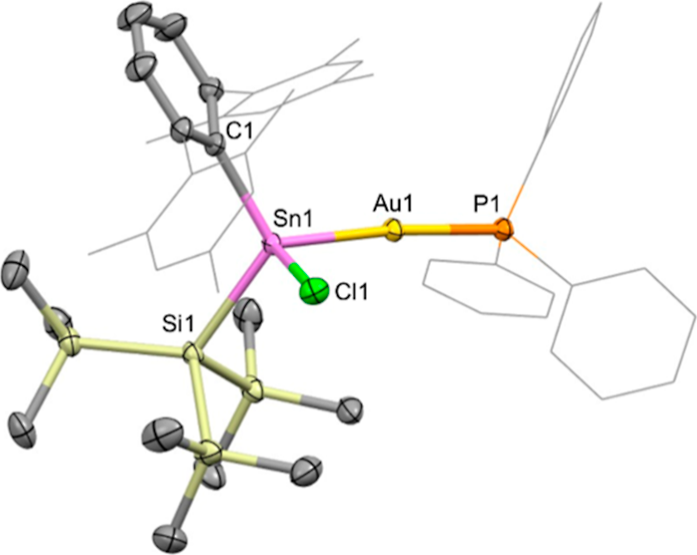
Molecular structure of **4** in the solid state as determined by X-ray crystallography. Thermal ellipsoids are set at the 50% probability level. Mes and Ph groups are depicted in wireframe format and H atoms omitted for clarity. Key bond lengths (Å), angles (°): Sn1–Au1 2.5915(4), Au1–P1 2.3268(5), Sn1–C1 2.201(2), Sn1–Si1 2.6575(6), Sn1–Cl1 2.4148(6), Sn1–Au1–P1 174.48(2), C1–Sn1–Si1 116.97(5), C1–Sn1–Au1 117.61(5), C1–Sn1–Cl1 103.22(5), Si1–Sn1–Au1 113.16(2), Si1–Sn1–Cl1 100.99(2), Au1–Sn1–Cl1 101.17(2).

The gold center in **4** shows the expected linear coordination, with a Sn1–Au1–P1 angle of 174.48(2)°, and the associated Sn1–Au1 bond distance (2.5915(4) Å) falls within the range for previously reported linear P–Au–Sn motifs (2.5438(9)–2.6141(2) Å).^13,14,17,18,21,22,24^ The Au1–P1 distance of 2.3268(5) Å is also within the range associated with phosphine–Au–Sn precedent.^13,17,21,22,28^ The C1–Sn1–Si1 bond angle (116.97(5)°) is significantly wider than that of the starting material **1** (109.75(6)°), though the Sn1–C1 and Sn1–Si1 bond lengths (2.201(2) and 2.6575(6) Å, respectively), remain very similar (cf. 2.189(3) and 2.6407(7) Å for **1**).^29^

Insertion in the same fashion occurs with stannylenes **2** and **3** (Scheme 5); combination of either stannylene with an equal amount of (Ph_3_P)­AuCl in toluene solution results in the immediate disappearance of the strongly colored starting material. The in situ ^1^H NMR spectrum in each case reveals quantitative conversion to a single novel product, in which rotation of the SiMe_3_ groups appears to be restricted on the NMR timescale. Each new compound is characterized by two distinct singlets of equal intensity in the silyl Me region (at δ_H_ = 0.37 and 0.05 ppm and δ_H_ = 0.50 and 0.49 ppm, respectively). The ^31^P NMR spectra show new resonances at δ_P_ = 45.2 and 46.6 ppm respectively, with ^119/117^Sn satellites (^2^ *J*_P–Sn_ = 1800 and 1700 and ^2^ *J*_P–Sn_ = 1390 and 1330 Hz, respectively). The corresponding ^119^Sn NMR spectra show doublets centered at δ_Sn_ = 260 (*J*_Sn–P_ = 1800 Hz) and 281 ppm (*J*_P–Sn_ = 1400 Hz), respectively, i.e. very similar to **4** (δ_P_ = 43.6 ppm, ^2^ *J*_Sn–P_ = 1890 and 1800 Hz; δ_Sn_ = 272, ^2^ *J*_Sn–P_ = 1890 Hz). Colorless crystals of the products **5** and **6** were obtained from solutions in hexane, allowing the respective structures to be determined crystallographically (Figure 5).

**Scheme 5:**
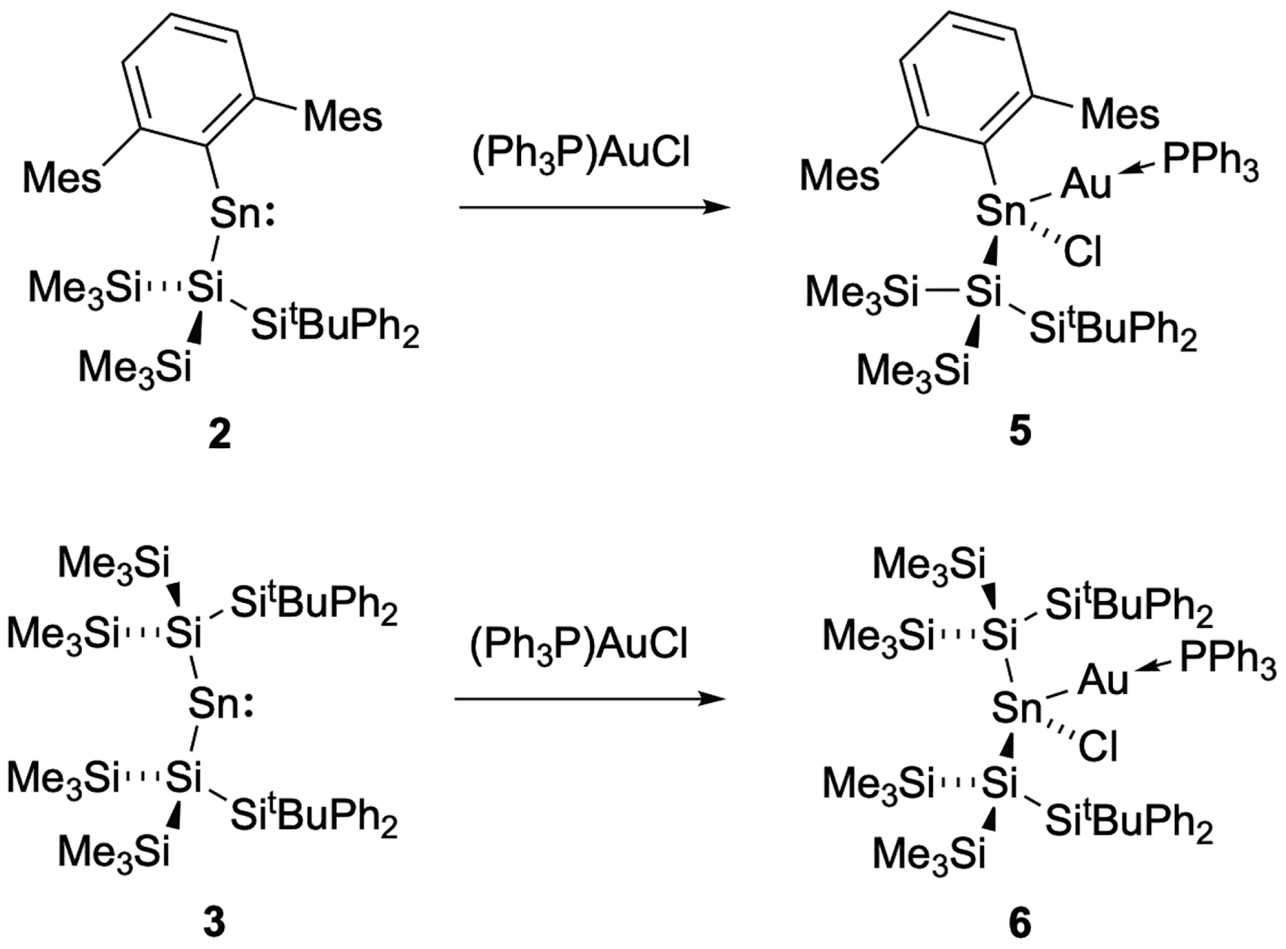
Reaction of Stannylenes **2** and **3** with (Ph_3_P)­AuCl to Form Insertion Products **5** and **6**, Respectively

**Figure 5:**
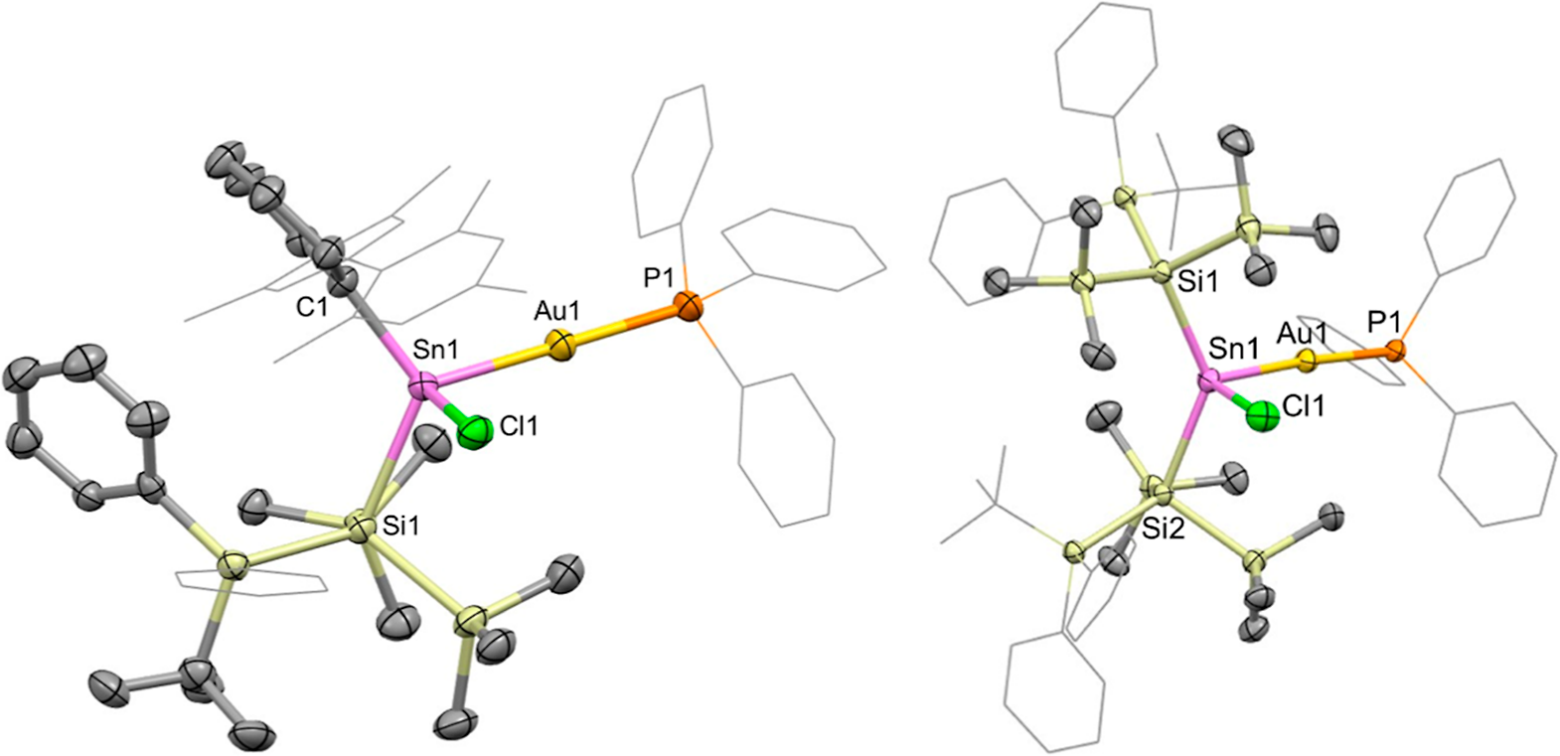
Molecular structures of **5** (left) and **6** (right) in the solid state as determined by X-ray crystallography. Thermal ellipsoids are set at the 50% probability level. Mes, ^*t*^Bu and selected Ph groups are depicted in wireframe and H atoms omitted for clarity. Key bond lengths (Å) and angles (°) for **5**: Sn1–Au1 2.5806(5), Au1–P1 2.331(2), Sn1–C1 2.216(6), Sn1–Si1 2.657(2), Sn1–Cl1 2.428(2), Sn1–Au1–P1 175.52(5), C1–Sn1–Si1 120.8(2), C1–Sn1–Au1 114.2(2), C1–Sn1–Cl1 105.2(2), Si1–Sn1–Au1 117.12(4), Si1–Sn1–Cl1 95.08(5), Au1–Sn1–Cl1 97.97(4). For 6: Sn1–Au1 2.6120(6), Au1–P1 2.341(1), Sn1–Si1 2.664(1), Sn1–Si2 2.677(1), Sn1–Cl1 2.448(1), Sn1–Au1–P1 17.97(4), Si1–Sn1–Si2 136.37(4), Si1–Sn1–Au1 112.51(3), Si1–Sn1–Cl1 97.18(5), Si2–Sn1–Au1 104.56(3), Si2–Sn1–Cl1 97.00(5), Au1–Sn1–Cl1 101.75(4).

X-ray crystallography confirms connectivity analogous to that of **4**: both **5** and **6** possess a linear coordination geometry around Au1, with Sn1–Au1–P1 angles of 175.52(5) and 175.97(4)°, respectively. **5** features Sn1–Au1 and Au1–P1 distances (2.5806(5) and 2.331(2) Å, respectively) which are similar to **4** (2.5915(4) and 2.3268(5) Å, respectively). Those of **6** are marginally longer (Sn1–Au1, 2.6120(6); Au1–P1, 2.341(1) Å, respectively), presumably due to the steric and electronic effects of the two silyl substituents. Consistently, a widened Si1–Sn1–Si2 bond angle of 136.37(4)° is also observed for **6** (cf. 121.71(3)° for **3**).

Similar experiments involving triphenylphosphine copper­(I) and silver­(I) chlorides ((Ph_3_P)­MCl, where M = Cu, Ag) resulted in decomposition in all cases, in contrast to their corresponding reactions with SnCl_2_, which generate compounds of type (Ph_3_P)­M­(SnCl_3_).^11^ It appears that, of the coinage metals, only gold is sufficiently electronegative (χ = 2.54, cf. χ = 1.90, 1.93 for Cu and Ag) to accommodate the additional electron density originating from the more electron-releasing stannyl ligands generated here.^39^

### Halide Abstraction: Reactions of Silylstannylenes with (Cy_3_P)­AuI

With a view to probing the possibility for generating cationic Au­(I) stannylene complexes by subsequent halide abstraction, the reactions of silyl stannylenes with tricyclohexylphosphine gold­(I) iodide, (Cy_3_P)­AuI, were investigated, reasoning that the more weakly bound iodide substituent and the more strongly electron donating phosphine ligand would promote halide loss (mixtures of products being observed with compounds **4** and **6**).

As in the case of compound **4**, combining equal amounts of **1** and (Cy_3_P)­AuI in toluene solution immediately yields a pale-yellow solution. A single new set of signals is observed in the ^1^H NMR spectrum, and the ^31^P NMR spectrum shows one new resonance (at δ_P_ = 62.7 ppm) with ^119/117^Sn satellites (*J*_Sn–P_ = 1880, 1800 Hz); the corresponding ^119^Sn NMR spectrum shows a new doublet centered at δ_Sn_ = 177 ppm (J_Sn–P_ = 1880 Hz). These data are closely comparable to those measured for **4**, **5** and **6**, and colorless crystals of product **7** were obtained from hexane to confirm stannylene insertion into the Au–I bond (Scheme 6 and Figure 6).

**Scheme 6:**
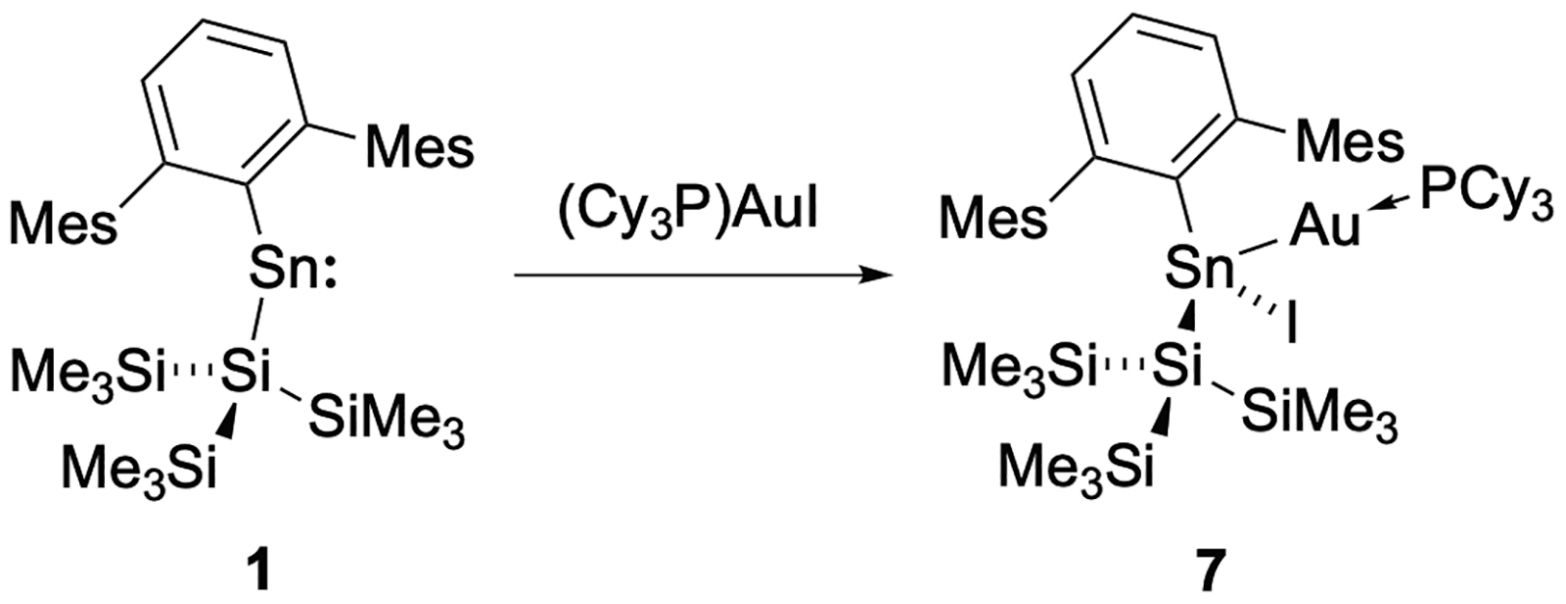
Reaction of **1** with (Cy_3_P)­AuI to Form Product **7** by Insertion Into the Au–I Bond

**Figure 6:**
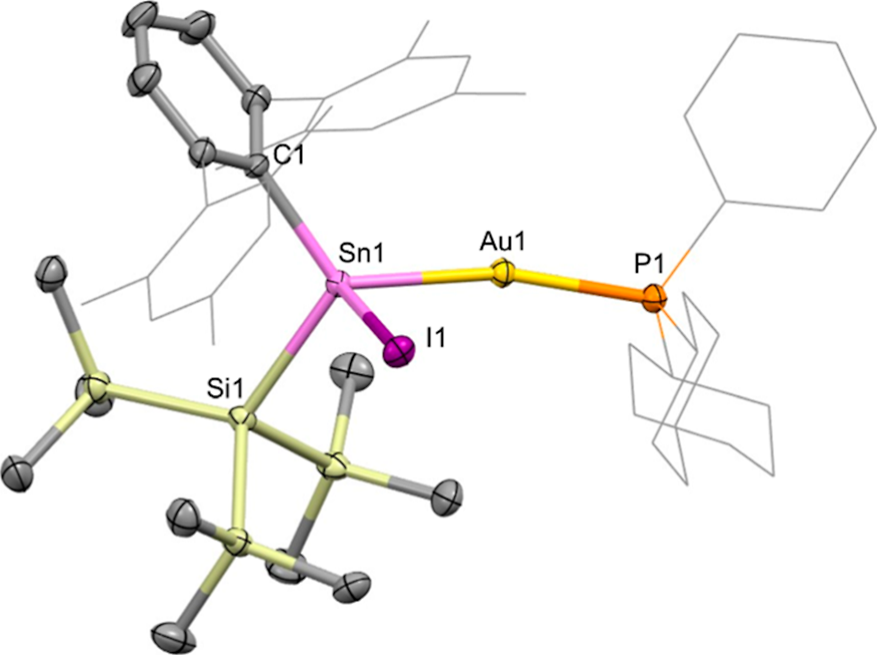
Molecular structure of **7** as determined by X-ray crystallography. Thermal ellipsoids are set at the 50% probability level. Hydrogen atoms are omitted for clarity. The Mes and Cy groups are depicted in wireframe for clarity. Key bond lengths (Å), angles (°): Sn1–Au1 2.5870(5), Au1–P1 2.320(1), Sn1–C1 2.195(4), Sn1–Si1 2.649(1), Sn1–I1 2.7941(6), Sn1–Au1–P1 162.82(3), C1–Sn1–Si1 112.0(1), C1–Sn1–Au1 122.9(1), C1–Sn1–I1 107.5(1), Si1–Sn1–Au1 117.95(3), Si1–Sn1–I1 101.46(3), Au1–Sn1–I1 88.49(2).

The solid-state structure of **7** reveals Sn1–Au1 and Au1–P1 bond lengths of 2.5870(5) and 2.320(1) Å, respectively, i.e. similar to those of **4** (2.5915(4) and 2.3268(5) Å). The Sn1–Au1–P1 angle on the other hand (162.82(3)°) shows significantly more pronounced distortion from linearity (cf. 174.48(2), 175.52(5) and 175.97(3)° for **4**—**6**), presumably due to the enhanced steric profile of the cyclohexyl groups—which are brought into contact with one of the flanking Ar^Mes^ Mes substituents.

Stannylenes **2** and **3** also react with (Cy_3_P)­AuI, yielding products that could be characterized in situ by similar spectroscopic signatures to **7**. As such, the respective ^31^P NMR spectra feature new signals at δ_P_ = 60.7 ppm (*J*_Sn–P_ = 1810, 1730 Hz for ^119/117^Sn) and δ_P_ = 60.3 ppm (*J*_Sn–P_ = 1410, 1340 Hz). These coupling constants are very similar to those measured for **5** and **6** (^2^ *J*_Sn–P_ = 1800, 1700 and ^2^ *J*_Sn–P_ = 1390, 1330 Hz, respectively) and the chemical shifts are offset from **5**/**6** by *ca*. 15 ppm (δ_P_ = 45.2, 46.6 ppm, respectively) by virtue of the different tertiary phosphine employed. While these data are consistent with reactivity occurring via similar Au–I insertion processes, the former product (derived from **2**) was found to decompose to a metallic mirror within a matter of minutes at room temperature. The product derived from **3** is more stable in solution, but despite multiple attempts, X-ray quality single crystals could not be obtained.

Further examination of the crystal structure of **7** reveals that C1, Si1 and Au1 define a much closer to trigonal planar arrangement around Sn1 than is the case with **4** (which features the same tin-bond silyl and aryl substituents): the sum of the respective angles is 352.9(1)° for **7**, compared to 347.7(1)° for **4**. This geometric arrangement, together with the relative length and weakness of the Sn–I bond suggested that **7** might be an ideal substrate for halide abstraction to generate a gold complex featuring a two-coordinate stannylene ligand. Accordingly, addition of one equivalent of Li­[Al­(OR^*f*^)_4_] (R^*f*^ = – C­(CF_3_)_3_), to a solution of **7** in *ortho*-difluorobenzene (*o*-DFB) resulted in an immediate color change to red, with accompanying formation of a colorless precipitate.^40^ In situ analysis by ^31^P NMR spectroscopy showed the formation of a new resonance at δ_P_ = 71.5 ppm (with separate ^119/117^Sn satellites unresolved; ^2^ *J*_Sn–P_ = *ca*. 1360 Hz), and single crystals of the product, [(Cy_3_P)­Au­{Sn­(Ar^Mes^)­(Si­(SiMe_3_)_3_)}]­[Al­(OR^*f*^)_4_] (**8**), could be obtained from a concentrated solution in *o*-DFB layered with hexane and stored at room temperature for 48 h (Scheme 7 and Figure 7).

**Scheme 7:**
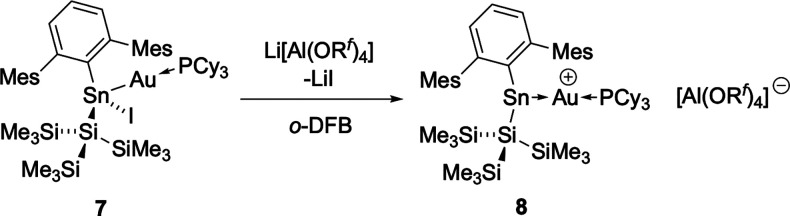
Iodide Abstraction from **7** with Li­[Al­(OR^*f*^)_4_] to Form [(Cy_3_P)­Au­{Sn­(Ar^Mes^)­(Si­(SiMe_3_)_3_)}]^+^ (**8**, as the [Al­(OR^*f*^)_4_]^−^ Salt)

**Figure 7:**
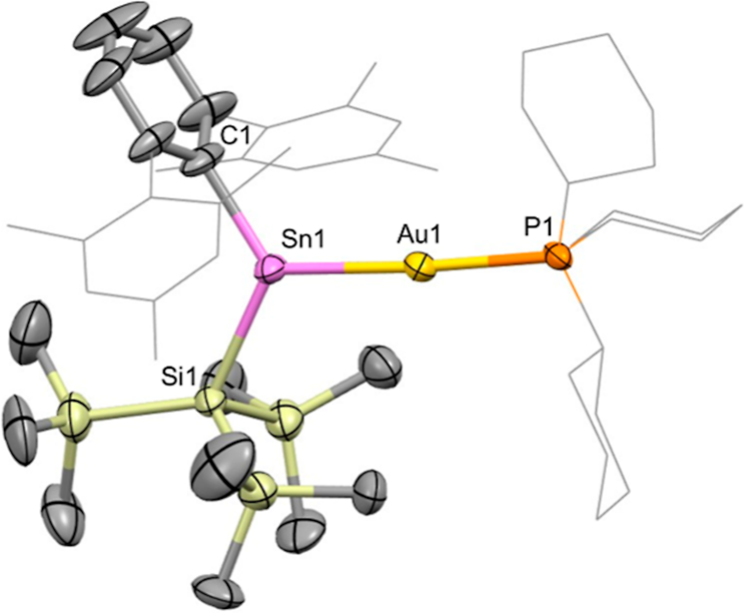
Molecular structure of the cationic component of **8** in the solid state as determined by X-ray crystallography. Thermal ellipsoids are set at the 50% probability level. Hydrogen atoms are omitted for clarity. The Mes and Cy groups are depicted in wireframe for clarity. Key bond lengths (Å), angles (°): Sn1–Au1 2.5761(13), Au1–P1 2.382(3), Sn1–C1 2.19(3), Sn1–Si1 2.568(4), Sn1–Au1–P1 176.91(8), C1–Sn1–Si1 117.0(9), C1–Sn1–Au1 125.5(9), Si1–Sn1–Au1 117.35(8).

X-ray crystallography confirms the abstraction of iodide from charge-neutral **7**, to form the well-separated ion pair [(Cy_3_P)Au­{Sn­(Ar^Mes^)­(Si­(SiMe_3_)_3_)}]^+^[Al­(OR^*f*^)_4_]^−^ (**8**). To the best of our knowledge **8** features the first example of a simple two-coordinate stannylene bound to a gold center.^41,42^ The C1, Si1 and Au1 atoms define a trigonal planar array around Sn1, with the sum of the respective angles equal to 359.6(7)°. The Sn1–Au1–P1 angle is much closer to linear than in **7** (176.91(8)°, cf. 162.82(3)°), presumably due to relief of steric crowding at tin; interestingly though, the Sn1–Au1 and Au1–P1 distances (2.5761(13) and 2.382(3) Å, respectively) remain largely unchanged compared to stannyl complex **7** (2.5870(5) and 2.320(1) Å). By means of comparison with related carbene–gold complexes featuring trialkyl phosphines, the series of cations [(IDipp)­Au­(PR_3_)]^+^ (R = Cy, ^*n*^Bu, ^*t*^Bu; IDipp = C­{(NDipp)­CH}_2_) possess Au–P distances which are shorter than that of **8** (2.279(1)—2.314(2) Å, cf. 2.382(3) Å).^43,44^ In the absence of appreciable steric effects (as implied by the near-linear Sn–Au–P angle), the longer bond length associated with the *trans* PCy_3_ ligand in **8** suggests a greater degree of σ donation from the more electropositive heavier metallylene ligand over its IDipp carbene counterpart (i.e., a greater *trans* influence).

The ^119^Sn NMR signal for **8** is a doublet centered at δ_Sn_ = 1816 ppm (^2^ *J*_Sn–P_ = 1360 Hz), i.e., at a chemical shift closer to the “free” aryl­(silyl)­stannylene **2** (δ_Sn_ = 2831 ppm),^45^ and significantly downfield both from the starting material **7** (δ_Sn_ = 177 ppm) and from the range of previously reported gold stannyl complexes (δ_Sn_ = −126 to 530 ppm). Structurally, the C–Sn–Si angle measured for the cationic component of **8** (117.0(9)°) is markedly wider than that measured for the “free” parent stannylene **1** (109.75(6)°),^29^ implying significantly enhanced p-orbital character in the Sn-centered lone pair. This in turn might be expected to contribute to the reduced magnitude of the ^2^ *J*_SnP_ coupling constant measured for **8** (ca. 1360 Hz, mean for ^119/117^Sn) compared, for example, to its stannyl precursor **7** (1880/1800 Hz).

To better understand the bonding in the novel cationic component of compound **8** we carried out a range of quantum chemical analyses, making use of NBO, ELF and EDA-NOCV approaches (see Supporting Information). Energy decomposition analysis (EDA–NOCV) reveals that the Au–Sn interaction is dominated by a single, well-defined donor–acceptor channel. The principal NOCV pair (Δ*E*_1_ = −79.5 kcal mol^–1^; |ν_1_| = 0.81 e; Figure 8) accounts for essentially the entirety of the orbital interaction, with the remaining channels contributing only marginal stabilization (ca. −3 to −4 kcal mol^–1^). The deformation density associated with pair 1 clearly shows charge flow from the Sn fragment toward the cationic Au fragment, consistent with donation of the Sn lone pair into the empty acceptor orbital of Au­(I). The corresponding NOCV orbitals confirm this assignment and display bonding and antibonding combinations characteristic of Sn → Au σ-donation. The magnitude of this interaction is substantially greater than that of any other orbital contribution, establishing it as the dominant component of the Au–Sn bond.

**Figure 8:**
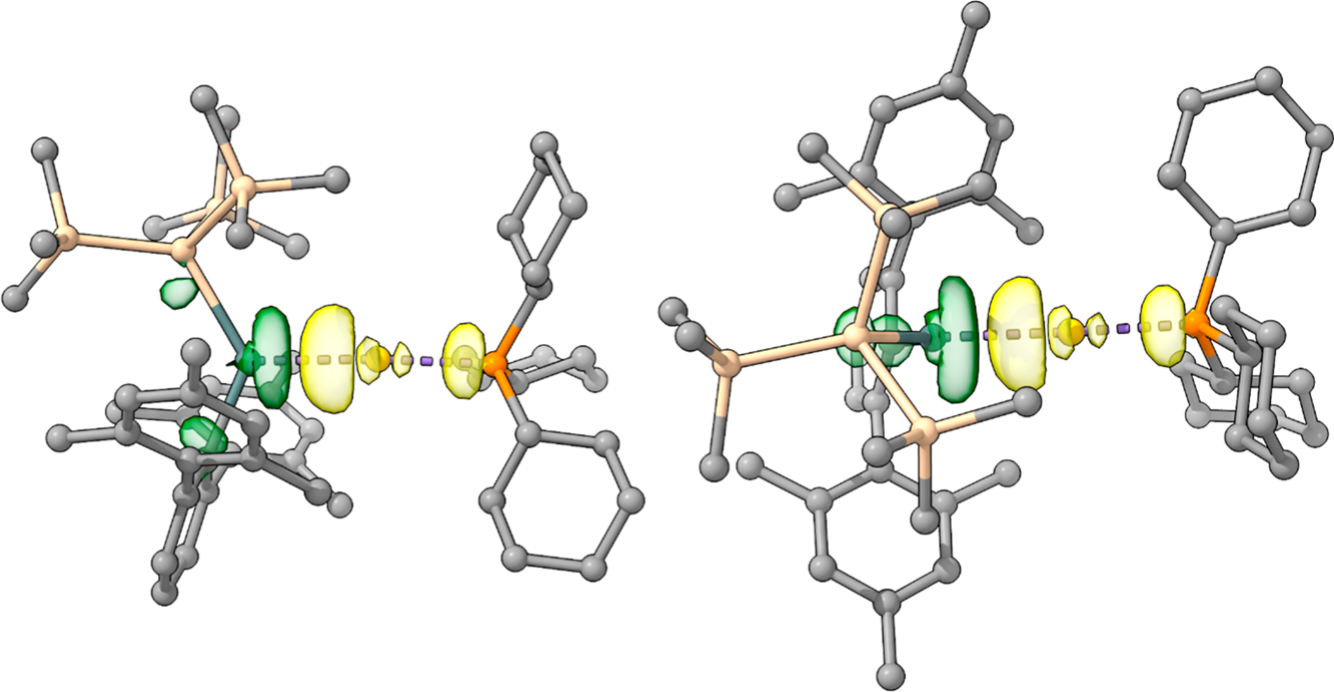
NOCV pair 1 deformation density (Δ*E* = −79.47 kcal mol^–1^), plotted at an isovalue of ±0.005. Electron density flows from green to yellow, corresponding to a charge transfer of 0.81 e.

The frontier molecular orbitals of [(Cy_3_P)­Au­{Sn­(Ar^Mes^)­(Si­(SiMe_3_)_3_)}]^+^ are consistent with this donor/acceptor description. The HOMO (−6.54 eV) shows significant contributions from the Sn and P centers, while the LUMO (−4.22 eV) is predominantly localized at Sn and corresponds to a vacant p-type acceptor orbital (Figures S42 and S43). Although minor Au *d*-character is present in the LUMO, the NOCV analysis demonstrates that back-donation into this orbital is energetically insignificant. Natural bond orbital (NBO) analysis further supports a polarized σ-bonding interaction. The Au–Sn bonding NBO (occupancy = 1.87 e) is composed of 42% Au character (predominantly s-type) and 58% Sn character. This is consistent with donation from a lone pair at Sn into an acceptor orbital of predominantly Au s-character, as expected for a *d* ^10^ Au­(I) center. The NBO analysis additionally identifies a 3-center, 4-electron Sn–Au–P hyper-bond (occupancy = 3.84 e), linking the Sn–Au σ-bond and the P-centered lone pair. This hyper-bond description is consistent with the delocalized character observed in the NOCV orbitals and the HOMO. Topological analysis of the electron localization function (ELF) reveals disynaptic basins associated with the Au–Sn interaction containing approximately two electrons in total, consistent with a conventional σ-bond. The relatively low bifurcation point for the Au–Sn basin suggests a polarized bond with limited covalent character, in agreement with the strongly donor–acceptor nature indicated by EDA–NOCV.

Overall, the Au–Sn bond is therefore best described as a predominantly σ-donor interaction arising from lone pair donation at Sn into a cationic Au­(I) acceptor orbital, with minimal backdonation. The interaction is therefore largely of L-type donor character, exhibiting significant polarization toward Sn and limited π-contribution from Au.

## Conclusions

Two novel silyl-substituted stannylenes have been synthesized via silyl for halide/amide metathesis, in one case demonstrating the first use of this conceptually simple methodology to generate a disilyl stannylene. These systems have all been shown to be capable of insertion into the Au–Cl bond of (Ph_3_P)­AuCl to form the related gold chlorostannyl complexes, although the strongly electron-donating properties of the silyl substituent appear to rule out analogous processes with the less electronegative group 11 metals copper and silver. The corresponding chemistry carried out with (Cy_3_P)­AuI generates related products derived from insertion into the Au–I bond. In the case of (Cy_3_P)­Au­{SnI­(Ar^Mes^)­(Si­(SiMe_3_)_3_)}, subsequent iodide abstraction generates the ion pair [(Cy_3_P)­Au­{Sn­(Ar^Mes^)­(Si­(SiMe_3_)_3_)}]^+^[Al­(OR^*f*^)_4_]^−^, containing the first example of a simple (non base-stabilized) stannylene coordinated to a gold center.

## Experimental Section

Complete synthetic and characterizing data for all novel compounds, representative spectra, and details of crystallographic studies are included in the Supporting Information. CIFs relating to the X-ray crystal structures of compounds **2**—**8** have been deposited with the Cambridge Crystallographic Data Centre (CCDC), reference numbers: 2518158—2518164.

## Supplementary Material





## Notes

Single file (pdf) containing synthetic procedures, characterizing data, representative spectra, crystallographic details and details of quantum chemical calculations (PDF)
xyz coordinates for calculated structures (XYZ)
