# Supplementary material for: Homo- and Heteroleptic Silylstannylenes: Synthesis, Structure and Use as Precursors to Bimetallic Compounds
Source: Organometallics. 2026 Mar 19;45(7):828–36. doi: 10.1021/acs.organomet.6c00001 (PMC13081111; doi:10.1021/acs.organomet.6c00001)
Supplement: Supplementary file 1 [file om6c00001_si_001.pdf]

Supporting Information

Homo- and Heteroleptic Silylstannylenes: Synthesis, Structure and Use as Precursors to Bimetallic Compounds

Aidan J. Murray, Lewis L. Wales, Maximilian Dietz, Eve M. Poland, Caitilín McManus, Agamemnon Crumpton, Job J. C. Struijs and Simon Aldridge*

Inorganic Chemistry Laboratory, Department of Chemistry, University of Oxford, South Parks Road, Oxford, OX1 3QR (UK)

simon.aldridge@chem.ox.ac.uk

Supporting Information (46 pages):

1. General Considerations S2
2. Syntheses of Precursors S3
3. Syntheses of Novel Compounds S4
4. X-ray Crystallographic Details S8
5. NMR Spectra of Novel Compounds S10
6. Details of Quantum Chemical Calculations S27
7. References S35

1. General Considerations

All manipulations were performed using standard Schlenk line or dry-box techniques under an argon or nitrogen atmosphere. Solvents were degassed by sparging with argon and dried by passing through a column of the appropriate drying agent, then stored under argon in Teflon-valve ampoules over a potassium mirror or 3Å molecular sieves. NMR spectra were measured in benzene-d_6 (C_6D_6) or bromobenzene-d_5 (C_6D_5Br) solvents, which were pre-dried over CaH_2, distilled under reduced pressure and stored under argon in Teflon valve ampoules over 3 Å molecular sieves. NMR samples were prepared under argon in 5 mm Wilmad 507-PP tubes fitted with J. Young Teflon valves, and spectra measured on a Bruker Avance III HD nanobay 400 MHz NMR spectrometer, a Bruker Avance III 500 MHz NMR spectrometer, or a Bruker NEO 600 MHz NMR spectrometer equipped with a broadband helium cryoprobe. All ^{19}F, ^{31}P and ^{13}C NMR measurements were performed with proton decoupling. ^{1}H and ^{13}C NMR spectra were referenced internally to residual protio-solvent (^{1}H) or solvent (^{13}C) resonances which are reported relative to $SiMe_4$. ^{19}F, ^{27}Al, ^{29}Si, ^{31}P and ^{119}Sn NMR spectra were referenced externally with respect to $CFCl_3$, $[Al(H_2O)_6]^{3+}$, $SiMe_4$, H_3PO_4 and $SnMe_4$, respectively. ^{29}Si spectra have been corrected to remove the background glass signal. Chemical shifts are quoted in δ (ppm) and coupling constants in Hz. 2D NMR techniques (COSY, HSQC, and HMBC) were used to aid in assignment of spectra. Elemental analyses were carried out by London Metropolitan University. All reagents were commercially sourced and used as received unless stated otherwise.

2. Syntheses of Precursors

The compounds $Ar^{Mes}SnSi(SiMe_3)_3$ (**1**),[s1] $KSi(SiMe_3)_2Si^tBuPh_2$,[s2] $(Cy_3P)AuI$,[s3] and $Li[Al(OC(CF_3)_3)_4]$,[s4] were prepared according to existing procedures. Spectroscopic data matched those previously reported.

$Ar^{Mes}SnCl$: A procedure adapted from that originally reported by Power was used.[s5] A solution of $Ar^{Mes}Li$ (1.9 g, 5.9 mmol) in toluene (30 mL) was added to a suspension of $SnCl_2$ (1.1 g, 5.9 mmol) in toluene (30 mL) at room temperature with rapid stirring, which was continued for a further 2 h. The solution was diluted with hexane (10 mL) and filtered. Concentration of the solution to *ca*. one quarter of its original volume and storage overnight at −30 °C gave the product as yellow crystals. Yield 1.2 g, 43%. Spectroscopic data matched those reported by Power *et al.*[s5]

$Si(SiMe_3)_3(Si^tBuPh_2)$: A procedure adapted from that originally reported by Baumgartner was used.[s6] $Si(SiMe_3)_4$ (11 g, 33 mmol) and KO^tBu (3.9 g, 35 mmol) were combined in a Schlenk flask and THF (100 mL) added at room temperature with stirring, which was continued for 12 h. Volatiles were removed *in vacuo*, and the residue extracted into toluene (150 mL). To this solution was added dropwise a solution of Si^tBuPh_2Cl (9.5 g, 35 mmol) also in toluene (50 mL) at −78 °C. The reaction mixture was warmed to room temperature and stirred for 12 h, after which it was added to a mixture of ice, de-ionised water, sulphuric acid (0.5 M), and diethyl ether. The aqueous and organic layers were separated, and the aqueous layer extracted with diethyl ether (3 × 20 mL). The combined organic layers were then dried over magnesium sulphate, and volatiles removed in vacuo. Recrystallisation of the residue from acetone afforded the product as colourless blocks. Yield: 11 g, 67%. Spectroscopic data matched those reported by Baumgartner *et al.*[s6]

3. Syntheses of Novel Compounds

2: $Ar^{Mes}SnCl$ (0.78 g, 1.7 mmol) and $K\{Si(SiMe_3)_2(Si^tBuPh_2)\}$ (0.76 g, 1.7 mmol) were combined as solids and the mixture dissolved in toluene (20 mL), with immediate formation of a green solution. After stirring at room temperature for 2 h, volatiles were removed *in vacuo* to yield an oily green solid. Extraction into hexane (20 mL), filtration and concentration of the solution to *ca*. one quarter of its original volume and storage at room temperature overnight yielded **2** as a green solid, which was isolated by filtration. Blue crystals of suitable quality for X-ray crystallography were obtained by slow evaporation from a hexane solution at room temperature. Yield: 1.0 g, 70 %.

Calc. for $C_{46}H_{62}Si_4Sn$: C 65.30%, H 7.39%. Measured: C 65.20%, H 7.52%.

^1H NMR (400 MHz, C_6D_6, 298 K): δ_H 7.94 – 7.87 (m, 4H, CH Ph), 7.33 (t, $^3J_{HH}$ = 7.5 Hz, 1H, *p*-CH Ar^{Mes}), 7.22 – 7.17 (m, 4H, CH Ph), 7.16 – 7.13 (m, 2H, CH Ph), 7.04 (d, $^3J_{HH}$ = 7.5 Hz, 2H, m-CH Ar^{Mes}), 6.74 (s, 4H, *m*-CH Mes), 2.45 (s, 12H, *o*-CH_3 Mes), 2.10 (s, 6H, *p*-CH_3 Mes), 0.82 (s, 9H, $C(CH_3)_3$), 0.01 (s, 18H, $Si(Si(CH_3)_3)_2$) ppm.

$^{13}C\{^1H\}$ NMR (101 MHz, C_6D_6, 298 K): δ_C 182.7, 146.3, 139.0, 137.8, 137.8, 137.4, 136.2, 130.6, 130.2, 129.1, 128.5, 127.7, 29.4, 22.1, 21.2, 20.0, 5.1 ppm.

$^{29}Si\{^1H\}$ NMR (80 MHz, C_6D_6, 298 K): δ_{Si} 8.9, −3.2, −36.7 ppm.

$^{119}Sn\{^1H\}$ NMR (150 MHz, C_6D_6, 298 K): δ_{Sn} 2831 ppm.

3: To a stirred suspension of $K\{Si(SiMe_3)_2(Si^tBuPh_2)\}$ (2.1 g, 4.6 mmol) in pentane (50 mL) at −78 °C was added dropwise a solution of $Sn\{N(SiMe_3)_2\}_2$ (1.0 g, 2.3 mmol) also in pentane (50 mL). The reaction mixture was then stirred at −78 °C for 1 h to yield a green suspension, which was then warmed to 0 °C and filtered. Volatiles were removed *in vacuo* from the filtrate, and the residue extracted into pentane at -78 °C. After filtration, the filtrate was concentrated to incipient crystallisation and stored at −78 °C overnight to afford **3** as dark red crystals of suitable quality for X-ray crystallography. Yield: 0.42 g, 19%.

^1H NMR (500 MHz, C_6D_6, 298 K) δ_H: 7.89 – 7.84 (m, 8H, CH Ph), 7.25 – 7.12 (m, 12H, CH Ph), 1.04 (s, 18H, $C(CH_3)_3$), 0.38 (s, 36H, $Si(Si(CH_3)_3)_2$) ppm.

$^{13}C\{^1H\}$ NMR (126 MHz, C_6D_6, 298 K) δ_C: 138.8, 138.5, 136.9, 136.7, 129.5, 129.3, 128.4, 128.0, 127.5, 31.8, 29.7, 29.2, 21.0, 7.2, 5.4, 2.5 ppm.

$^{29}Si\{^1H\}$ NMR (99 MHz, C_6D_6, 298 K) δ_{Si}: 10.4, −3.3, −46.0 ppm.

$^{119}Sn\{^1H\}$ NMR (187 MHz, C_6D_6, 298 K) δ_{Sn}: not found.

4: **1** (69 mg, 0.10 mmol) and $(Ph_3P)AuCl$ (50 mg, 0.10 mmol) were combined as solids and the mixture dissolved in toluene (2 mL), with immediate formation of a pale-yellow solution. After stirring for 30 min,

volatiles were removed *in vacuo* to leave a brown oily solid. This solid was dissolved in hexane (5 mL), with rapid onset of crystallisation. Storage at room temperature for 1 h yielded **4** as a white powder, which was isolated by removal of the mother liquor *via* a thin cannula. Crystals of suitable quality for X-ray crystallography were obtained by slow evaporation from a hexane solution at room temperature. Yield: 48 mg, 40%.

Calc. for $C_{51}H_{67}AuClPSi_4Sn$: C 52.15%, H 5.75%. Measured: C 51.72%, H 5.81%.

^{1}H NMR (400 MHz, C_6D_6, 298 K) δ_H: 7.34 – 7.27 (m, 6H, CH Ph), 7.23 (t, $^{3}J_{HH}$ = 7.5 Hz, 1H, *p*-CH Ar^{Mes}), 7.03 – 6.94 (m, 13H, CH Ar), 6.55 (s, 2H, *m*-CH Mes), 2.43 (s, 6H, CH_3 Mes), 2.36 (s, 6H, CH_3 Mes), 2.16 (s, 6H, CH_3 Mes), 0.44 (s, 27H, $Si(Si(CH_3)_3)_3$) ppm.

$^{31}P\{^{1}H\}$ NMR (162 MHz, C_6D_6, 298 K) δ_P: 43.6 ($^{119/117}$Sn satellites: $^{2}J_{P\text{-}Sn}$ = 1890, 1800 Hz) ppm.

$^{13}C\{^{1}H\}$ NMR (101 MHz, C_6D_6, 298 K) δ_C: 152.0, 151.8, 150.7, 142.2, 137.5, 136.0, 135.9, 134.8, 134.6, 131.8, 131.4, 131.2, 131.2, 129.7, 129.2, 129.1, 129.0, 128.9, 23.9, 23.3, 21.5, 5.1 ppm.

$^{29}Si\{^{1}H\}$ NMR (80 MHz, C_6D_6, 298 K): δ_{Si} −6.0, −99.7 ppm (assigned by ^{29}Si/^{1}H HMBC).

$^{119}Sn\{^{1}H\}$ NMR (150 MHz, C_6D_6, 298 K) δ_{Sn}: 272 (d, $^{2}J_{P\text{-}Sn}$ = 1890 Hz) ppm.

The syntheses of **5**, **6** and **7** were performed in similar fashion:

5: **2** (88 mg, 0.10 mmol) and $(Ph_3P)AuCl$ (51 mg, 0.10 mmol) were combined as solids and the mixture dissolved in toluene (3 mL), with immediate formation of a pale-yellow solution. After stirring for 30 min, volatiles were removed *in vacuo* to leave a brown oily solid. This solid was dissolved in hexane (5 mL), with rapid onset of crystallisation. Storage at room temperature for 1 h yielded **5** as a white powder, which was isolated by removal of the mother liquor *via* a thin cannula. Crystals of suitable quality for X-ray crystallography were obtained by slow evaporation from a hexane solution at room temperature. Yield: 91 mg, 68%.

Calc. for $C_{64}H_{77}AuClPSi_4Sn$: C 57.33%, H 5.79%. Measured: C 57.89%, H 6.23%.

^{1}H NMR (400 MHz, C_6D_6, 298 K) δ_H: 7.88 – 7.83 (m, 4H, CH Ar), 7.44 – 7.36 (m, 6H, CH Ar), 7.19 – 7.10 (m, 7H, CH Ar), 7.04 – 6.97 (m, 9H, CH Ar), 6.94 – 6.88 (m, 4H, CH Ar), 6.61 (s, 2H, *m*-CH Mes), 2.48 (s, 6H, CH_3 Mes), 2.36 (s, 6H, CH_3 Mes), 2.19 (s, 6H, CH_3 Mes), 1.32 (s, 9H, $C(CH_3)_3$), 0.37 (s, 9H, $Si(CH_3)_3$), 0.05 (s, 9H, $Si(CH_3)_3$) ppm.

$^{31}P\{^{1}H\}$ NMR (162 MHz, C_6D_6, 298 K) δ_P: 45.2 ($^{119/117}$Sn satellites: $^{2}J_{P\text{-}Sn}$ = 1800, 1700 Hz) ppm.

$^{13}C\{^{1}H\}$ NMR (101 MHz, C_6D_6, 298 K) δ_C: 153.2, 153.0, 150.9, 142.3, 138.4, 138.3, 138.2, 137.3, 136.8, 136.2, 134.7, 134.6, 131.5, 131.3, 131.3, 131.1, 131.0, 129.4, 129.3, 129.2, 129.0, 129.0, 128.5, 127.7, 127.6, 30.6, 23.8, 23.5, 21.5, 20.9, 6.3, 5.1 ppm.

$^{29}Si\{^{1}H\}$ NMR (80 MHz, C_6D_6, 298 K): δ_{Si}: 6.5, -4.9, -7.2, -89.2 ppm (assigned by ^{29}Si/^{1}H HMBC).

$^{119}Sn\{^1H\}$ NMR (150 MHz, C_6D_6, 298 K): δ_{Sn}: 260 (d, $^2J_{Sn\text{-}P}$ = 1800 Hz) ppm.

6: **3** (50 mg, 0.053 mmol) and (Ph_3P)AuCl (26 mg 0.053 mmol) were combined as solids and the mixture dissolved in toluene (2 mL), with immediate formation of a brown solution. After stirring for 30 min, volatiles were removed *in vacuo* to leave an oily brown solid. This solid was dissolved in hexane (5 mL), with rapid onset of crystallisation. Storage at room temperature for 1 h **6** as a beige-coloured powder, which was isolated by removal of the mother liquor *via* a thin cannula. Brown crystals of suitable quality for X-ray crystallography were obtained by slow evaporation from a hexane solution at room temperature. Yield: 45 mg, 59%.

Calc. for $C_{62}H_{89}AuClPSi_8Sn{\cdot}C_7H_8$: C 50.17%, H 5.92%. Measured: C 49.98%, H 6.24%.

Due to the sparing solubility of this product in available deuterated solvents, the product was instead spectroscopically characterised *in-situ* by adding C_6D_6 (0.4 mL) to a J Young's NMR tube containing **3** (21 mg, 0.022 mol) and (Ph_3P)AuCl (11 mg, 0.022 mol).

^1H NMR (400 MHz, C_6D_6, 298 K) δ_H: 8.04 – 7.98 (m, 4H, CH Ph), 7.97 – 7.93 (m, 4H, CH Ph), 7.54 – 7.47 (m, 6H, CH Ph), 7.27 – 7.21 (m, 4H, CH Ph), 7.20 – 7.16 (m, 2H, CH Ph), 7.11 – 7.04 (m, 6H, CH Ph), 7.04 – 6.98 (m, 9H, CH Ph), 1.45 (s, 18H, $C(CH_3)_3$), 0.50 (s, 18H, $Si(CH_3)_3$), 0.49 (s, 18H, $Si(CH_3)_3$) ppm.

$^{31}P\{^1H\}$ NMR (162 MHz, C_6D_6, 298 K) δ_P: 46.6 ($^{119/117}Sn$ satellites: $^2J_{P\text{-}Sn}$ = 1390, 1330 Hz) ppm.

$^{13}C\{^1H\}$ NMR (101 MHz, C_6D_6, 298 K) δ_C: 138.4, 138.2, 137.8, 137.0, 134.7, 134.6, 134.4, 134.3, 131.5, 131.4, 131.3, 131.1, 129.3, 129.2, 129.1, 127.6, 31.1, 21.0, 5.7, 5.6 ppm.

$^{29}Si\{^1H\}$ NMR (80 MHz, C_6D_6, 298 K) δ_{Si}: 7.5, −4.3, −4.6, −89.9 ppm (assigned by $^{29}Si/^1H$ HMBC).

$^{119}Sn\{^1H\}$ NMR (149 MHz, C_6D_6, 298 K) δ_{Sn}: 281 (d, $^2J_{P\text{-}Sn}$ = 1400 Hz) ppm.

7: **1** (56 mg, 0.082 mmol) and (Cy_3P)AuI (50 mg, 0.083 mmol) were combined as solids and the mixture dissolved in toluene (2 mL), with immediate formation of a pale-yellow solution. After stirring for 30 min, volatiles were removed *in vacuo* to leave a brown oily solid. This solid was dissolved in hexane (2 mL), with rapid onset of crystallisation. Storage at room temperature for 1 h yielded **7** as a white powder, which was isolated via removal of the mother liquor *via* a thin cannula. Colourless crystals of suitable quality for X-ray crystallography were obtained by slow evaporation from a hexane solution at room temperature. Yield: 56 mg, 53 %.

^1H NMR (400 MHz, C_6D_6, 298 K): δ_H 7.21 (t, $^3J_{HH}$ = 7.5 Hz, 1H, *p*-CH Ar^{Mes}), 6.98 (d, $^3J_{HH}$ = 7.5 Hz, 2H, *m*-CH Ar^{Mes}), 6.97 (s, 2H, *m*-CH Mes), 6.94 (s, 2H, *m*-CH Mes), 2.48 (s, 6H, CH_3 Mes), 2.34 (s, 6H, CH_3 Mes), 2.30 (s, 6H, CH_3 Mes), 1.85 – 1.10 (m, 33H, $P(c\text{-}C_6H_{11})_3$), 0.47 (s, 27H, $Si(Si(CH_3)_3)_3$).

$^{31}P\{^1H\}$ NMR (162 MHz, C_6D_6, 298 K) δ_P: 62.7 ($^{119/117}Sn$ satellites: $^2J_{P\text{-}Sn}$ = 1880, 1800 Hz) ppm.

$^{13}C\{^1H\}$ NMR (101 MHz, C_6D_6, 298 K) δ_C: 150.9, 142.2, 137.5, 136.6, 136.1, 129.4, 129.3, 128.9, 128.8, 34.0, 33.8, 31.2, 31.2, 30.5, 27.6, 27.5, 26.2, 24.3, 23.7, 21.5, 5.3 ppm.

$^{29}Si\{^1H\}$ NMR (80 MHz, C_6D_6, 298 K): δ_{Si}: −5.3, −109.9 ppm (assigned by $^{29}Si/^1H$ HMBC).

$^{119}Sn\{^1H\}$ NMR (150 MHz, C_6D_6, 298 K): δ_{Sn}: 177 (d, $^2J_{Sn\text{-}P}$ = 1880 Hz) ppm.

8: To a J Young's NMR tube containing a solution of **7** (42 mg, 0.033 mmol) in *o*-DFB (1 mL) was added $Li[Al(OC(CF_3)_3)_4]$ (32 mg, 0.033 mmol). Sealing and inverting the tube several times resulted in the formation of a red solution and a colourless precipitate. After leaving to stand for 5 min, the reaction mixture was filtered through a syringe into a Schlenk flask. Addition of hexane (5 mL) and storage at -30 °C overnight produced a crop of product **8** as a red powder, which was isolated by removal of the mother liquor *via* a thin cannula. Crystals of suitable quality for X-ray crystallography were obtained from a concentrated solution in *o*-DFB overlaid with hexane. Yield: 30 mg, 47%.

^1H NMR (600 MHz, C_6D_5Br, 298 K) δ_H: 7.54 (t, $^3J_{HH}$ = 7.6 Hz, 1H, p-CH Ar^{Mes}), 7.14 (d, $^3J_{HH}$ = 7.6 Hz, 2H, *m*-CH Ar^{Mes}), 6.87 (s, 2H, *m*-CH Mes), 6.82 (s, 2H, *m*-CH Mes), 2.38 (s, 6H, CH_3 Mes), 2.21 (s, 6H, CH_3 Mes), 2.18 (s, 6H, CH_3 Mes), 2.09 – 2.02 (m, 3H, $P(c\text{-}C_6H_{11})_3$), 1.89 – 1.79 (m, 11H, $P(c\text{-}C_6H_{11})_3$), 1.75 – 1.70 (m, 3H, $P(c\text{-}C_6H_{11})_3$), 1.40 – 1.31 (m, 6H, $P(c\text{-}C_6H_{11})_3$), 1.31 – 1.23 (m, 7H, $P(c\text{-}C_6H_{11})_3$), 1.21 – 1.13 (m, 3H, $P(c\text{-}C_6H_{11})_3$), 0.13 (s, 27H, $Si(Si(CH_3)_3)_3$) ppm.

$^{19}F\{^1H\}$ NMR (565 MHz, C_6D_5Br, 298 K) δ_F: −74.6 ppm.

^{27}Al NMR (104 MHz, C_6D_5Br, 298 K) δ_{Al}: −35.4 ppm.

$^{31}P\{^1H\}$ NMR (162 MHz, C_6D_5Br, 298 K) δ_P: 71.8 ppm (separate $^{119/117}Sn$ satellites not resolved; $^2J_{Sn\text{-}P}$ = *ca*. 1360 Hz).

$^{13}C\{^1H\}$ NMR (101 MHz, C_6D_5Br, 298 K) δ_C: 159.5, 159.4, 145.9, 140.5, 137.2, 137.1, 135.0, 132.0, 131.3, 130.4, 121.4, 34.4, 34.3, 31.6, 27.7, 27.6, 26.4, 22.9, 22.4, 21.8, 4.3 ppm.

$^{29}Si\{^1H\}$ NMR (119 MHz, C_6D_5Br, 298 K) δ_{Si}: −3.3, −60.1, −60.2 ppm.

$^{119}Sn\{^1H\}$ NMR (224 MHz, C_6D_5Br, 298 K) δ_{Sn}: 1816 (d, $^2J_{Sn\text{-}P}$ = 1390 Hz) ppm.

4. X-ray Crystallographic Details

Single-crystal X-ray diffraction data were measured on an Oxford Diffraction/Agilent SuperNova diffractometer equipped with a 135 mm Atlas CCD area detector. Crystals were picked under Pantone-N oil and mounted on MiTeGen Micromount loops whilst being quench-cooled using an Oxford Cryosystems open flow N_2 cooling device.[s7] All data were collected at 150 K using mirror monochromated Cu Kα radiation (λ = 1.54184 Å) or Mo Kα radiation (λ = 0.70173 Å). Data were processed using the CrysAlisPro package, and structures were solved *ab initio* from the integrated intensities using SHELXT and refined on F^2 using SHELXL with the graphical interface OLEX2.[s8–s10]

Table s1. X-ray crystallographic details for compounds **2** – **8**.

	2	3	4	5	6	7	8
**Empirical Formula**	$C_{46}H_{62}Si_4Sn$	$C_{44}H_{74}Si_8Sn$	$C_{51}H_{67}AuClPSi_4Sn$	$C_{64}H_{77}AuClPSi_4Sn$	$C_{62}H_{89}AuClPSi_8Sn$	$C_{54}H_{92}AuIPSi_4Sn$	$C_{67}H_{85}AlAuF_{36}O_4PSi_4Sn$
**Formula weight (g mol^{-1})**	846.00	946.44	1174.48	1340.69	1441.12	1327.16	2124.31
**Temperature (K)**	150.00(10)	150.00(10)	149.9(3)	150.01(10)	150.00(10)	150.00(10)	150.00(10)
**Crystal system**	orthorhombic	orthorhombic	triclinic	triclinic	monoclinic	monoclinic	monoclinic
**Space group**	Aea2	$P2_12_12_1$	P-1	P-1	$P2_1/n$	C2/c	I2/a
**a (Å)**	31.7921(2)	10.2634(3)	10.31030(10)	11.5167(4)	14.6158(2)	42.7722(3)	30.8090(9)
**b (Å)**	19.10910(10)	17.5762(5)	14.4590(2)	15.9573(7)	25.0888(3)	13.42440(10)	11.8446(3)
**c (Å)**	14.97990(10)	28.7554(8)	17.8863(3)	17.0352(9)	18.4933(3)	21.4975(2)	46.6552(10)
**α (°)**	90	90	88.3040(10)	88.839(4)	90	90	90
**ß (°)**	90	90	87.8350(10)	84.705(4)	101.801(2)	102.1170(10)	92.091(2)
**λ (°)**	90	90	88.2710(10)	82.883(3)	90	90	90
**V (Å^{-3})**	9100.57(10)	5187.2(3)	2662.23(6)	3093.2(2)	6638.03(17)	12068.67(17)	17014.1(8)
**Z**	8	4	2	2	4	8	8
**ρ_{calc} (g cm^{-3})**	1.235	1.212	1.465	1.439	1.442	1.461	1.659
**μ (mm^{-1})**	5.684	0.705	10.677	9.266	9.341	13.050	7.462
**F(000)**	3552.0	2000.0	1180.0	1356.0	2936.0	5352.0	8448.0
**Crystal size (mm^{-3})**	0.2 × 0.2 × 0.2	0.3 × 0.2 × 0.2	0.4 × 0.1 × 0.1	0.2 × 0.05 × 0.05	0.2 × 0.2 × 0.2	0.4 × 0.3 × 0.1	0.2 × 0.1 × 0.1
**Radiation, λ (Å)**	1.54184	0.71073	1.54184	1.54184	1.54184	1.54184	1.54184
**2Θ range for data colln (°)**	7.998 to 152.286	6.73 to 61.29	7.76 to 152.488	7.604 to 152.736	7.044 to 136.5	6.916 to 152.122	7.584 to 140.152
**Index ranges**	-39 ≤ h ≤ 39, -23 ≤ k ≤ 24, -15 ≤ l ≤ 18	-14 ≤ h ≤ 10, -24 ≤ k ≤ 23, -40 ≤ l ≤ 35	-11 ≤ h ≤ 12, -18 ≤ k ≤ 18, -22 ≤ l ≤ 22	-14 ≤ h ≤ 14, -17 ≤ k ≤ 20, -21 ≤ l ≤ 21	-17 ≤ h ≤ 17, -30 ≤ k ≤ 28, -22 ≤ l ≤ 22	-53 ≤ h ≤ 53, -16 ≤ k ≤ 16, -21 ≤ l ≤ 27	-37 ≤ h ≤ 33, -14 ≤ k ≤ 14, -56 ≤ l ≤ 56
**Reflections collected**	47601	57458	44616	34381	47574	72997	81175
**Independent reflections**	8449	14497	11014	12765	12175	12532	16173
**Data/restraints/parameters**	8449 / 1 / 476	14497 / 0 / 496	11014 / 0 / 547	12765 / 0 / 664	12175 / 0 / 685	12532 / 0 / 575	16173 / 4094 / 1683
**Goodness-of-fit on F^2**	1.047	1.059	1.039	1.023	1.049	1.059	1.210
**Final R indexes [I ≥ 2σ (I)]**	R_1 = 0.0252 ωR_2 = 0.0559	R_1 = 0.0327 ωR_2 = 0.0529	R_1 = 0.0178 ωR_2 = 0.0440	R_1 = 0.0498 ωR_2 = 0.1230	R_1 = 0.0386 ωR_2 = 0.1056	R_1 = 0.0413 ωR_2 = 0.1072	R_1 = 0.1129 ωR_2 = 0.2376
**Final R indexes [all data]**	R_1 = 0.0281 ωR_2 = 0.0581	R_1 = 0.0459 ωR_2 = 0.0578	R_1 = 0.0193 ωR_2 = 0.0448	R_1 = 0.0772 ωR_2 = 0.1397	R_1 = 0.0488 ωR_2 = 0.1157	R_1 = 0.0438 ωR_2 = 0.1111	R_1 = 0.1322 ωR_2 = 0.2474
**Largest diff. peak/hole (e Å^{-3})**	0.30 / -0.36	0.42 / -0.28	1.36 / -0.69	2.38 / -2.31	2.15 / -0.84	1.33 / -1.81	2.38 / -2.03
**CCDC ref**	2518159	2518164	2518161	2518158	2518162	2518163	2518160

5. NMR Spectra of Novel Compounds

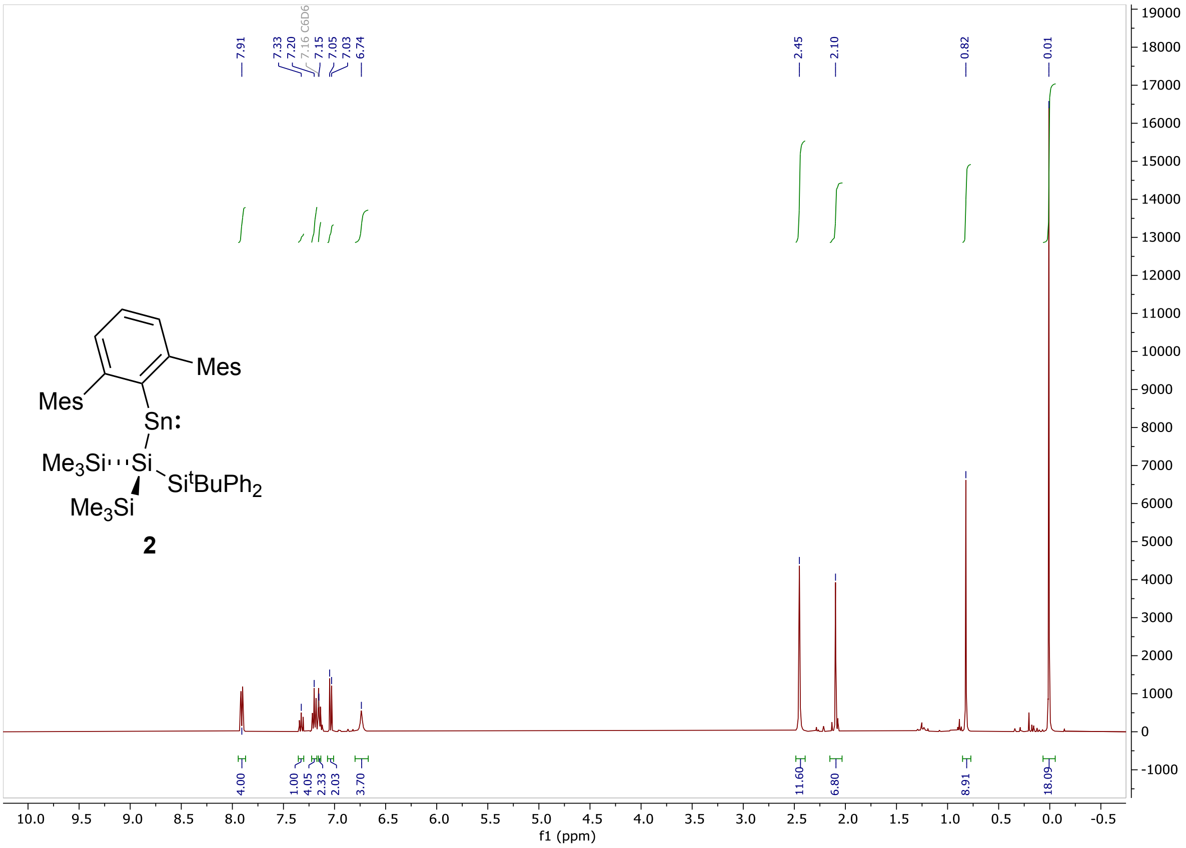

Figure S1. ^{1}H NMR spectrum of **2** in C_6D_6 at 298 K.

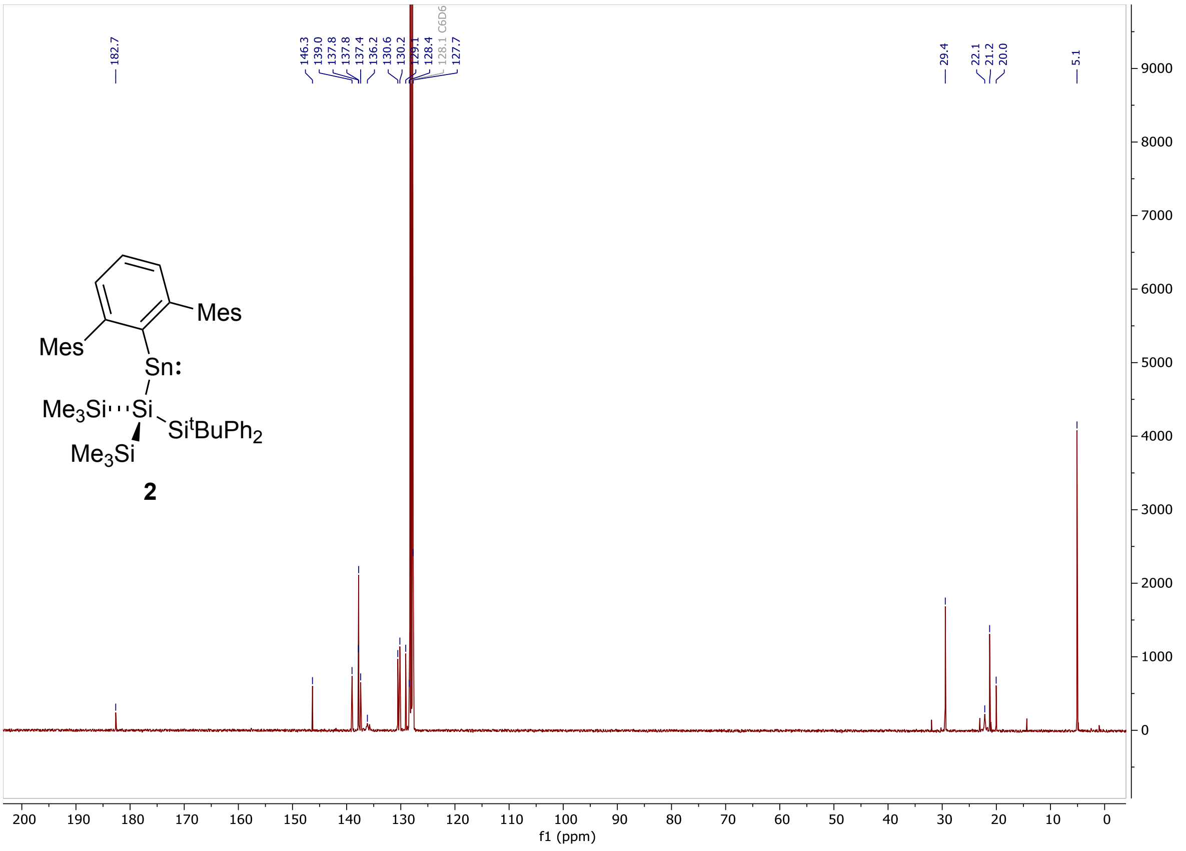

Figure S2. ^{13}C NMR spectrum of **2** in C_6D_6 at 298 K.

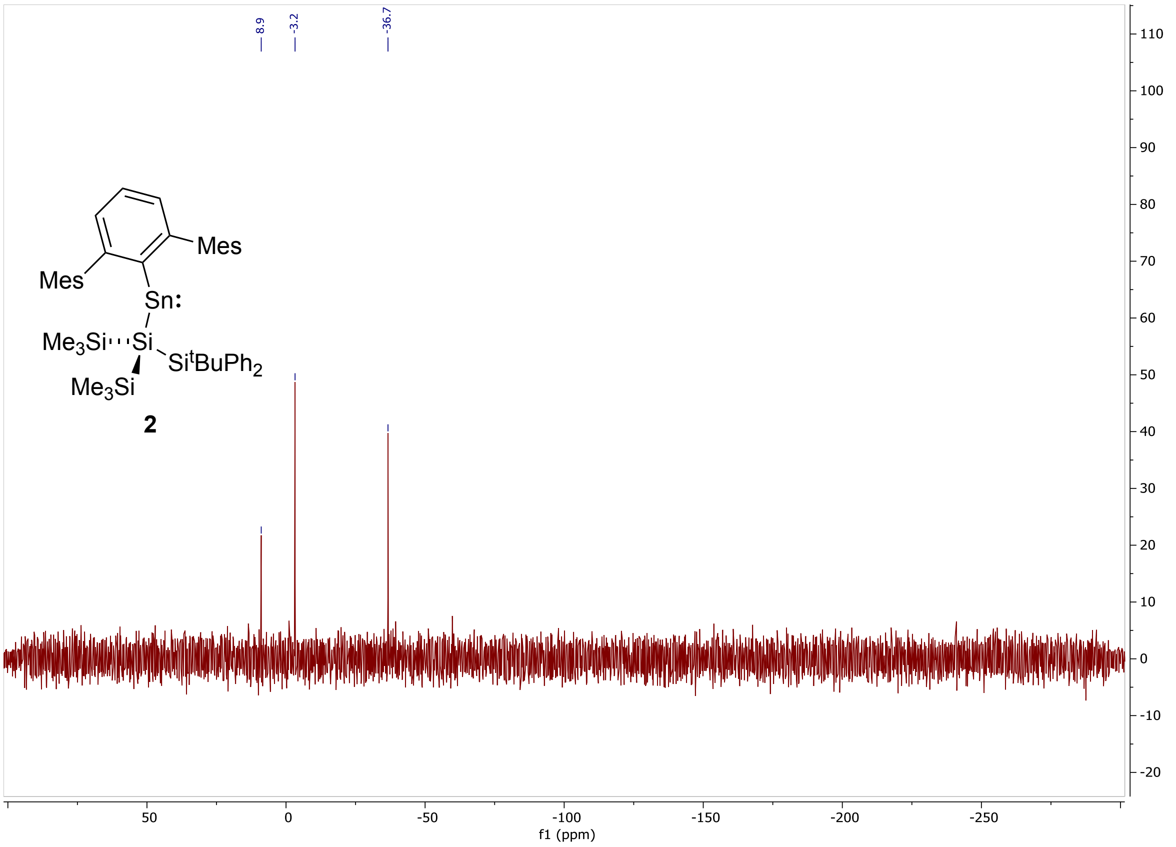

Figure S3. ^{29}Si NMR spectrum of **2** in C_6D_6 at 298 K.

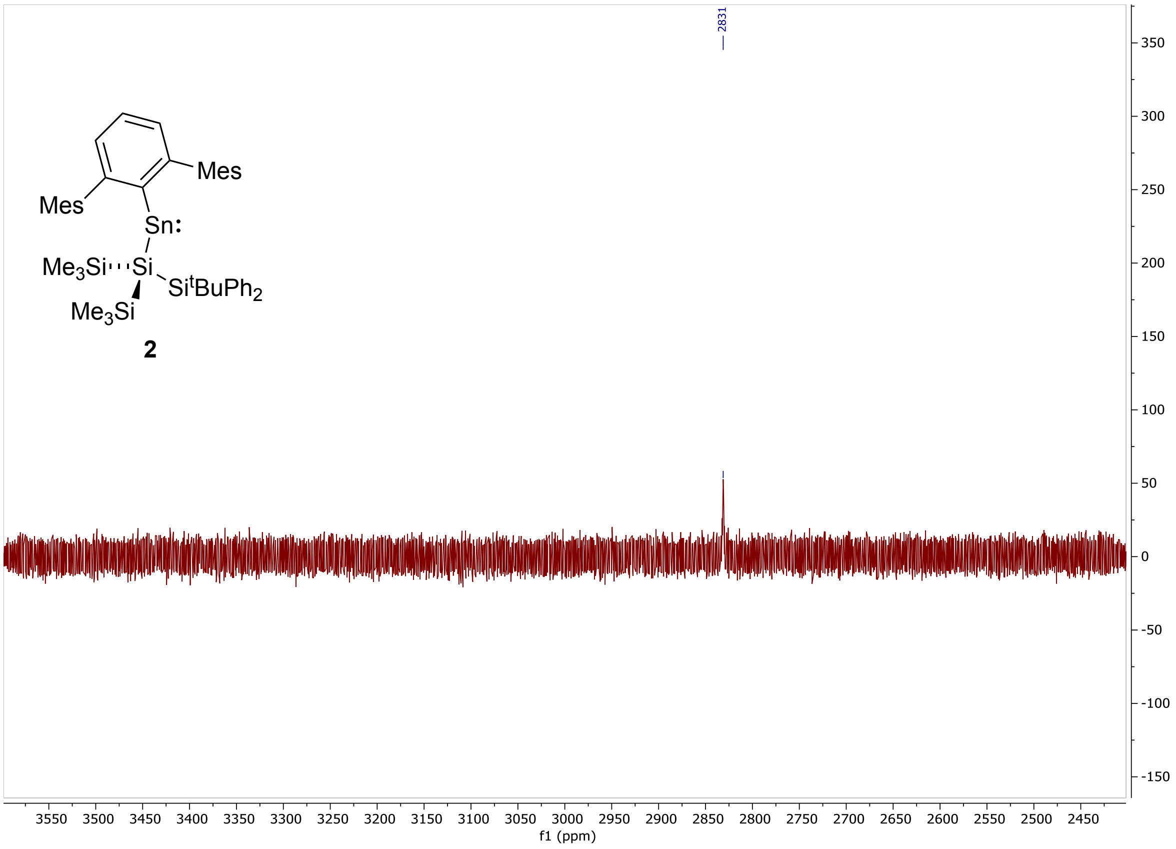

Figure S4. ^{119}Sn NMR spectrum of **2** in C_6D_6 at 298 K.

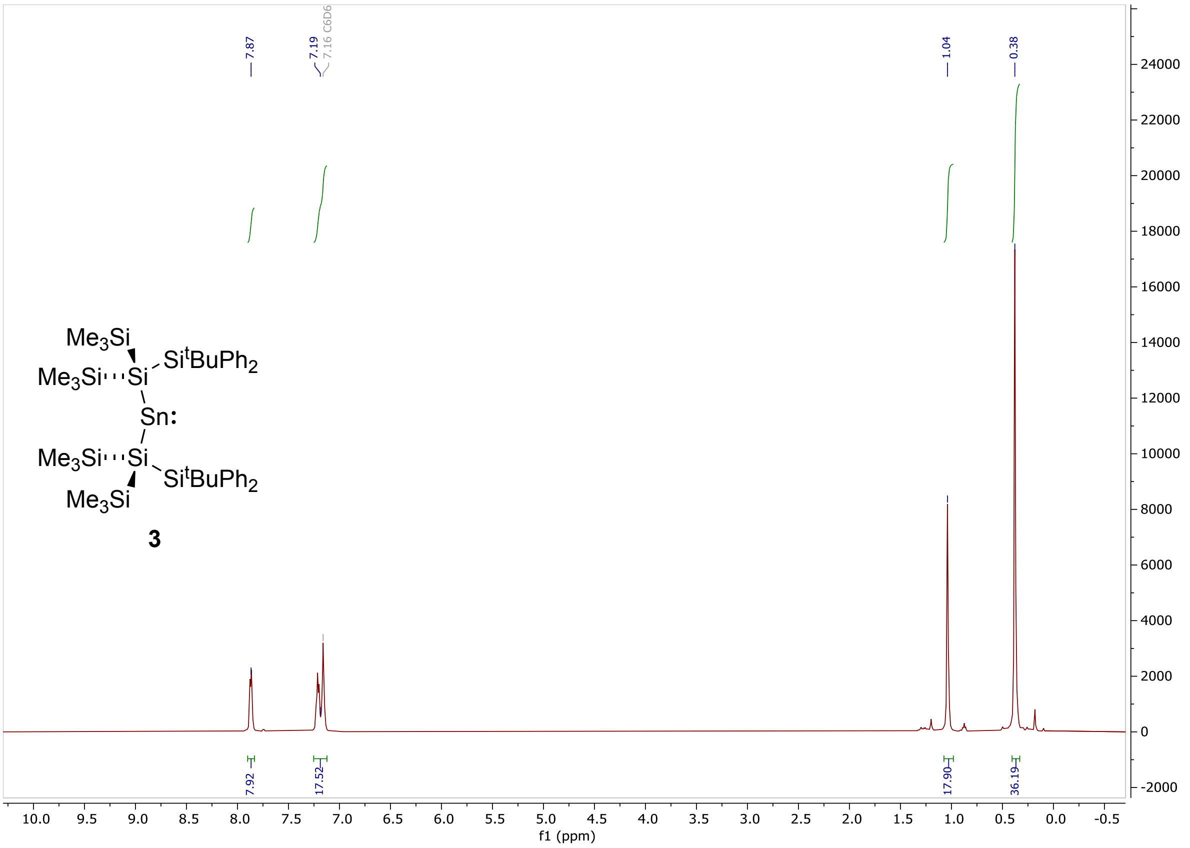

Figure S5. ^{1}H NMR spectrum of **3** in C_6D_6 at 298 K.

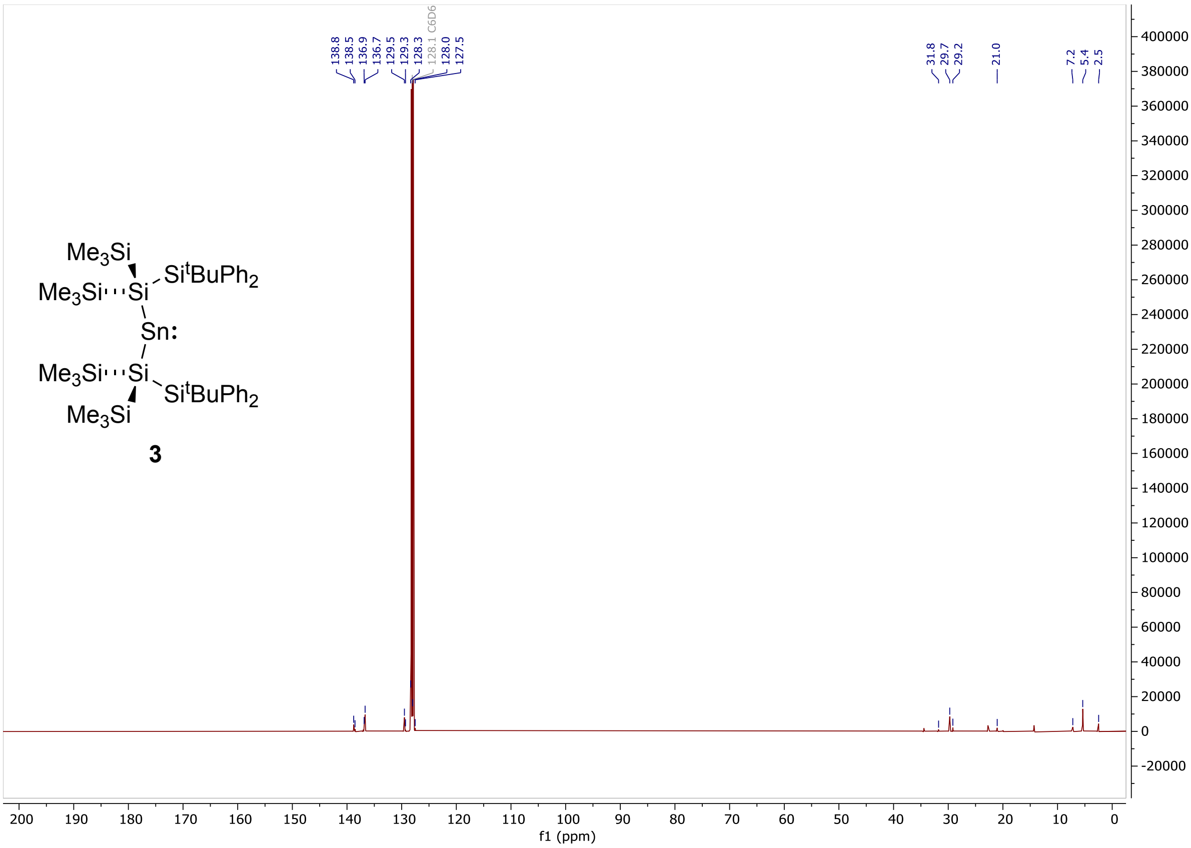

Figure S6. ^{13}C NMR spectrum of **3** in C_6D_6 at 298 K.

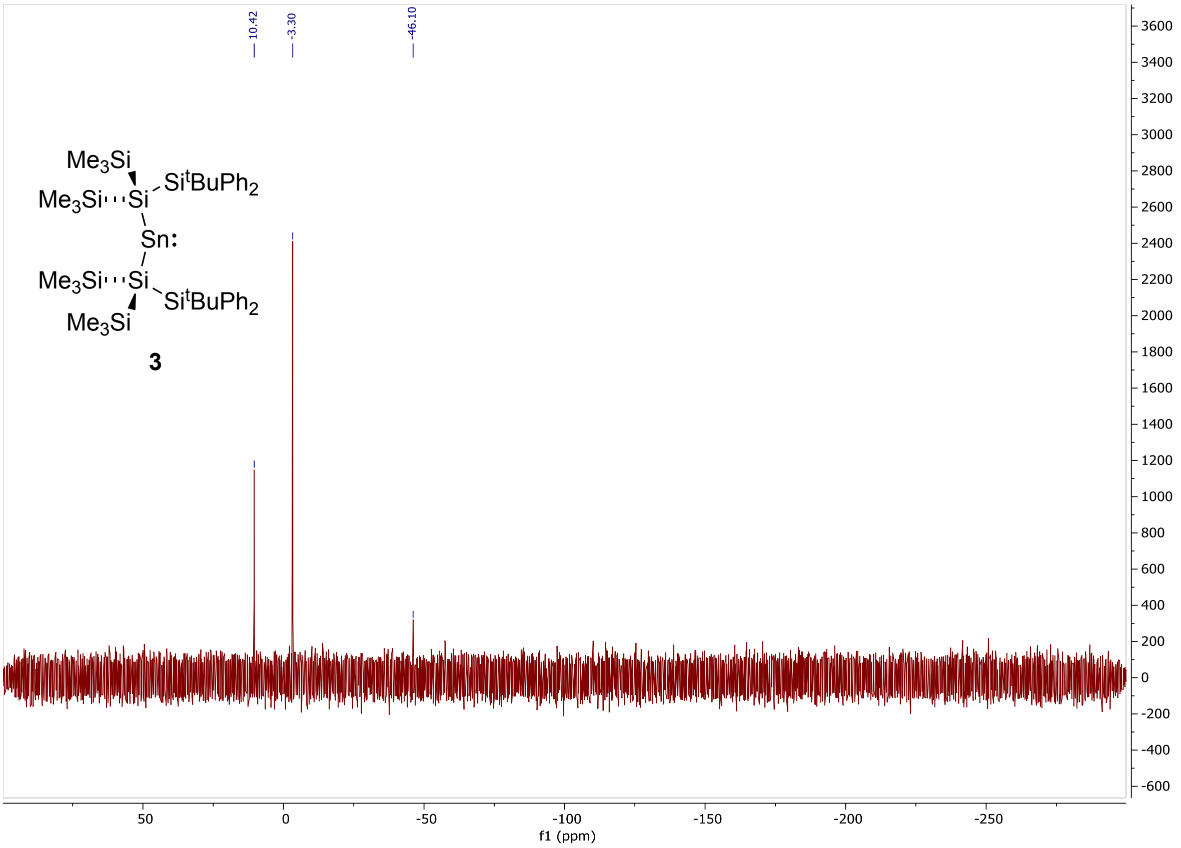

Figure S7. ^{29}Si NMR spectrum of **3** in C_6D_6 at 298 K.

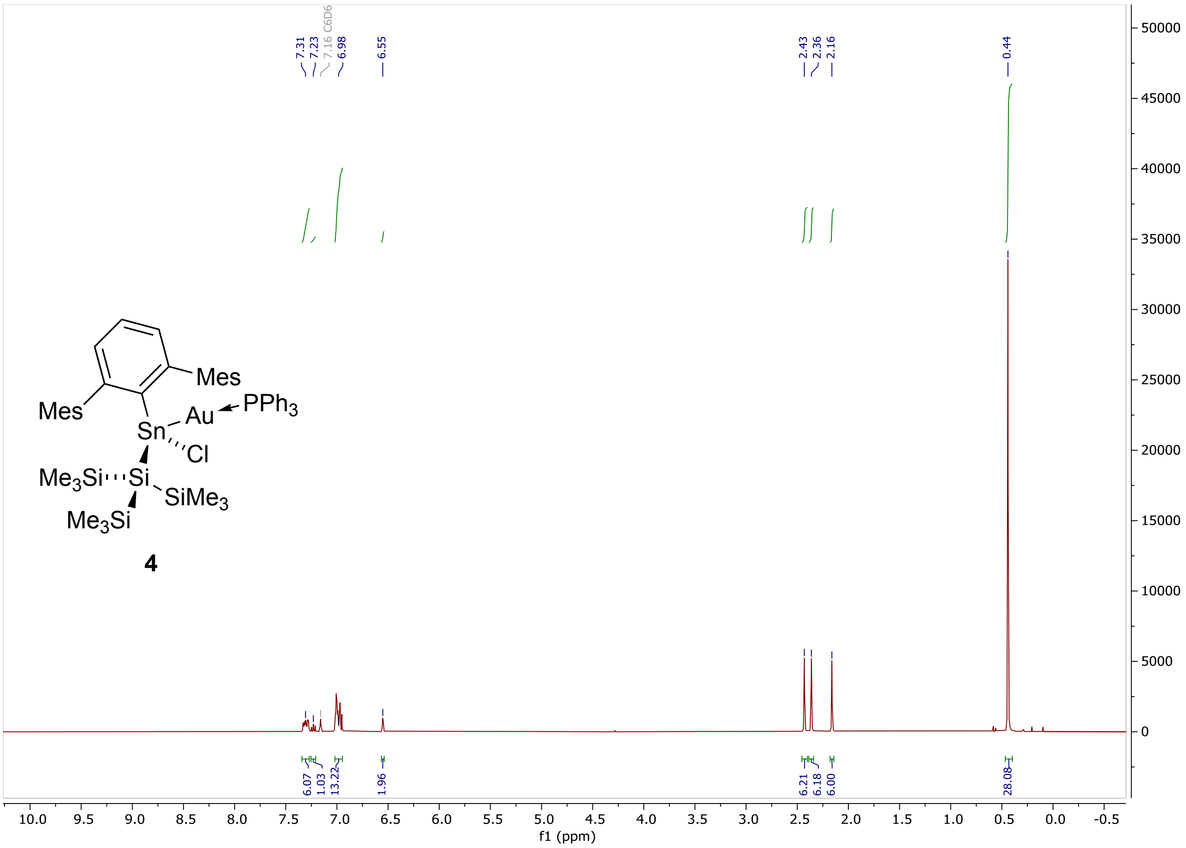

Figure S8. ^{1}H NMR spectrum of **4** in C_6D_6 at 298 K.

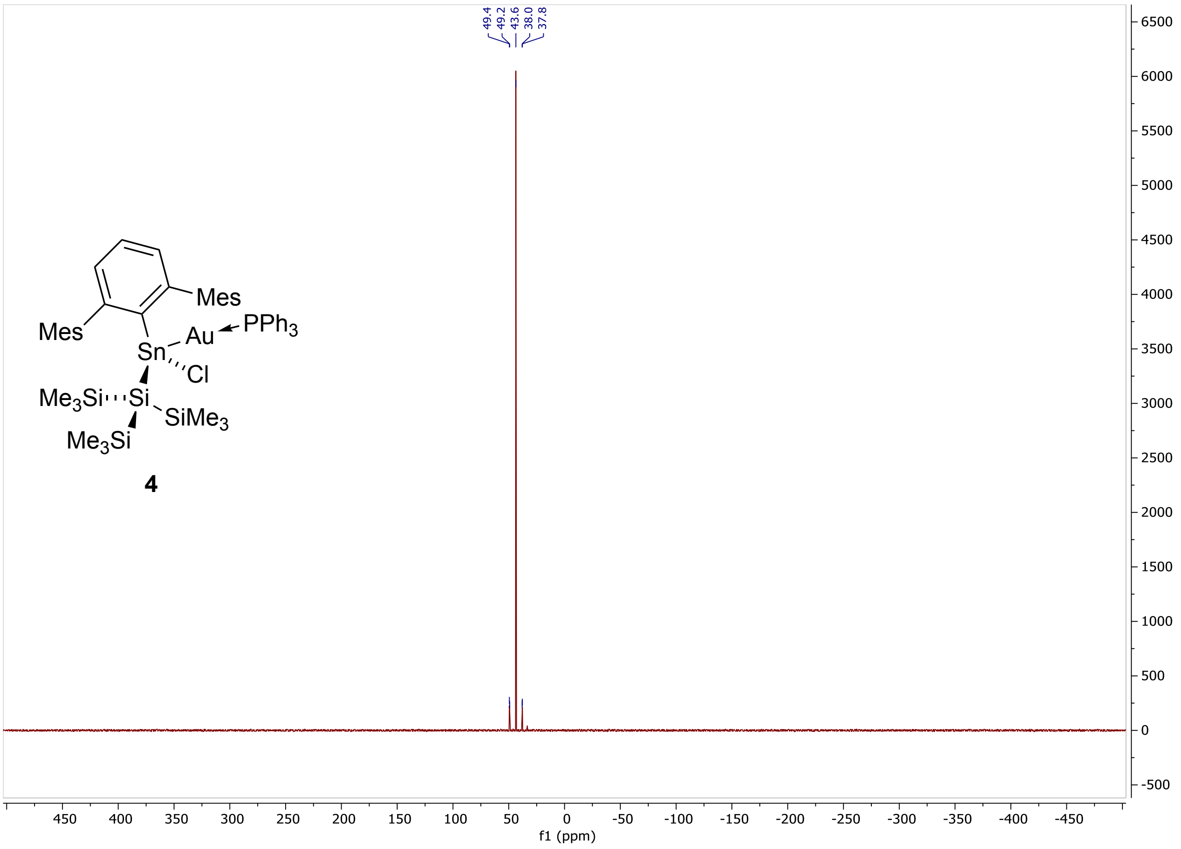

Figure S9. ^{31}P NMR spectrum of **4** in C_6D_6 at 298 K.

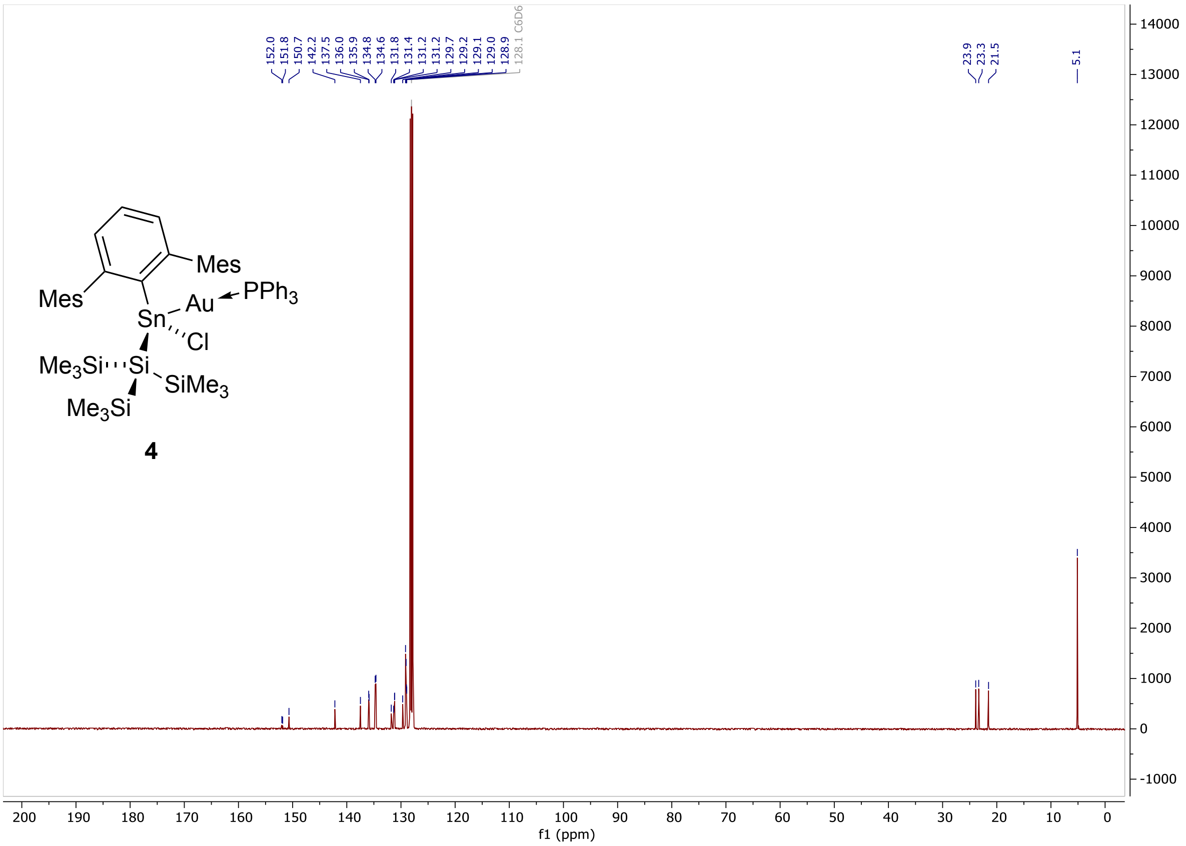

Figure S10. ^{13}C NMR spectrum of **4** in C_6D_6 at 298 K.

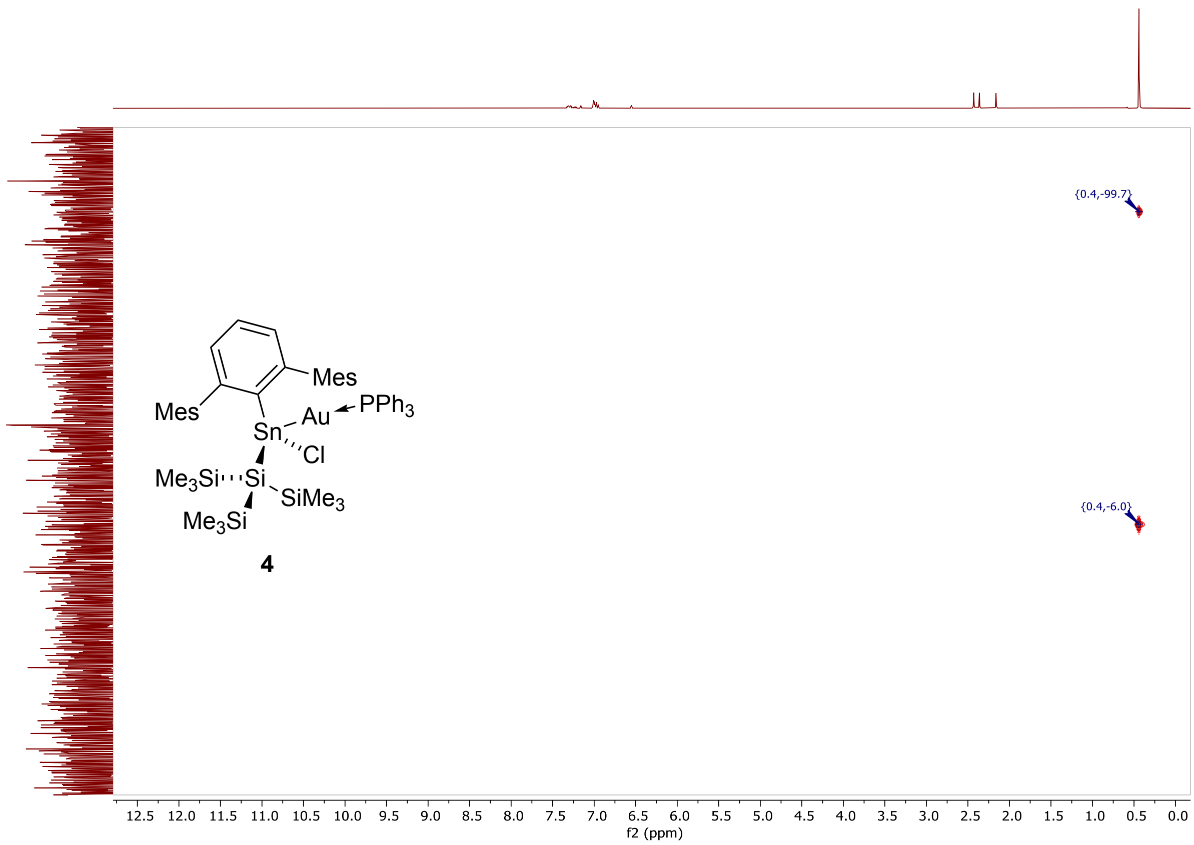

Figure S11. $^{29}Si/^{1}H$ HMBC spectrum of **4** in C_6D_6 at 298 K.

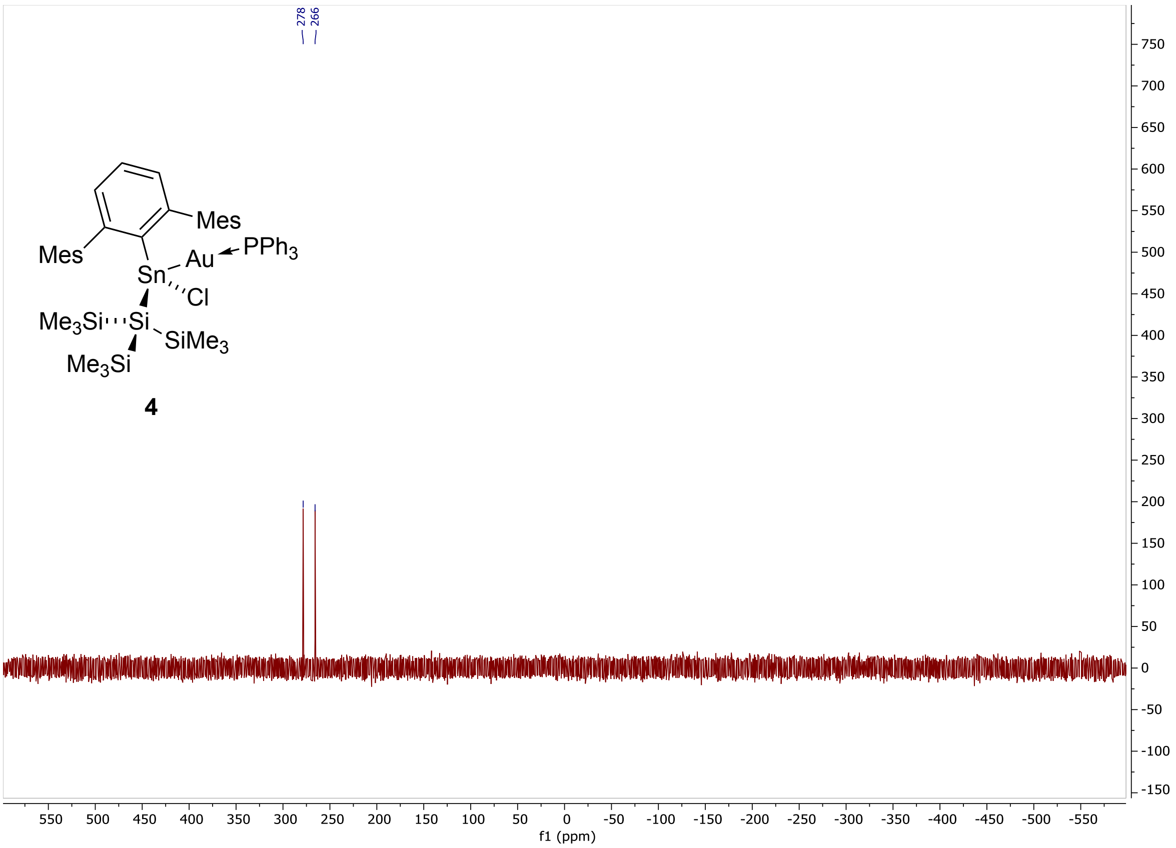

Figure S12. ^{119}Sn NMR spectrum of **4** in C_6D_6 at 298 K.

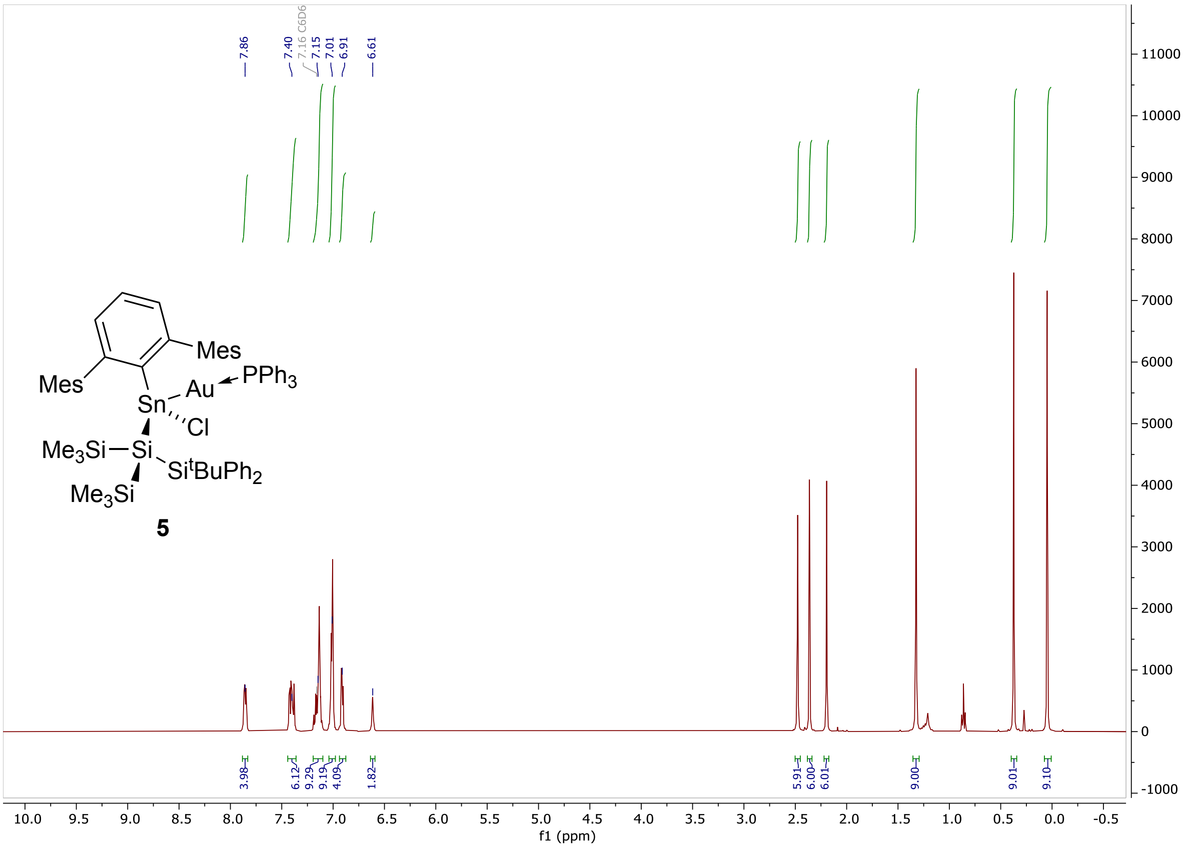

Figure S13. ^{1}H NMR spectrum of **5** in C_6D_6 at 298 K.

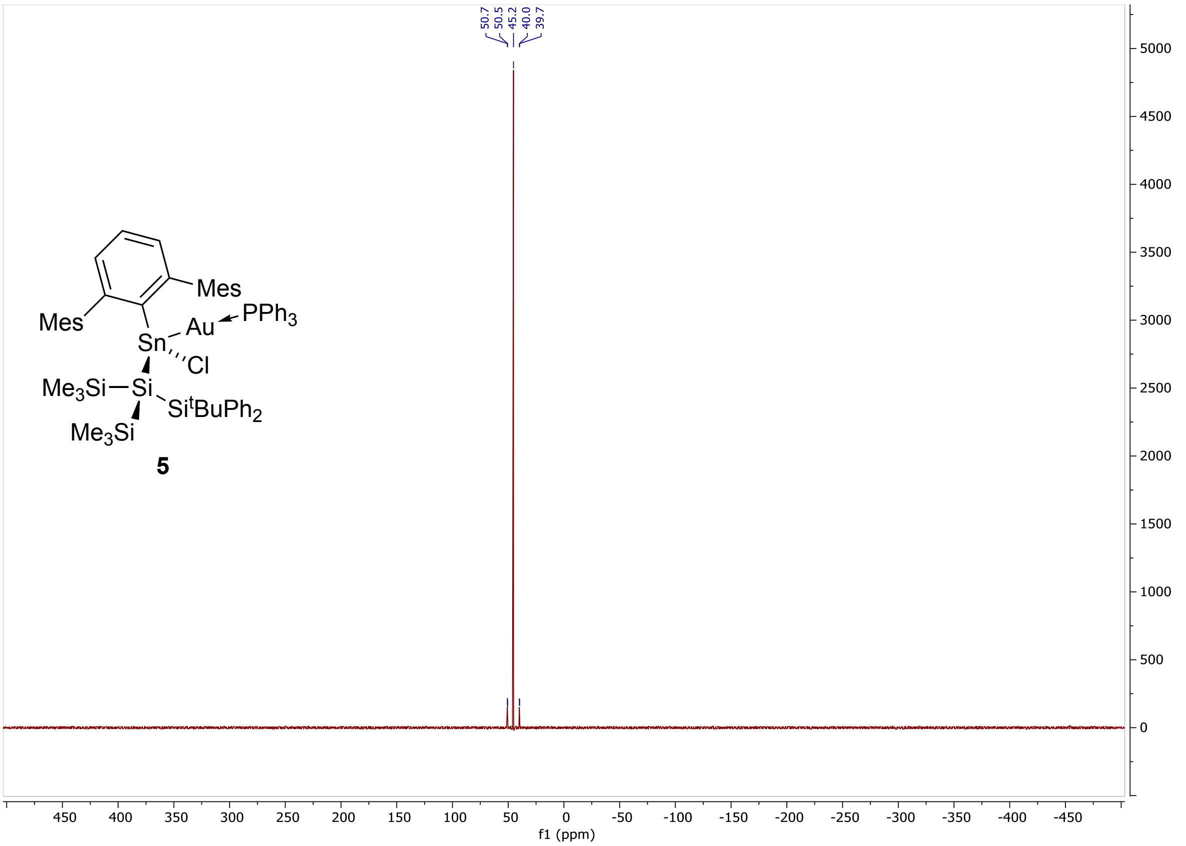

Figure S14. ^{31}P NMR spectrum of **5** in C_6D_6 at 298 K.

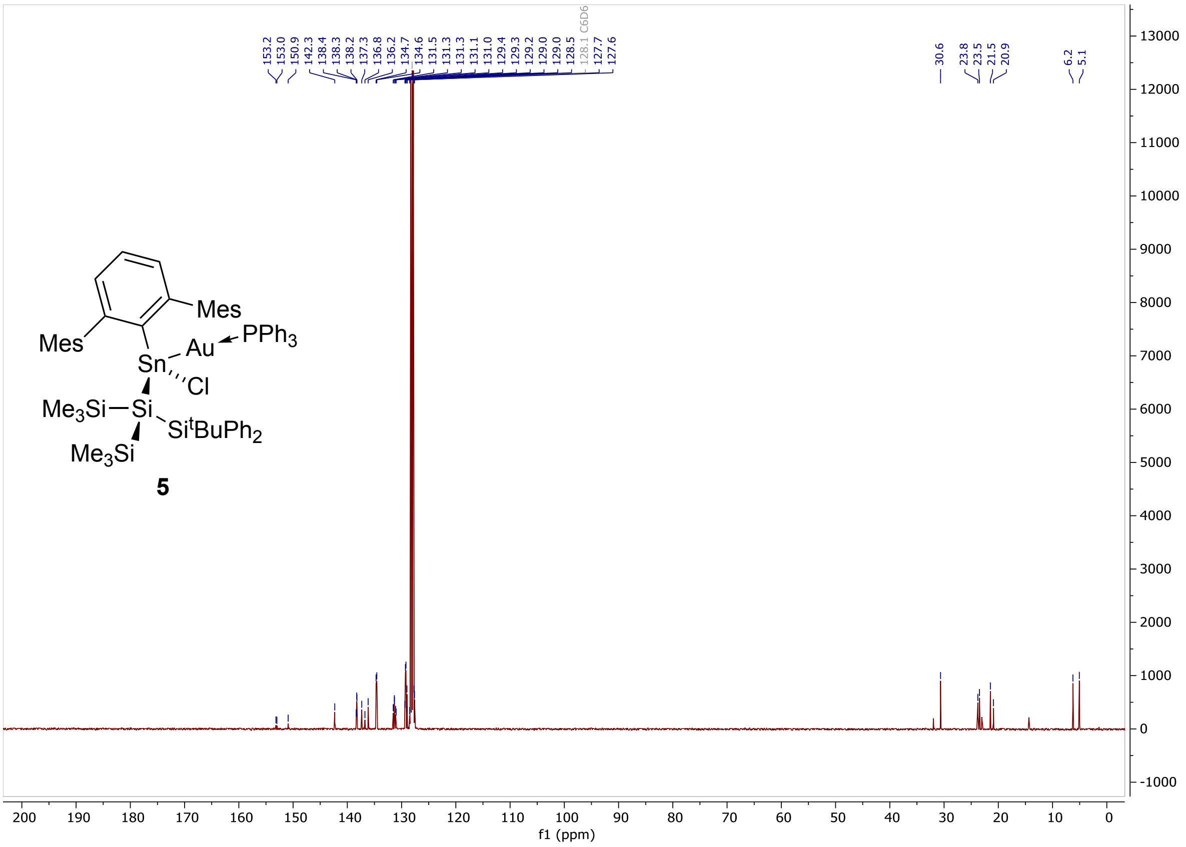

Figure S15. ^{13}C NMR spectrum of **5** in C_6D_6 at 298 K.

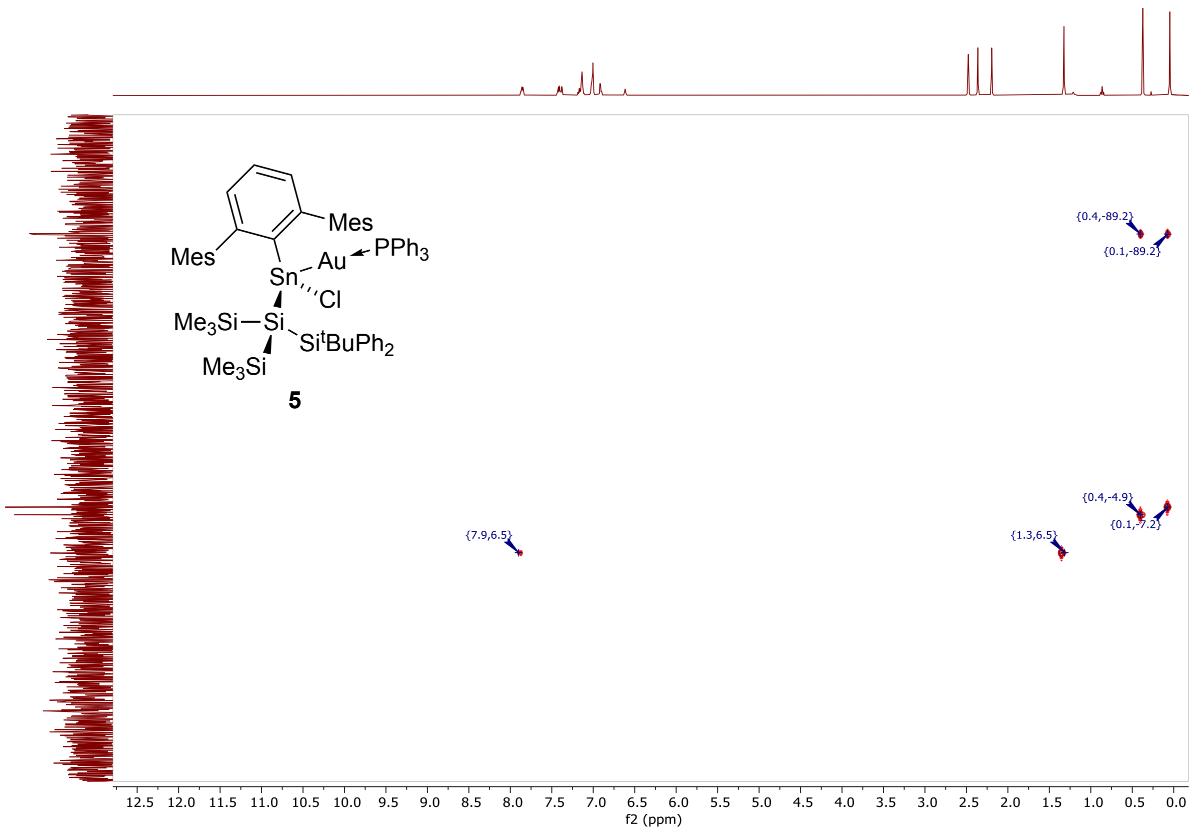

Figure S16. $^{29}Si/^{1}H$ HMBC spectrum of **5** in C_6D_6 at 298 K.

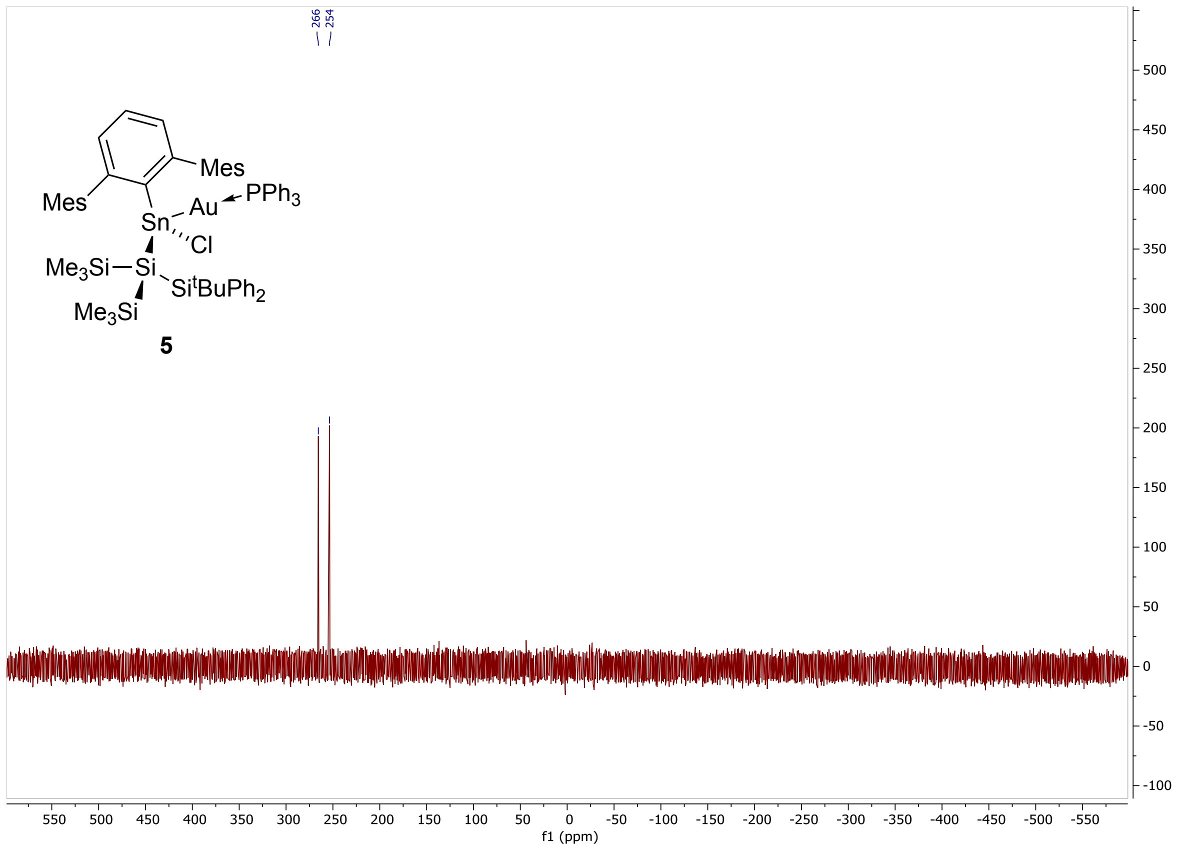

Figure S17. ^{119}Sn NMR spectrum of **5** in C_6D_6 at 298 K.

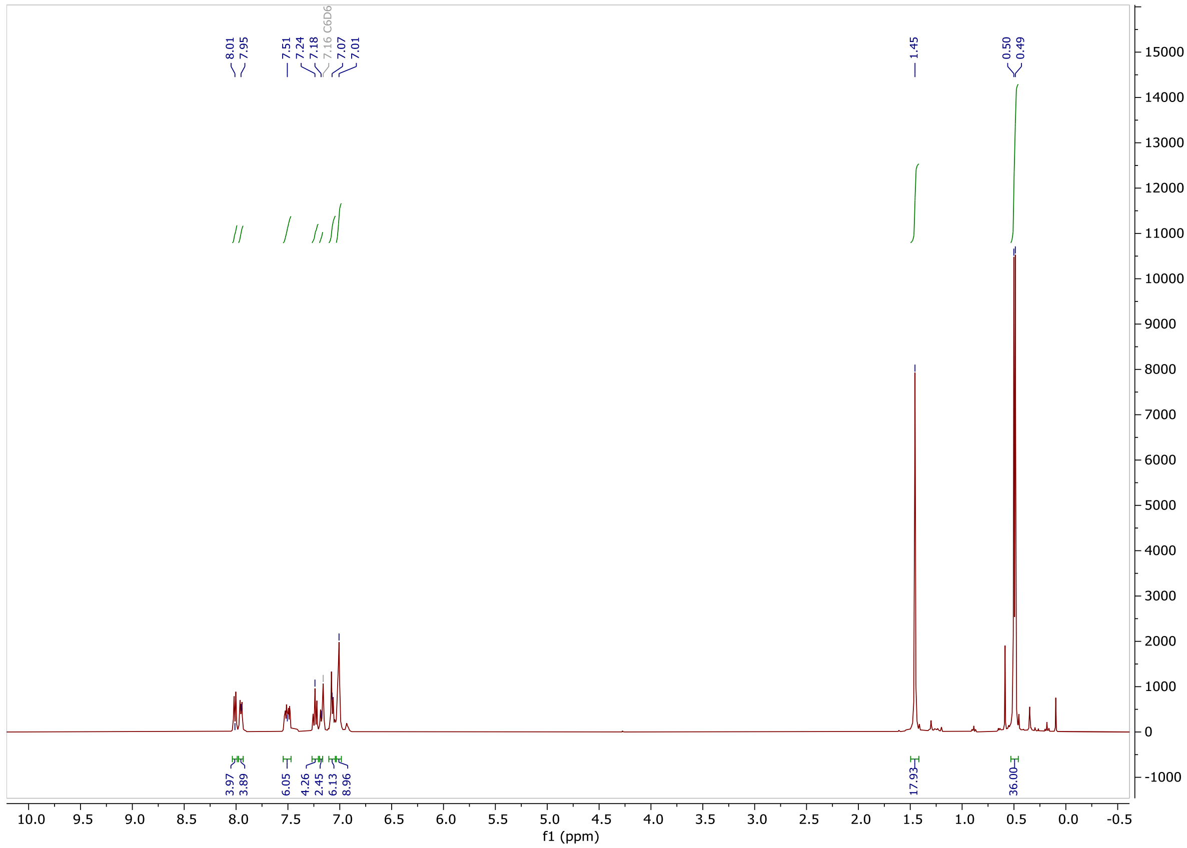

Figure S18. ^{1}H NMR spectrum of **6** in C_6D_6 at 298 K.

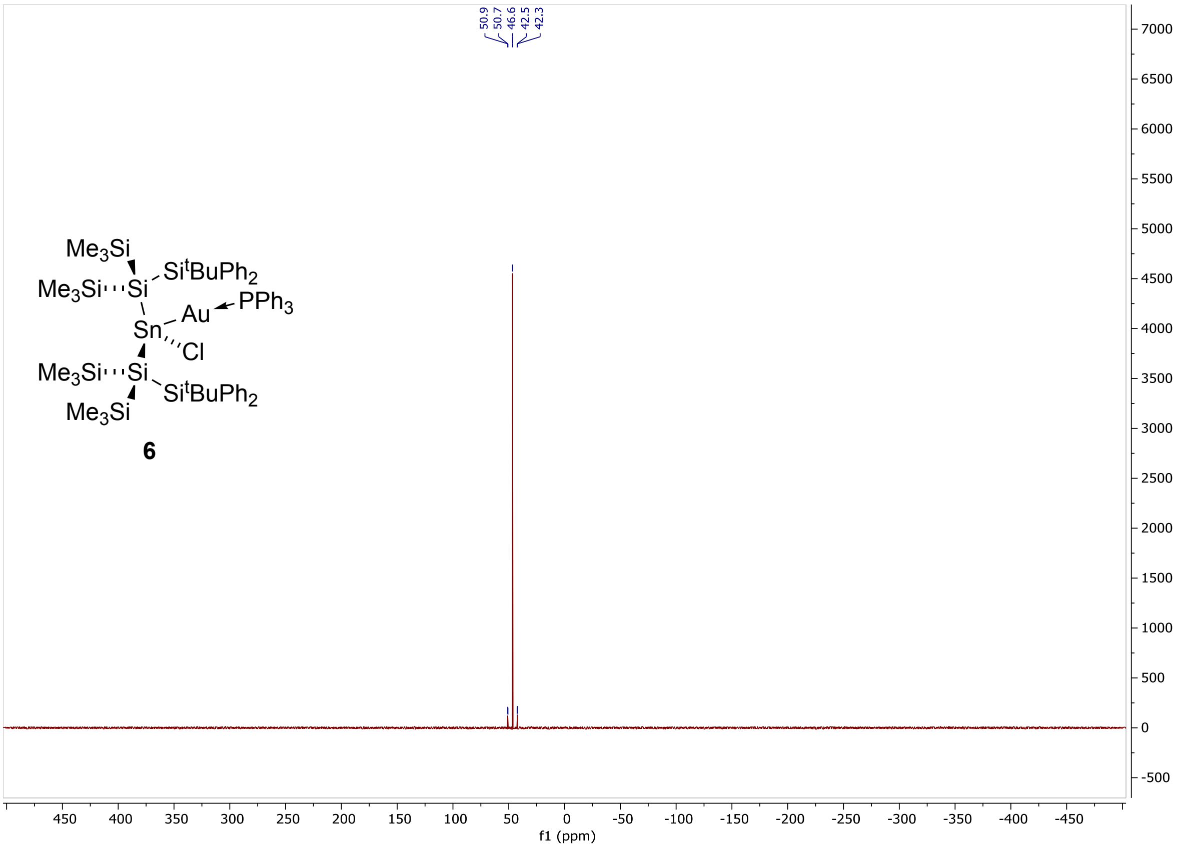

Figure S19. ^{31}P NMR spectrum of **6** in C_6D_6 at 298 K.

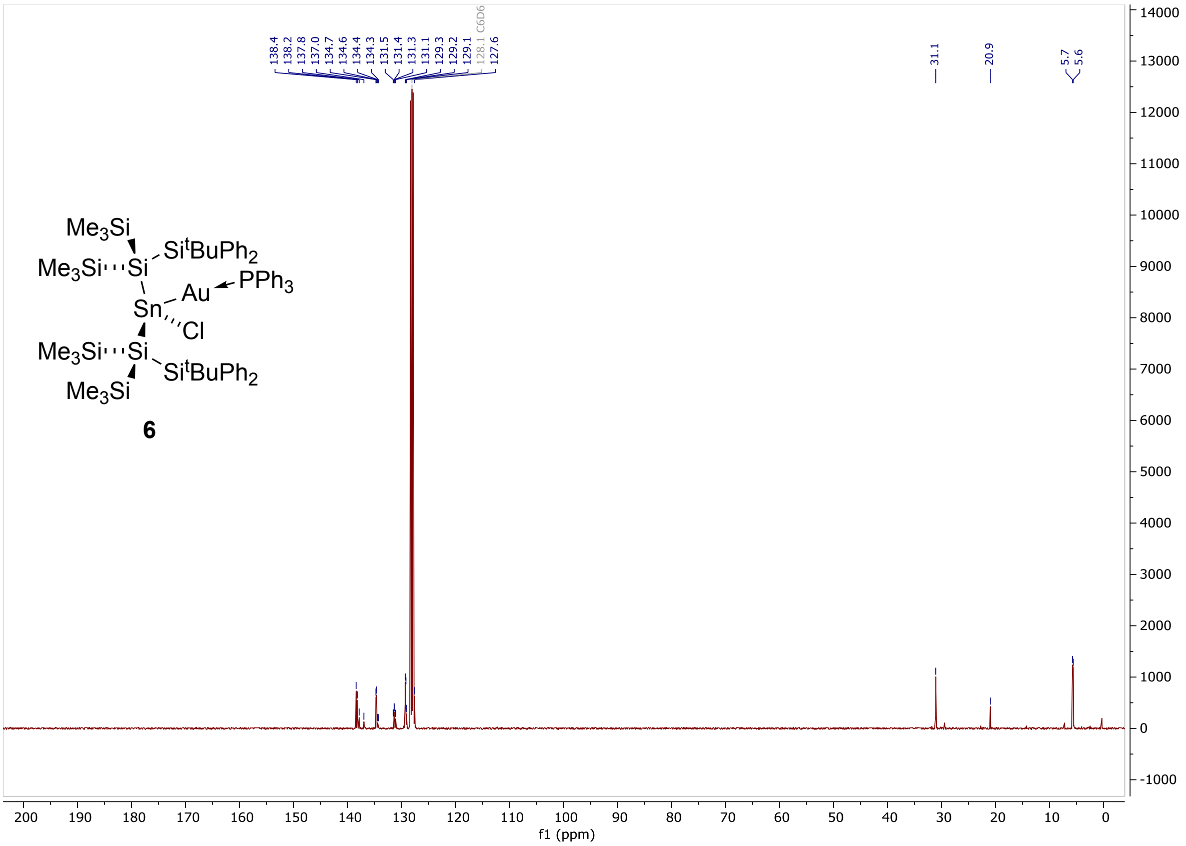

Figure S20. ^{13}C NMR spectrum of **6** in C_6D_6 at 298 K.

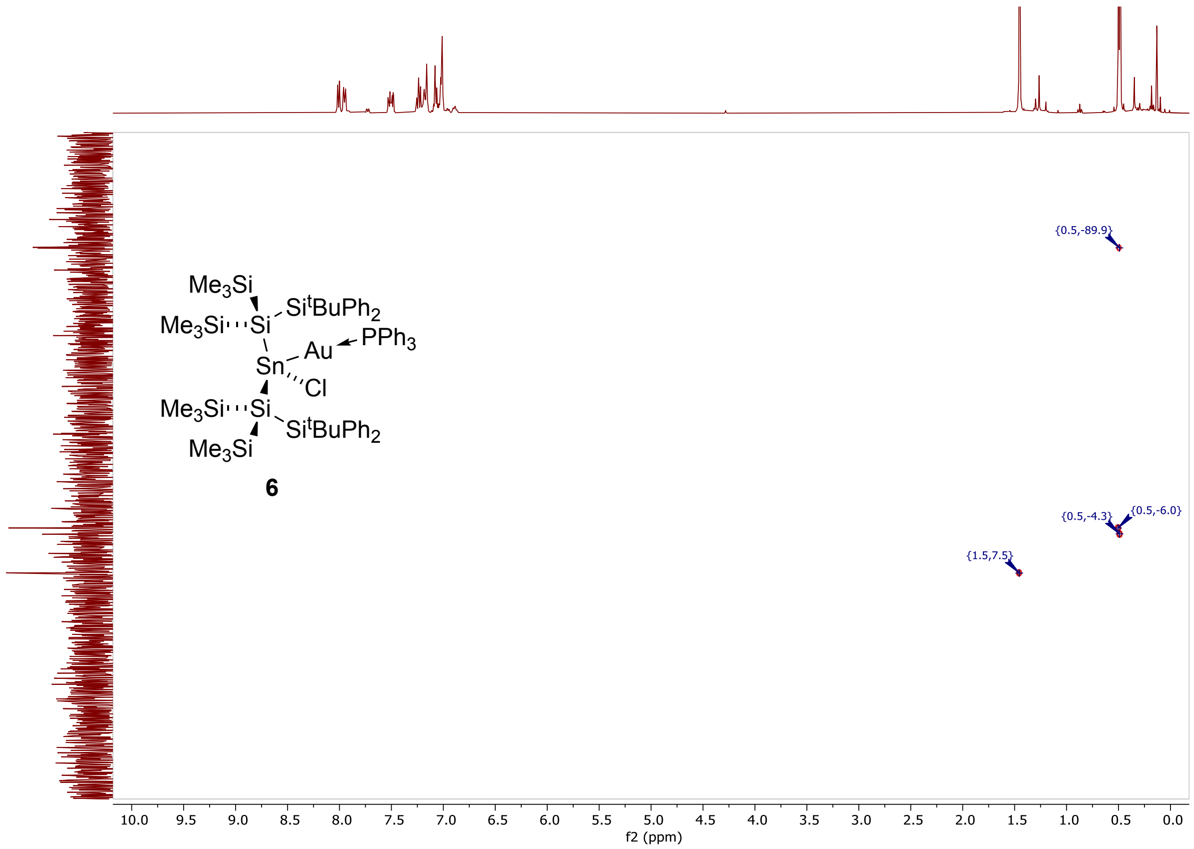

Figure S21. $^{29}Si/^{1}H$ HMBC spectrum of **6** in C_6D_6 at 298 K.

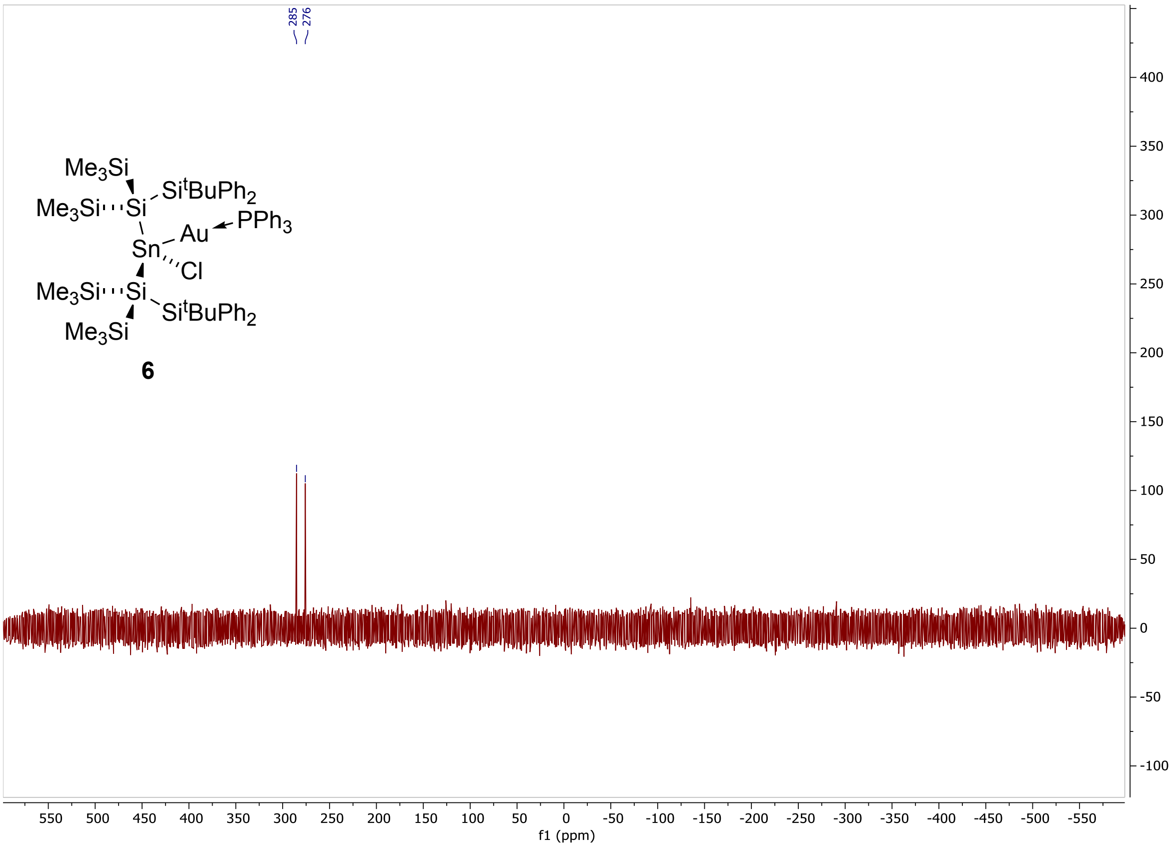

Figure S22. ^{119}Sn NMR spectrum of **6** in C_6D_6 at 298 K.

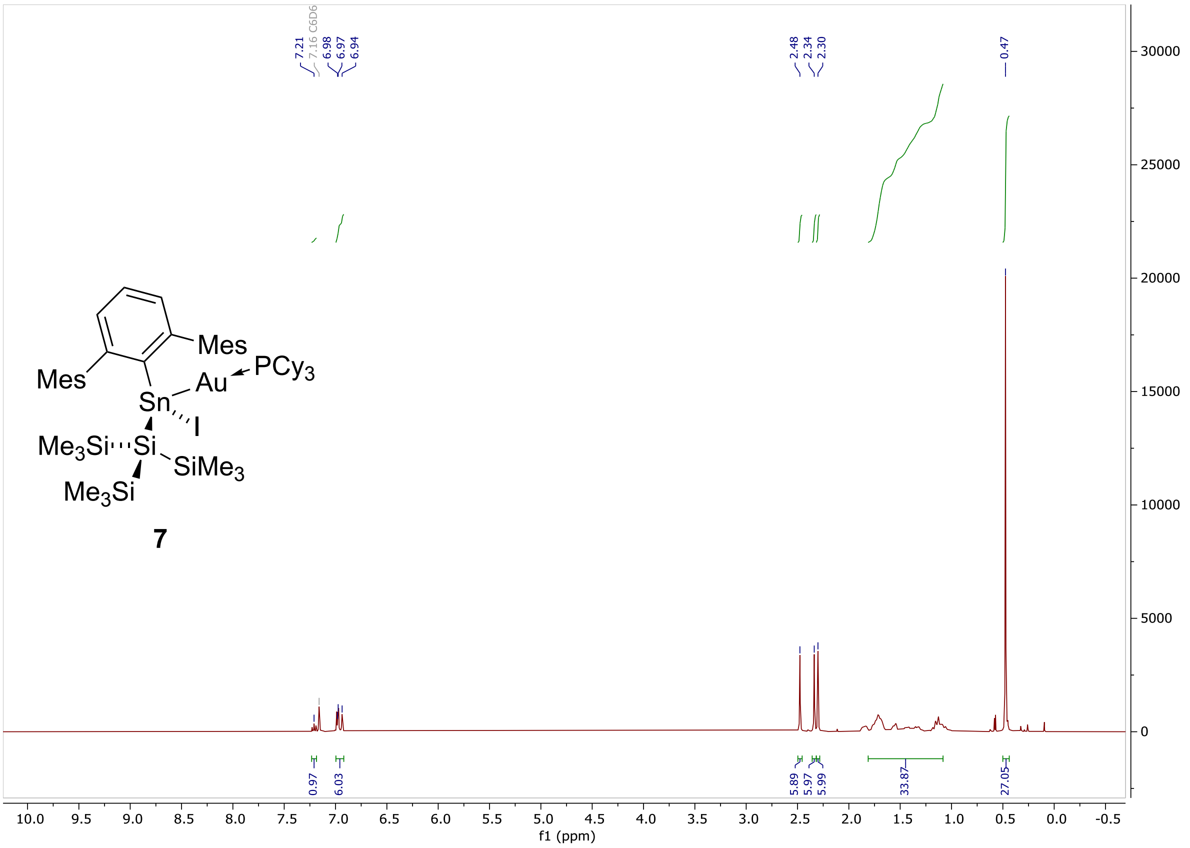

Figure S23. ^{1}H NMR spectrum of **7** in C_6D_6 at 298 K.

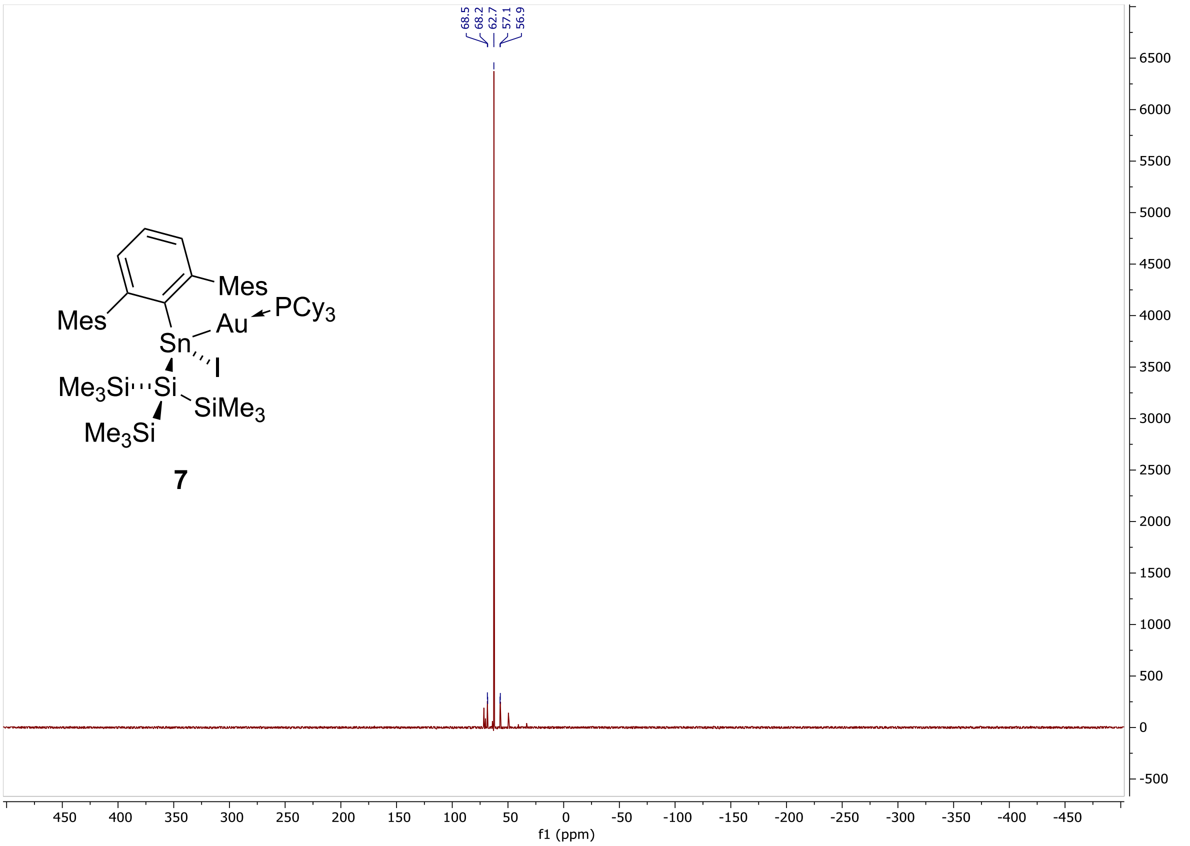

Figure S24. ^{31}P NMR spectrum of **7** in C_6D_6 at 298 K.

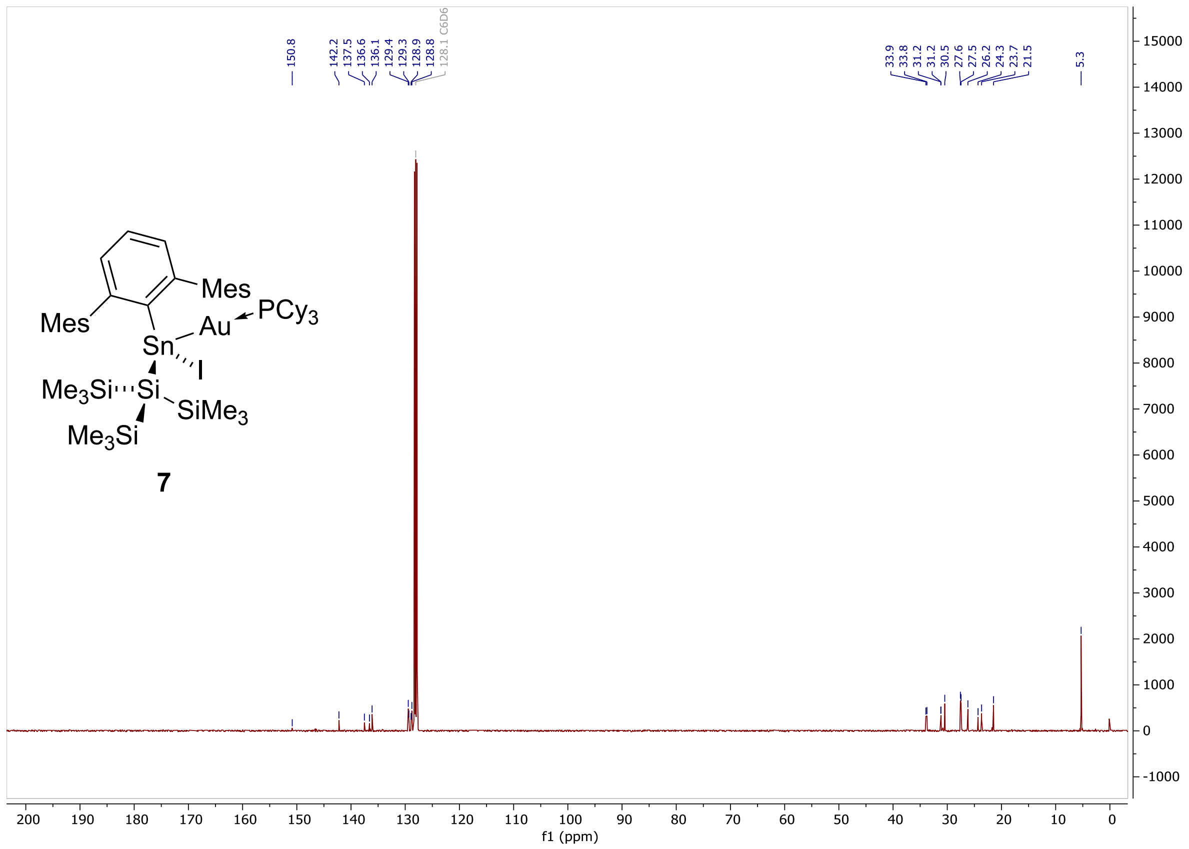

Figure S25. ^{13}C NMR spectrum of **7** in C_6D_6 at 298 K.

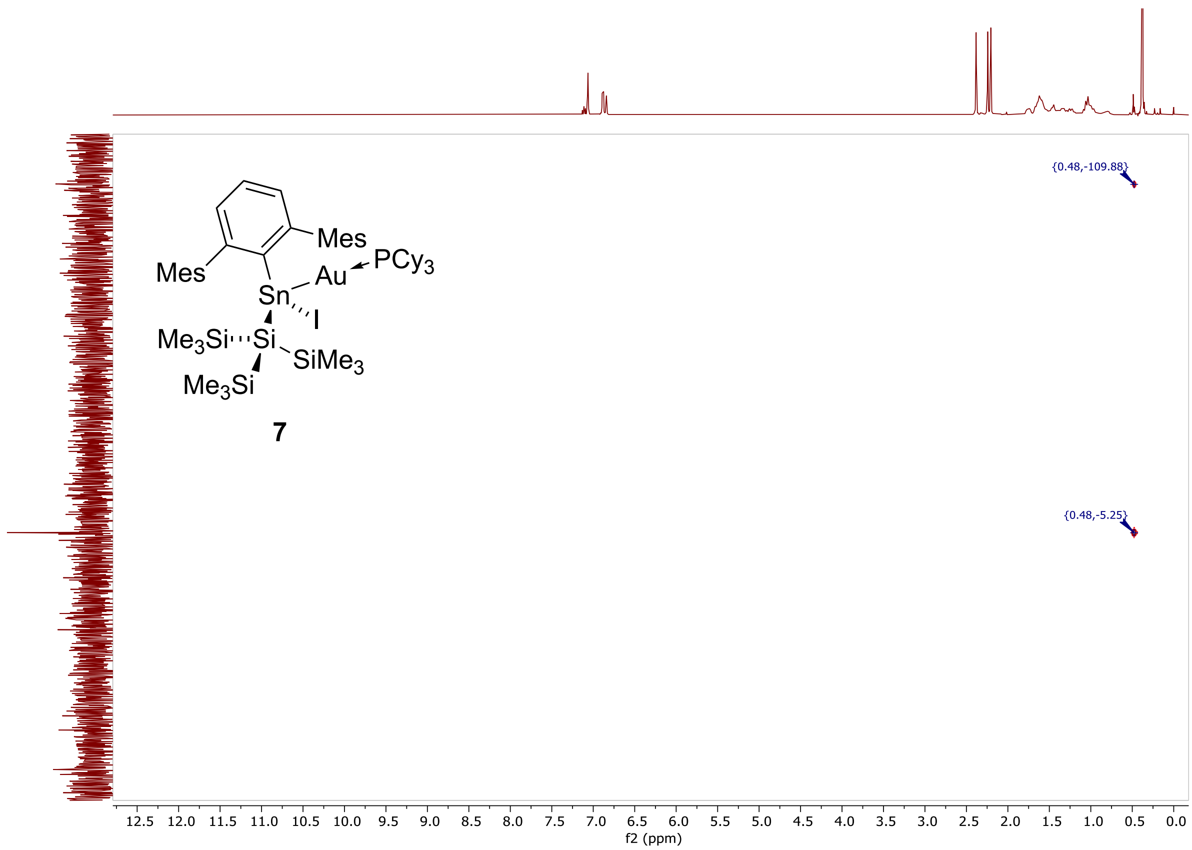

Figure S26. $^{29}Si/^{1}H$ HMBC spectrum of **7** in C_6D_6 at 298 K.

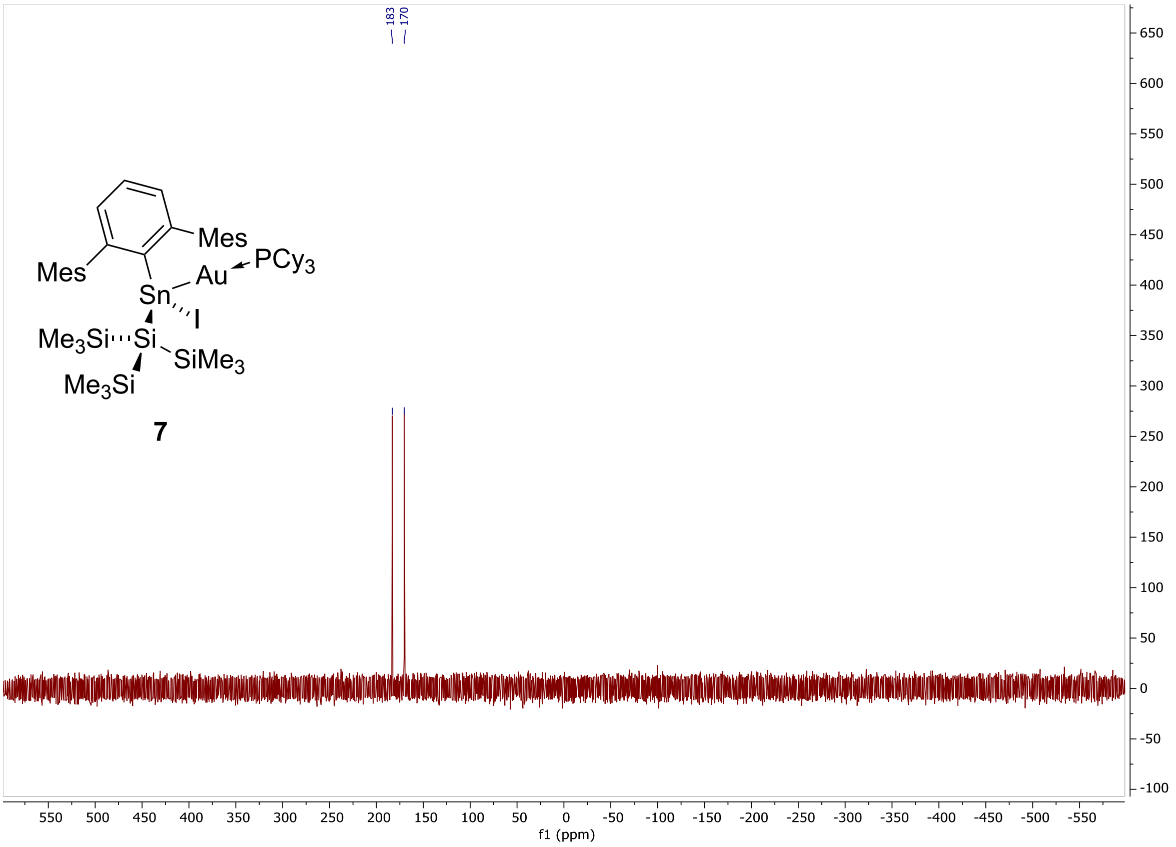

Figure S27. ^{119}Sn NMR spectrum of **7** in C_6D_6 at 298 K.

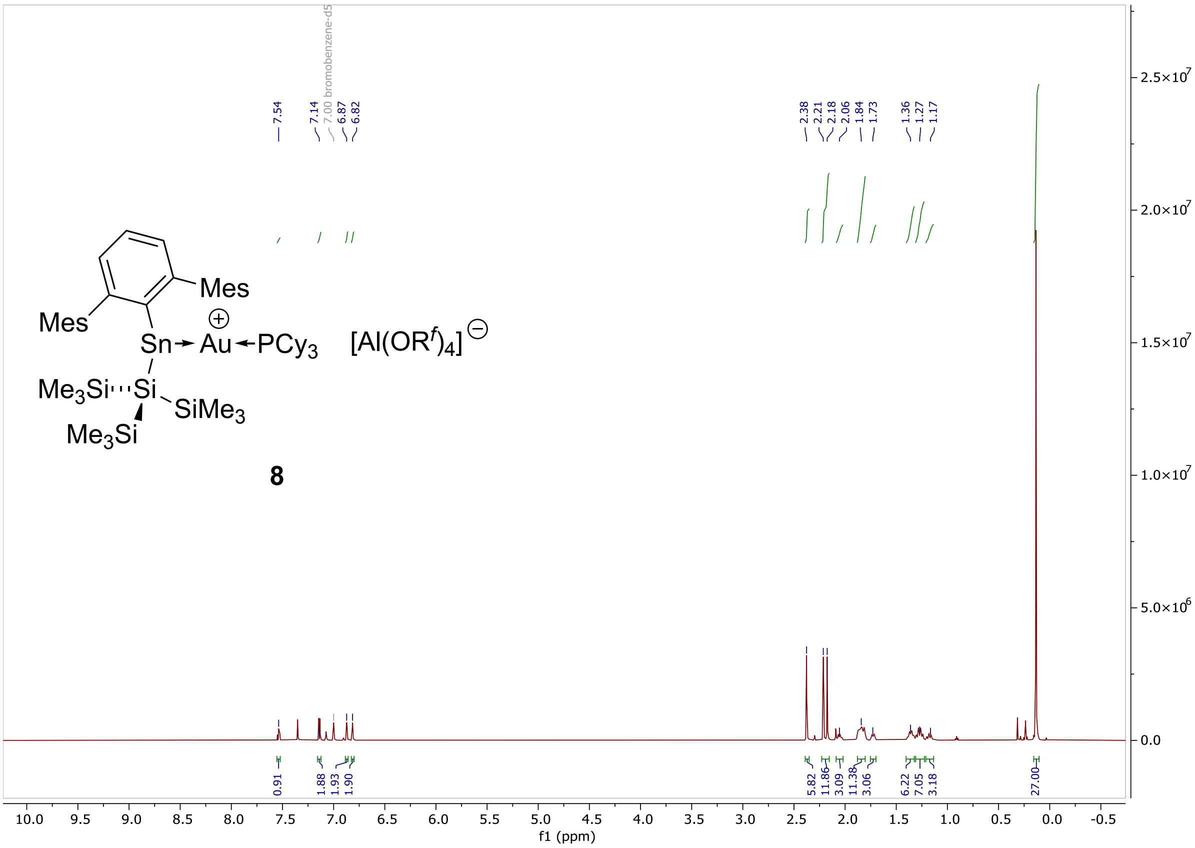

Figure S28. ^{1}H NMR spectrum of **8** in C_6D_5Br at 298 K.

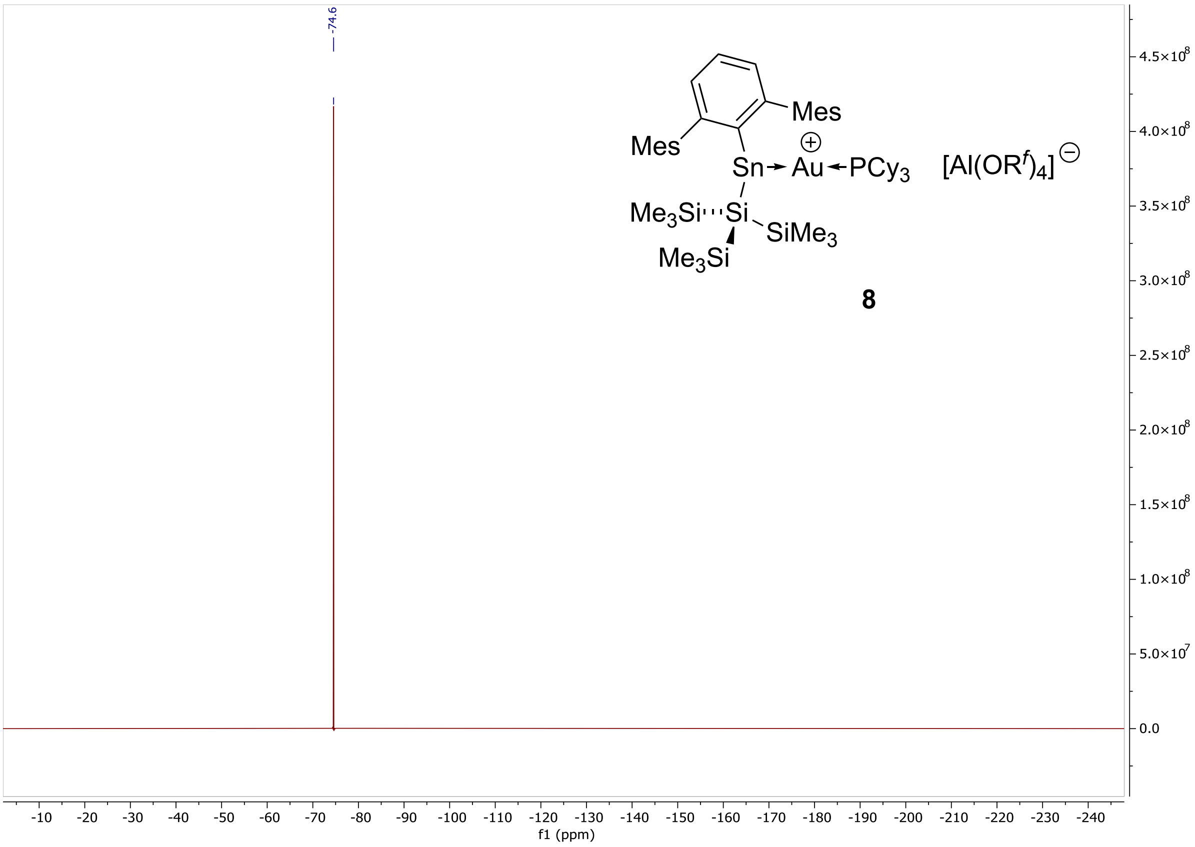

Figure S29. ^{19}F NMR spectrum of **8** in C_6D_5Br at 298 K.

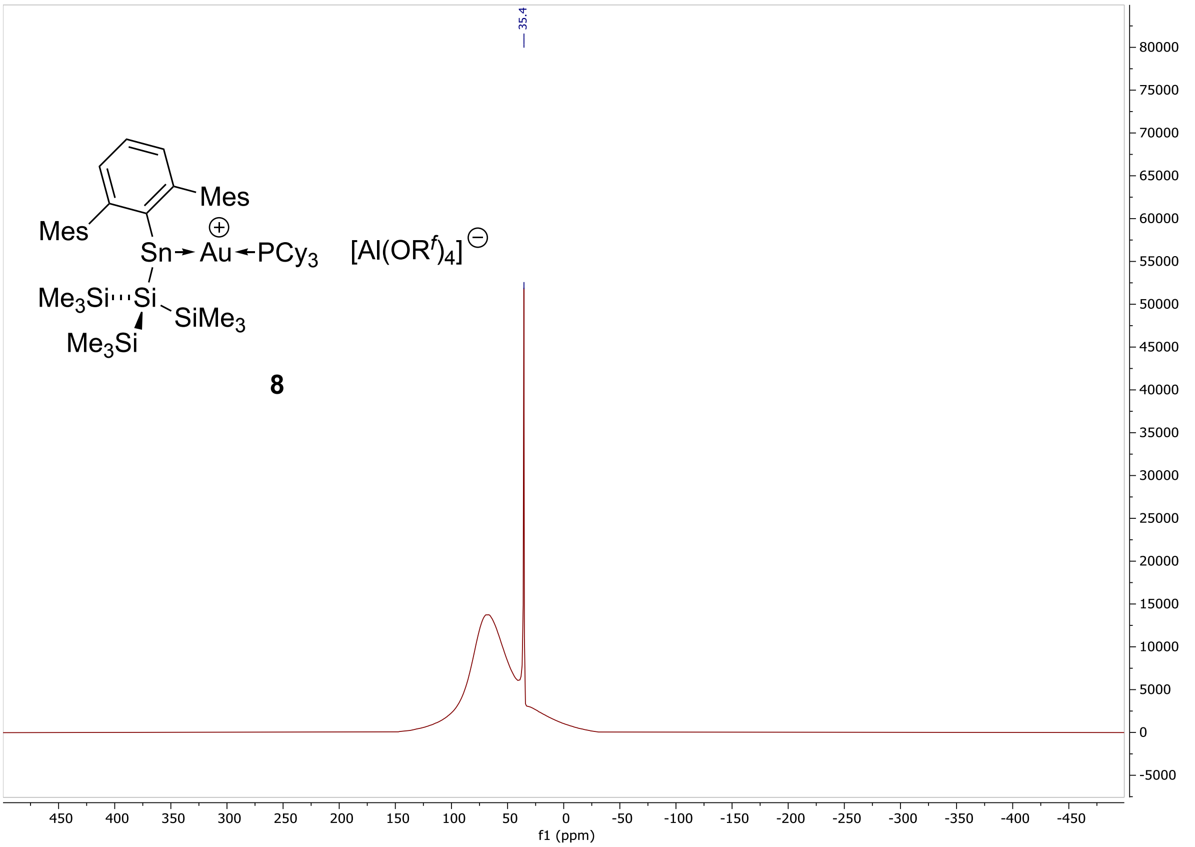

Figure S30. ^{27}Al NMR spectrum of **8** in C_6D_5Br at 298 K.

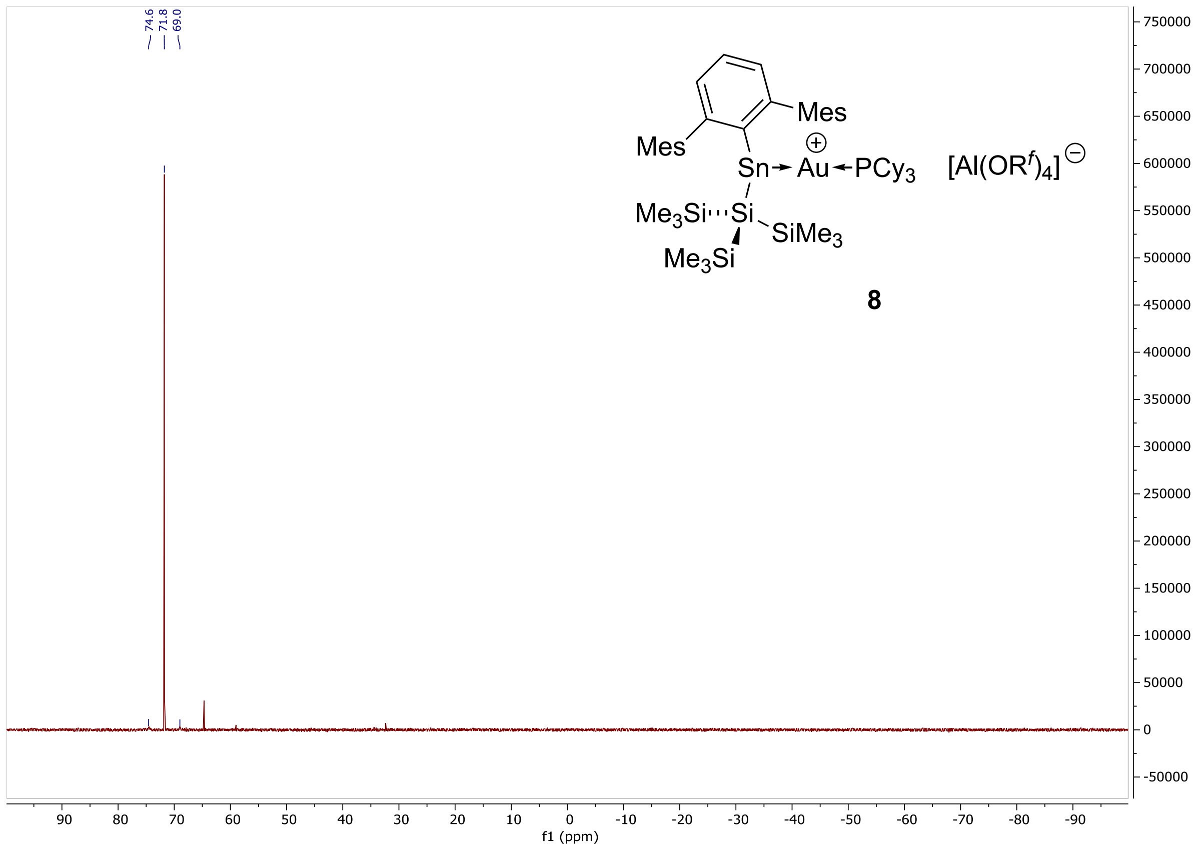

Figure S31. ^{31}P NMR spectrum of **8** in C_6D_5Br at 298 K.

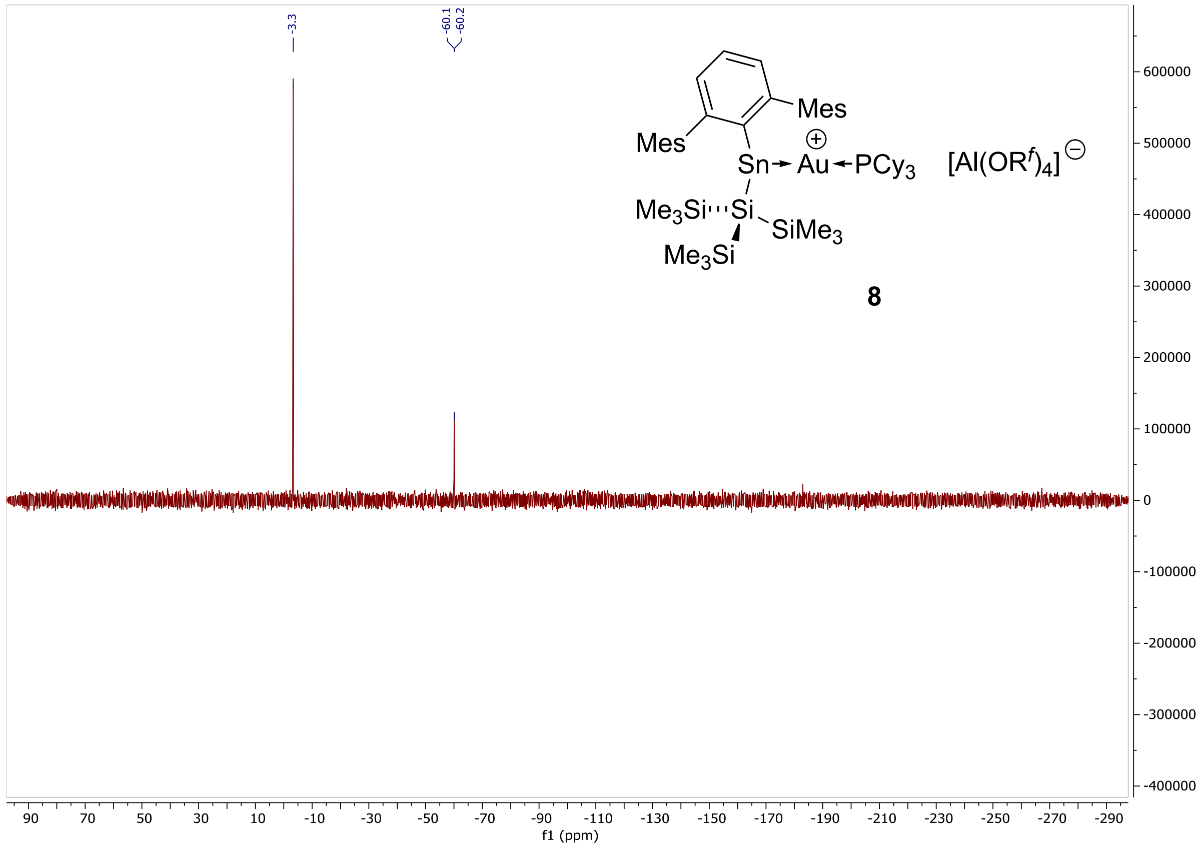

Figure S32. ^{29}Si NMR spectrum of **8** in C_6D_5Br at 298 K.

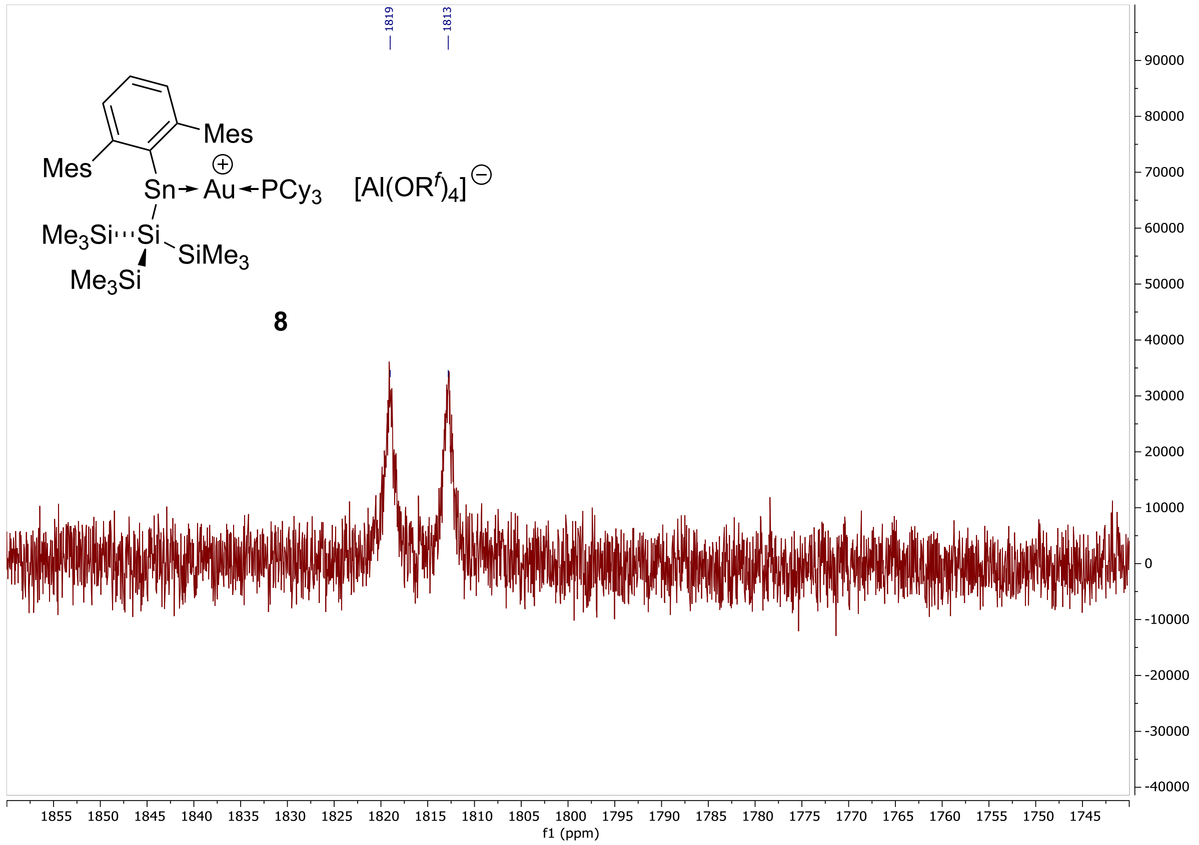

Figure S33. ^{119}Sn NMR spectrum of **8** in C_6D_5Br at 298 K.

6. Details of Quantum Chemical Calculations

Methods

All computational work reported herein was performed using ORCA (revision 6.1.0).[s11,s12] Geometry optimizations were carried out using the meta–generalized gradient approximation (mGGA) functional r²SCAN,[s13] in conjunction with the def2-TZVPPm basis set,[s14] including the D4 dispersion correction,[s15] and the geometrical counterpoise correction (gCP)[s16,s17] (collectively referred to as the r²SCAN-3c method).[s14]

Solvent effects were approximated using the conductor-like continuum polarization model (CPCM) with benzene as the solvent.[s18] The nature of the stationary point was confirmed by a full vibrational frequency calculation. The optimized structure exhibited no imaginary frequencies, consistent with a true minimum on the potential energy surface. Only this single compound was investigated computationally.

Energy Decomposition Analysis combined with Natural Orbitals for Chemical Valence (EDA-NOCV)[s19] was performed in ORCA at the same level of theory. The molecule was fragmented along the Au–Sn bond. The Sn fragment was defined with a charge of 0 and a multiplicity of 1, while the Au fragment was assigned a charge of +1 and a multiplicity of 1.

Electron Localization Function (ELF) surface and basin analyses were carried out using Multiwfn (version 3.8),[s20,s21] based on the ORCA-generated wavefunction. Natural Bond Orbital (NBO 7)[s20,s22] calculations were also performed using the ORCA wavefunction. All images were generated by ChimeraX (version 10.1).[s23,s24]

EDA NOCV

Table 1: EDA–NOCV decomposition energies for the Au–Sn bond using the fragmentation scheme described in the Methods section.

Energy Term	Energy / kcal mol^{-1}
Bond Energy	-81.87
Orbital Energy	-100.60
Electrostatic Energy	-59.69
Pauli Energy	162.03
Δ Exchange-Correlation	-77.36
Δ Dispersion	-6.96
Δ gCP correction	0.66

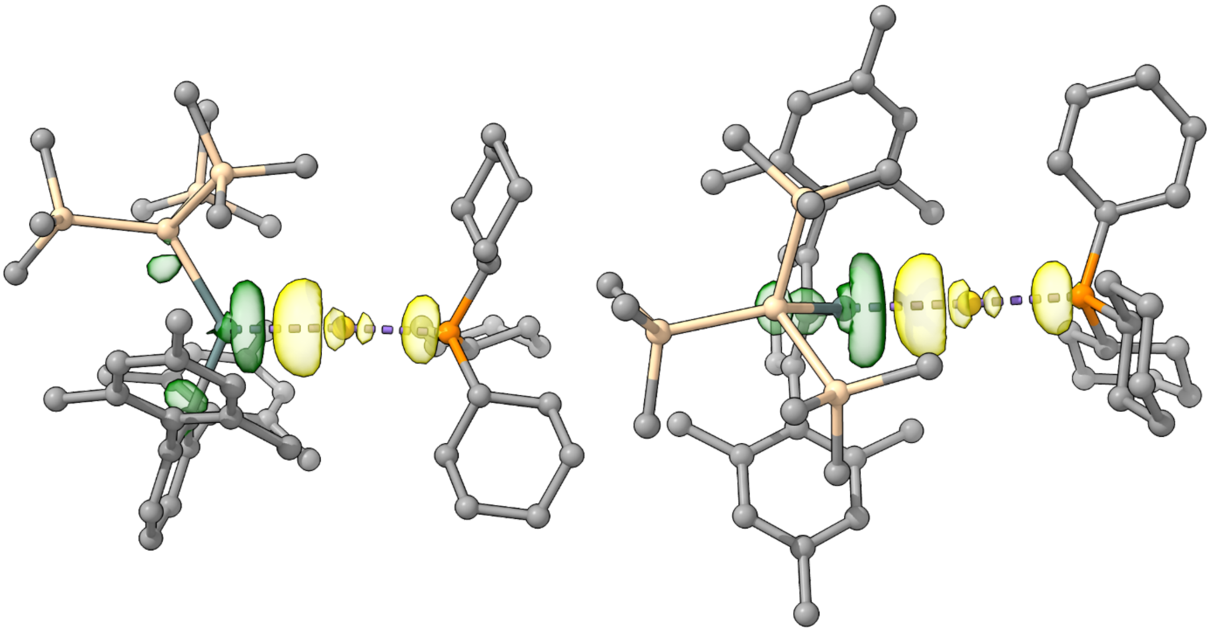

Figure S34. NOCV pair 1 deformation density (ΔE = −79.47 kcal mol^{-1}), plotted at an isovalue of ±0.005. Electron density flows from green to yellow, corresponding to a charge transfer of 0.81 e.

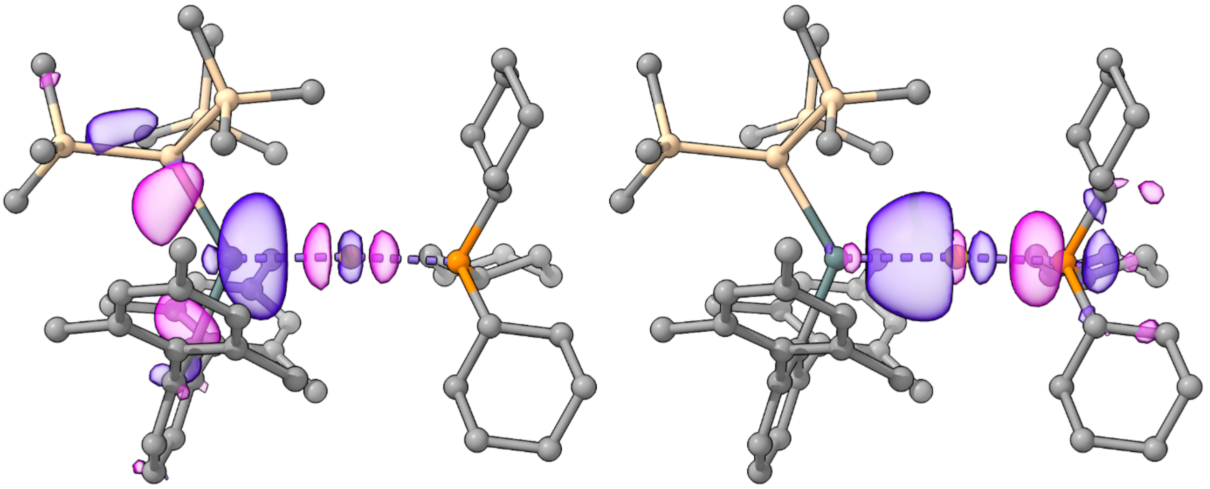

Figure S35. NOCV orbital pair 1 ($|v_1|$ = 0.81 e). The orbital on the left corresponds to the negative eigenvalue (ψ_{-1}), and the orbital on the right corresponds to the positive eigenvalue (ψ_{+1}). Orbitals are plotted at an isovalue of 0.05.

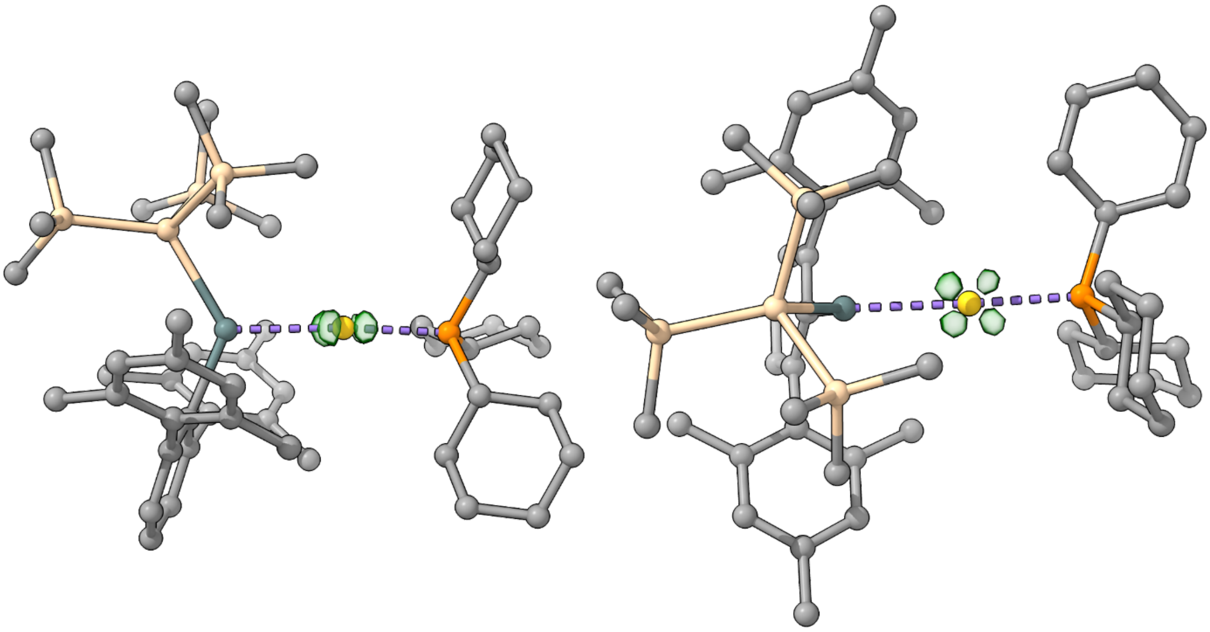

Figure S36. NOCV pair 2 deformation density ($\Delta E = -4.07$ kcal mol^{-1}), plotted at an isovalue of ±0.005. Electron density flows from green to yellow, corresponding to a charge transfer of 0.23 e.

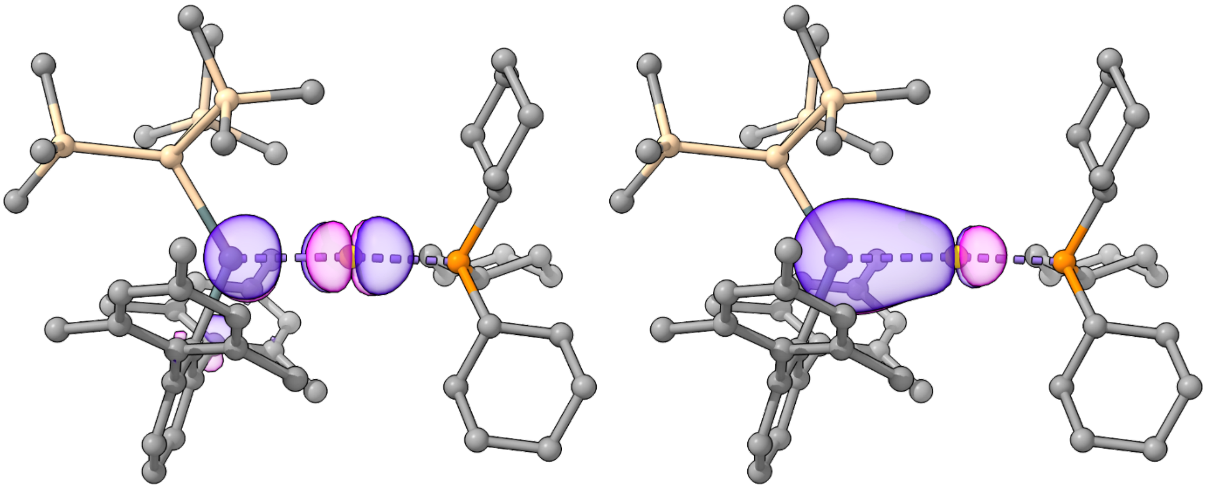

Figure S37. NOCV orbital pair 2 ($|v_2| = 0.23$ e). The orbital on the left corresponds to the negative eigenvalue (ψ_{-2}), and the orbital on the right corresponds to the positive eigenvalue (ψ_{+2}). Orbitals are plotted at an isovalue of 0.05.

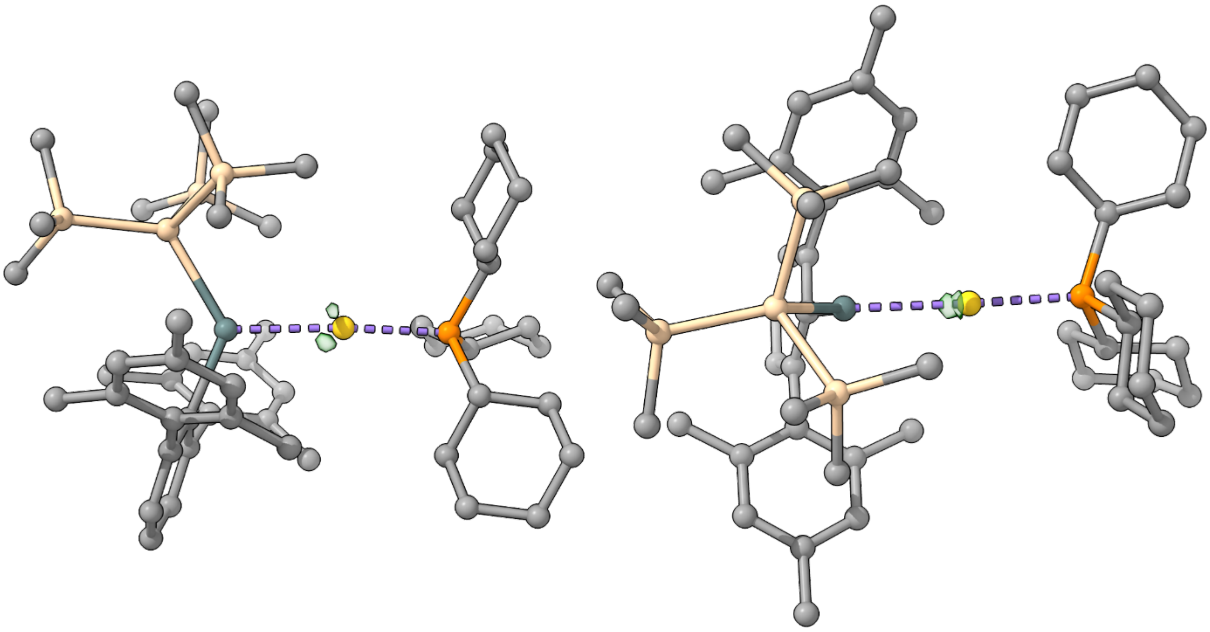

Figure S38.NOCV pair 3 deformation density (ΔE = −3.27 kcal mol^{-1}), plotted at an isovalue of ±0.005. Electron density flows from green to yellow, corresponding to a charge transfer of 0.14 e.

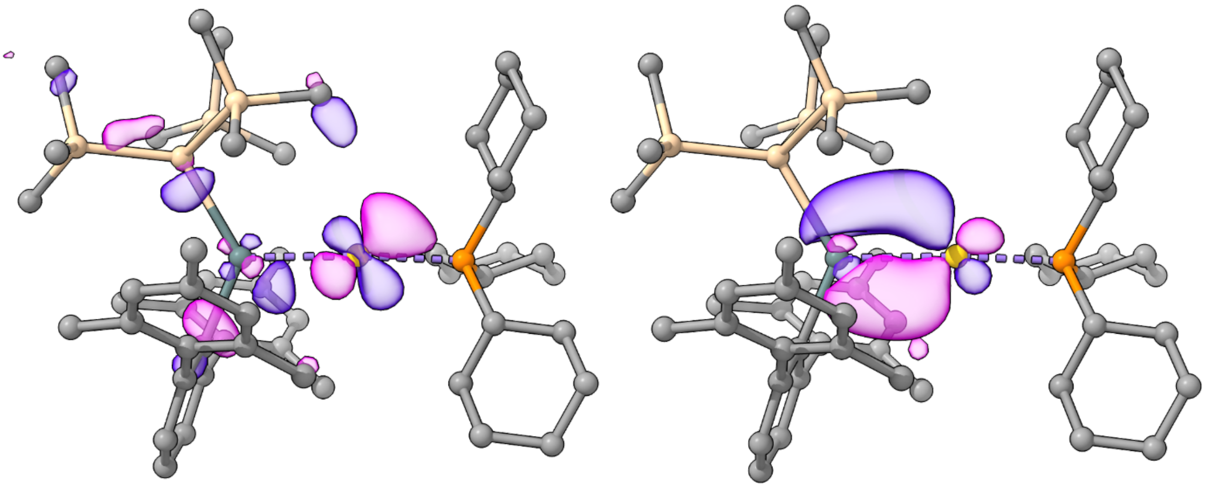

Figure S39. NOCV orbital pair 3 ($|v_3|$ = 0.14 e). The orbital on the left corresponds to the negative eigenvalue (ψ_{-3}), and the orbital on the right corresponds to the positive eigenvalue (ψ_{+3}). Orbitals are plotted at an isovalue of 0.05.

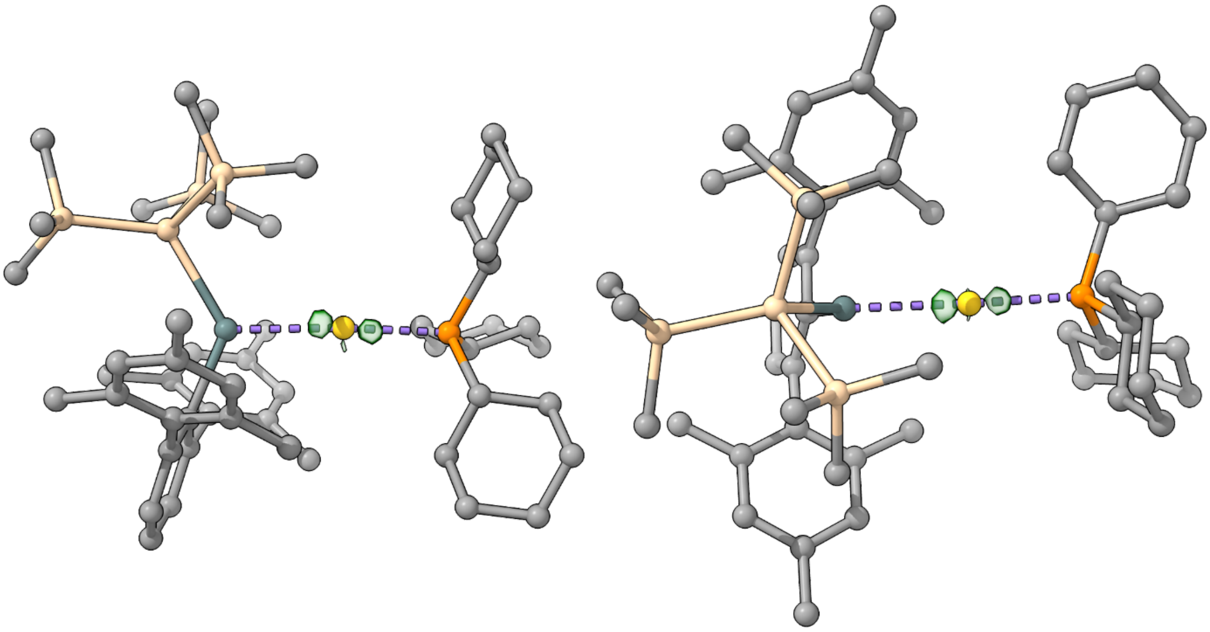

Figure S40. NOCV pair 4 deformation density (ΔE = −3.38 kcal mol^{-1}), plotted at an isovalue of ±0.005. Electron density flows from green to yellow, corresponding to a charge transfer of 0.12 e.

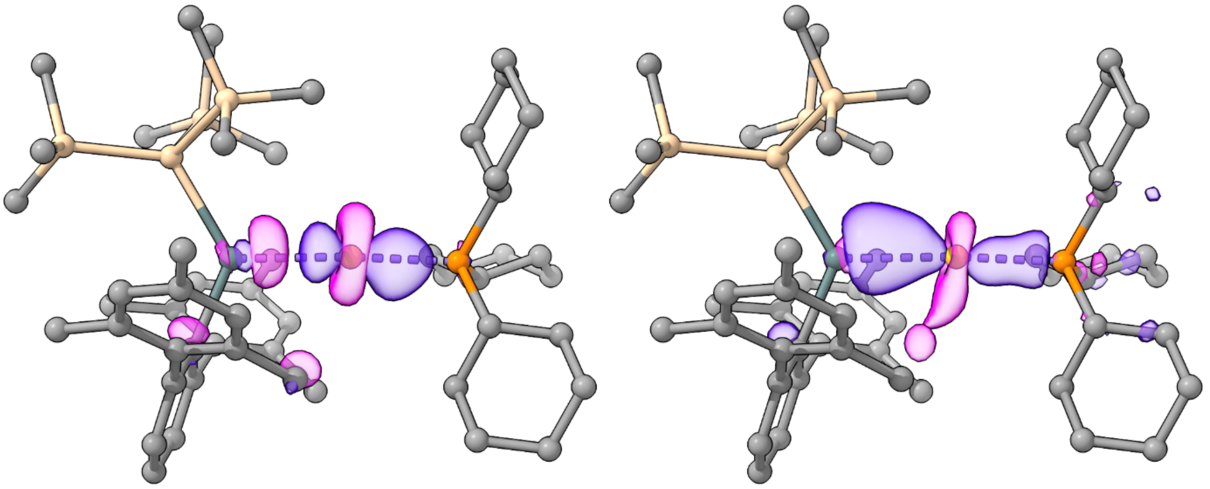

Figure S41. NOCV orbital pair 4 ($|v_4|$ = 0.12 e). The orbital on the left corresponds to the negative eigenvalue (ψ_{-4}), and the orbital on the right corresponds to the positive eigenvalue (ψ_{+4}). Orbitals are plotted at an isovalue of 0.05.

Orbitals

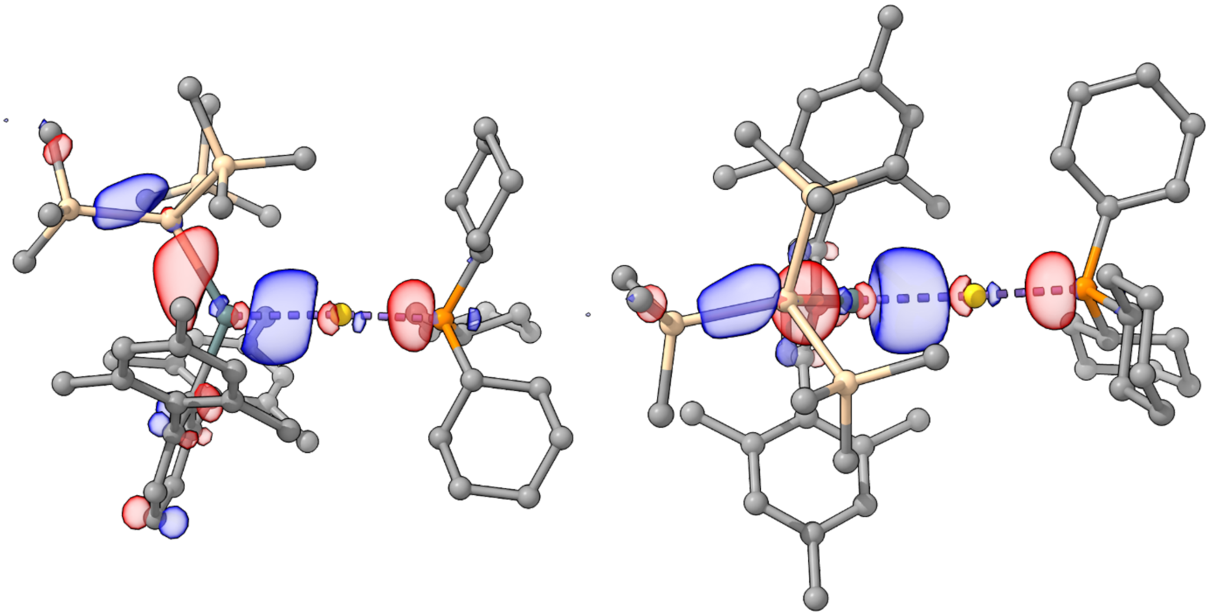

Figure S42. Highest occupied molecular orbital (HOMO, −6.54 eV) of the Au–Sn–P complex. The orbital is primarily associated with the Au–Sn–P bonding interaction. Orbitals are plotted at an isovalue of 0.05.

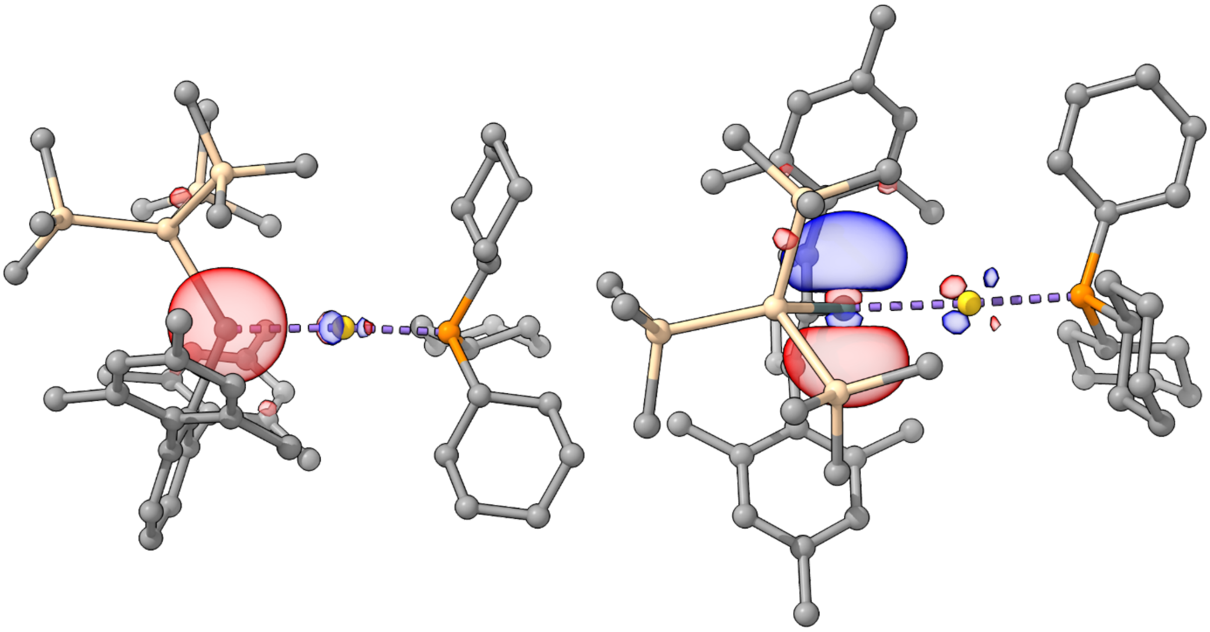

Figure S43. Lowest unoccupied molecular orbital (LUMO, −4.22 eV) of the Au–Sn–P complex. The orbital is predominantly localized at Sn and corresponds to a vacant p orbital. Orbitals are plotted at an isovalue of 0.05.

NBO

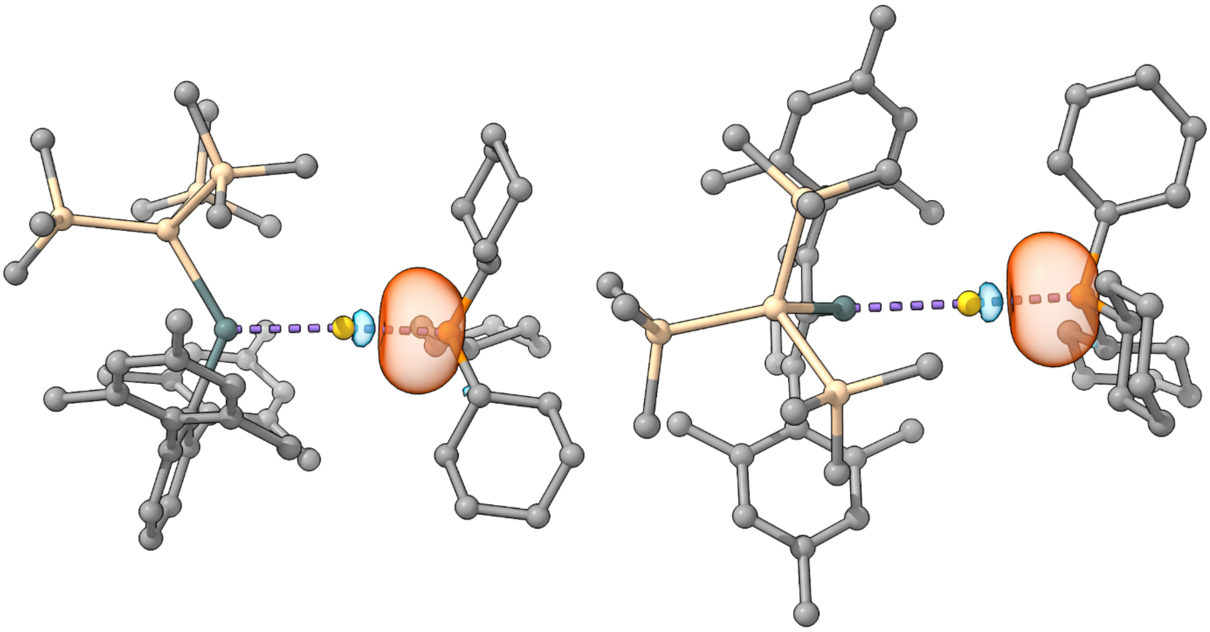

Figure S44. Natural bond orbital (NBO 95): lone pair at P (occupancy = 1.61 e). The orbital exhibits approximately sp hybrid character (48% s, 52% p). The orbital is plotted at an isovalue of 0.05.

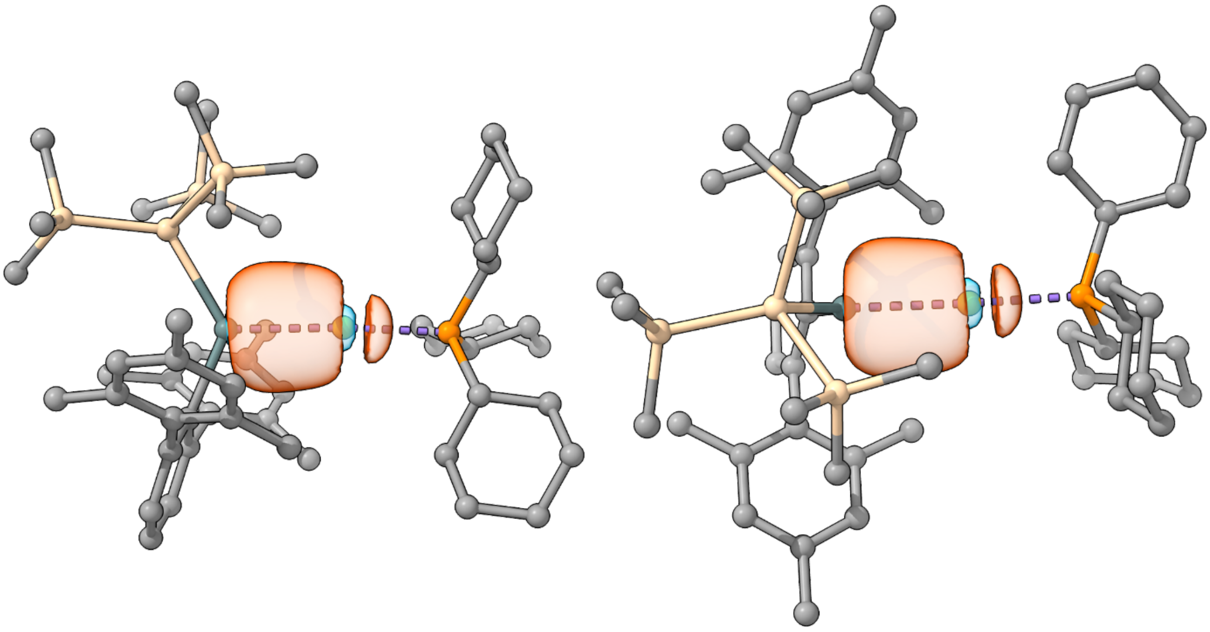

Figure S45. Natural bond orbital (NBO 96): σ-bonding orbital of the Au–Sn bond (occupancy = 1.87 e). The bond is composed of 42% Au character (predominantly s-type) and 58% Sn character. The Sn contribution exhibits approximately sp^2 hybridization (33.7% s, 66.2% p). Orbitals are plotted at an isovalue of 0.05.

ELF

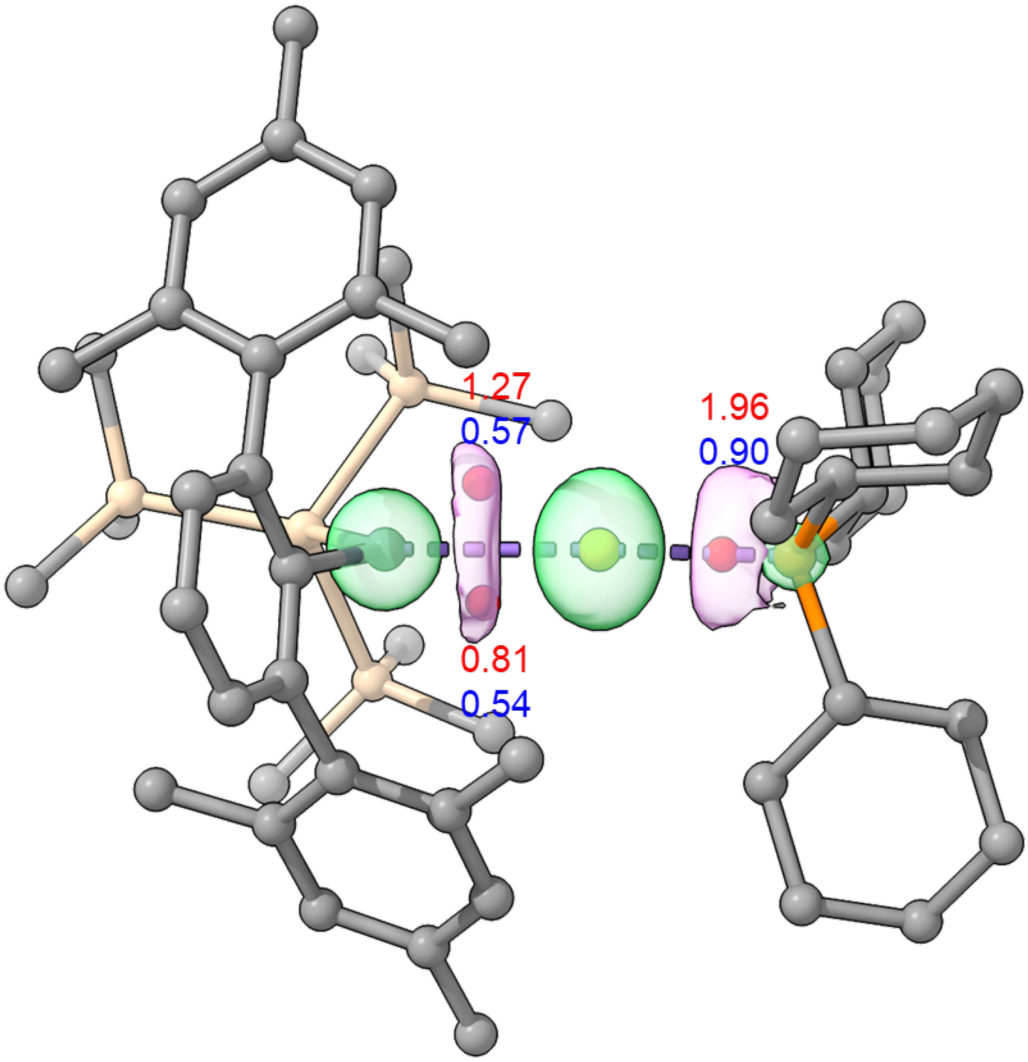

Figure S46. Electron localization function (ELF) surface plotted at an isovalue of 0.45, displaying disynaptic basins (green) and selected monosynaptic basins (pink). ELF attractors are indicated in red. Basin electron populations are labelled in red, and the corresponding ELF values at the attractors are given in blue.

7. References

s1. Fischer, M.; Roy, M. M. D.; Wales, L. L.; Ellwanger, M. A.; McManus, C.; Roper, A. F.; Heilmann, A.; Aldridge, S. Taming Heavier Group 14 Imine Analogues: Accessing Tin Nitrogen [Sn=N] Double Bonds and their Cycloaddition/Metathesis Chemistry. *Angew. Chem. Int. Ed.* **2022**, *61*, e202211616.

s2. Kunz, T.; Şahin, O.; Schnepf, A. Three different ways for the synthesis of the metalloid cluster $[Ge_9(Hyp^{tBuPh2})_3]^-$. *Z. Anorg. Allg. Chem.* **2022**, *648*, e202200081.

s3. Bott, R. C.; Bowmaker, G. A.; Buckley, R. W.; Healy, P. C.; Perera, M. C. S. Synthesis, Structures and Spectroscopic Properties of 1:1 Complexes of Gold(I) Halides with Tricyclohexylphosphine, $[Au(PCy_3)X]$, X = Cl, Br and I. *Aust. J. Chem.* **1999**, *52*, 271-278.

s4. Krossing, I. The Facile Preparation of Weakly Coordinating Anions: Structure and Characterisation of Silverpolyfluoroalkoxyaluminates $AgAl(OR_F)_4$, Calculation of the Alkoxide Ion Affinity. *Chem.-Eur. J.* **2001**, *7*, 490–502.

s5. Simons, R. S.; Pu, L.; Olmstead, M. M.; Power, P. P. Synthesis and Characterization of the Monomeric Diaryls $M\{C_6H_3\text{-}2,6\text{-}Mes_2\}_2$ (M = Ge, Sn, or Pb; Mes = 2,4,6-$Me_3C_6H_2$−) and Dimeric Aryl−Metal Chlorides $[M(Cl)\{C_6H_3\text{-}2,6\text{-}Mes_2\}]_2$ (M = Ge or Sn). *Organometallics* **1997**, *16*, 1920–1925.

s6. Hlina, J.; Stella, F.; Meshgi, M. A.; Marschner, C.; Baumgartner, J. σ-Bond Electron Delocalization in Oligosilanes as Function of Substitution Pattern, Chain Length, and Spatial Orientation. *Molecules* **2016**, *21*, 1079.

s7. Cosier, B. J.; Glazer, A. M. A Nitrogen-Gas-Stream Cryostat for General X-ray Diffraction Studies. *J. Appl. Cryst.* **1986**, *19*, 105–107.

s8. Sheldrick, G. M. SHELXT – Integrated space-group and crystal-structure determination. *Acta Crystallogr. A* **2015**, *71*, 3–8.

s9. Sheldrick, G. M. Crystal structure refinement with SHELXL. *Acta Crystallogr. C* **2015**, *71*, 3–8.

s10. Dolomanov, O. V.; Bourhis, L. J.; Gildea, R. J.; Howard, J. A. K.; Puschmann, H. OLEX2: a complete structure solution, refinement and analysis program. *J. Appl. Cryst.* **2009**, *42*, 339–341.

s11. Neese, F. Software Update: The ORCA Program System—Version 6.0. *WIREs Comput. Mol. Sci.* **2025**, *15*, e70019.

s12. Neese, F. The ORCA program system. *WIREs Comput. Mol. Sci.* **2012**, *2*, 73–78.

s13. Furness, J. W.; Kaplan, A. D.; Ning, J.; Perdew, J. P.; Sun, J. Accurate and Numerically Efficient r2SCAN Meta-Generalized Gradient Approximation. *J. Phys. Chem. Lett.* **2020**, *11*, 19, 8208–8215.

s14. Grimme, S.; Hansen, A.; Ehlert, S.; Mewes, J.-M. r2SCAN-3c: A 'Swiss army knife' composite electronic-structure method. *J. Chem. Phys.* **2021**, *154*, 064103.

s15. Caldeweyher, E.; Ehlert, S.; Hansen, A.; Neugebauer, H.; Spicher, S.; Bannwarth, C.; Grimme, S. A generally applicable atomic-charge dependent London dispersion correction. *J. Chem. Phys.* **2019**, *150*, 154122.

s16. Brandenburg, J. G.; Alessio, M.; Civalleri, B.; Peintinger, M. F.; Bredow, T.; Grimme, S. Geometrical Correction for the Inter- and Intramolecular Basis Set Superposition Error in Periodic Density Functional Theory Calculations. *J. Phys. Chem. A* **2013**, *117*, 9282–9292.

s17. Kruse, H.; Grimme, S. A geometrical correction for the inter- and intra-molecular basis set superposition error in Hartree-Fock and density functional theory calculations for large systems. *J. Chem. Phys.* **2012**, *136*, 154101.

s18. Barone, V.; Cossi, M. Quantum Calculation of Molecular Energies and Energy Gradients in Solution by a Conductor Solvent Model. *J. Phys. Chem. A* **1998**, *102*, 1995–2001.

s19. Sabando, R. C.; Riplinger, C.; Wennmohs, F.; Neese, F.; Bistoni, G. Broadening the Scope of the ETS-NOCV Scheme: A Versatile Implementation in ORCA. *J. Chem. Theory Comput.* **2025**, *21*, 7920–7934.

s20. Savin, A. The electron localization function (ELF) and its relatives: interpretations and difficulties. *J. Mol. Struct. THEOCHEM* **2005**, *727*, 127–131.

s21. Lu, T. A comprehensive electron wavefunction analysis toolbox for chemists, Multiwfn. *J. Chem. Phys.* **2024**, *161*, 082503.

s22. Glendening, E. D.; Badenhoop, J, K.; Reed, A. E.; Carpenter, J. E.; Bohmann, J. A.; Morales, C. M.; Karafiloglou, P.; Landis, C. R. Weinhold, F. NBO 7.0. Theoretical Chemistry Institute, University of Wisconsin, Madison **2018**.

s23. Meng, E. C.; Goddard, T. D.; Pettersen, E. F.; Couch, G. S.; Pearson, Z. J.; Morris, J. H.; Ferrin, T. E. UCSF ChimeraX: Tools for structure building and analysis. *Protein Sci.* **2023**, *32*, e4792.

s24. Pettersen, E. F. ; Goddard, T. D.; Huang, C. C.; Meng, E. C.; Couch, G. S.; Croll, T. I.; Morris, J. H.; Ferrin, T. E. UCSF ChimeraX: Structure visualization for researchers, educators, and developers. *Protein Sci.* **2021**, *30*, 70–82.
